# RNA_seq_ and quantitative proteomic analysis of *Dictyostelium* knock-out cells lacking the core autophagy proteins ATG9 and/or ATG16

**DOI:** 10.1186/s12864-021-07756-2

**Published:** 2021-06-15

**Authors:** Qiuhong Xiong, Ning Song, Ping Li, Sarah Fischer, Roman Konertz, Prerana Wagle, Gernot Glöckner, Changxin Wu, Ludwig Eichinger

**Affiliations:** 1 Institutes of Biomedical Sciences, Key Laboratory of Chemical Biology and Molecular Engineering of Ministry of Education, Shanxi University, No. 92 Wucheng Road, 030006 Taiyuan, China; 2 Center for Biochemistry, Medical Faculty, University of Cologne, Joseph-Stelzmann-Str. 52, 50931 Cologne, Germany; 3 Cologne Excellence Cluster on Cellular Stress Responses in Aging Associated Diseases, University of Cologne, Joseph-Stelzmann-Str. 26, 50931 Cologne, Germany

**Keywords:** Autophagy, ATG9, ATG16, RNA_seq_, proteome, tandem mass tag (TMT), parallel reaction monitoring (PRM), *Dictyostelium discoideum*

## Abstract

**Background:**

Autophagy is an evolutionary ancient mechanism that sequesters substrates for degradation within autolysosomes. The process is driven by many autophagy-related (ATG) proteins, including the core members ATG9 and ATG16. However, the functions of these two core ATG proteins still need further elucidation. Here, we applied RNA_seq_ and tandem mass tag (TMT) proteomic approaches to identify differentially expressed genes (DEGs) and proteins (DEPs) in *Dictyostelium discoideum* ATG9‾, ATG16‾ and ATG9‾/16‾ strains in comparison to AX2 wild-type cells.

**Result:**

In total, we identified 332 (279 up and 53 down), 639 (487 up and 152 down) and 260 (114 up and 146 down) DEGs and 124 (83 up and 41 down), 431 (238 up and 193 down) and 677 (347 up and 330 down) DEPs in ATG9‾, ATG16‾ and ATG9‾/16‾ strains, respectively. Thus, in the single knock-out strains, the number of DEGs was higher than the number of DEPs while in the double knock-out strain the number of DEPs was higher. Comparison of RNA_seq_ and proteomic data further revealed, that only a small proportion of the transcriptional changes were reflected on the protein level. Gene ontology (GO) analysis revealed an enrichment of DEPs involved in lipid metabolism and oxidative phosphorylation. Furthermore, we found increased expression of the anti-oxidant enzymes glutathione reductase (gsr) and catalase A (catA) in ATG16‾ and ATG9‾/16‾ cells, respectively, indicating adaptation to excess reactive oxygen species (ROS).

**Conclusions:**

Our study provides the first combined transcriptome and proteome analysis of ATG9‾, ATG16‾ and ATG9‾/16‾ cells. Our results suggest, that most changes in protein abundance were not caused by transcriptional changes, but were rather due to changes in protein homeostasis. In particular, knock-out of *atg9* and/or *atg16* appears to cause dysregulation of lipid metabolism and oxidative phosphorylation.

**Supplementary Information:**

The online version contains supplementary material available at 10.1186/s12864-021-07756-2.

## Background

Autophagy is an evolutionary ancient mechanism for the recycling of cellular material. In this process, portions of the cytosol are engulfed within a newly formed double-membrane vesicle, the autophagosome, and delivered to the lysosome for degradation [1]. Since the identification of the first autophagy-related (*ATG*) gene in yeast by Ohsumi in 1993 [2], significant progress has been made in understanding the molecular mechanisms of autophagy [3, 4]. To date, more than 50 ATG genes have been identified [4] and around 20 of them, including ATG9 and ATG16, constitute the “core” autophagic machinery, as they are required for autophagosome formation in all autophagy subtypes [1, 5, 6]. ATG9, the only transmembrane core autophagy protein, cycles between different organelle compartments via vesicular transport pathways and delivers membrane lipids to the autophagosome formation site in response to induction of autophagy [7]. ATG16 acts in the hetero-tetrameric ATG16/ATG12 ~ 5 complex, which determines the ATG8 lipidation sites and facilitates transfer of ATG8 from ATG3 to phosphatidylethanolamine (PE) [6, 8]. Apart from their autophagic functions, most of the ATGs are also involved in different autophagy-independent processes [9]. For example, ATG9 functions in the transport of lysosomal hydrolases from the trans-Golgi network (TGN) in mammalian cells [10] and regulates the actin cytoskeleton organization through interactions with profilin and Ena in *Drosophila* [11]. Also for ATG16 several autophagy-independent functions, as for example in antigen presentation, in hormone secretion, and in plasma membrane repair have been reported [6].

The social amoeba *Dictyostelium discoideum* is a well-established model organism to study the autophagic process [7]. Under nutrient rich conditions, *Dictyostelium* cells grow as separate, independent cells that divide by binary fission and feed on bacteria by phagocytosis [12]. Upon starvation solitary amoebae aggregate and undergo distinct morphological states, finally giving rise to a mature fruiting body, which is composed of a ball of spores supported by a thin, long stalk made of vacuolized dead cells [13]. Development takes place in the absence of nutrients and *Dictyostelium* cells must mobilize a large fraction of the required energy for morphogenesis and associated biosynthetic pathways by autophagy [7]. As a consequence, knock-out mutants of core autophagy genes generally suffer from developmental defects [14]. We have previously shown that the single and double *atg9* and *atg16* knock-out mutants suffered from pleiotropic defects, which were comparable for some cellular processes in all three mutant strains but for other processes more severe in the double mutant. While defects in macropinocytosis and phagocytosis were comparable in the single and double mutants, we found that, autophagosome formation, multicellular development and proteasomal activity were much more severely impaired in the double knock-out cells [15–17]. We concluded from these results that ATG9 and ATG16 probably function in different pathways in addition to autophagy [16, 17].

In this study, we used RNA_seq_ and Tandem Mass Tag (TMT) proteomic approaches to investigate global transcriptome and proteome changes of ATG9‾, ATG16‾ and ATG9‾/16‾ strains in comparison to AX2 wild-type cells. We found 332 (279 up and 53 down), 639 (487 up and 152 down) and 260 (114 up and 146 down) differentially expressed genes (DEGs) in ATG9‾, ATG16‾ and ATG9‾/16‾ strains, respectively. On the protein level we found 124 (83 up and 41 down), 431 (238 up and 193 down) and 677 (347 up and 330 down) differentially expressed proteins (DEPs) in ATG9‾, ATG16‾ and ATG9‾/16‾ strains, respectively. Comparison of RNA_seq_ and proteomic data showed that only a small proportion of the transcriptional changes were reflected in corresponding changes on the protein level. This suggests that most proteome changes were not caused by transcriptional changes but were rather due to changes in protein homeostasis. Gene Ontology (GO) analysis of DEPs revealed that metabolism, in particular lipid metabolism and oxidative phosphorylation appear disturbed in mutant strains. In conclusion, this study provides the first analysis of global transcriptomic and proteomic changes in ATG9 and/or ATG16 deficient *Dictyostelium* cells and adds to our knowledge of the molecular regulatory network of ATG9 and ATG16.

## Results

### Autophagy mutants display massive transcriptional changes

**Fig. 1:**
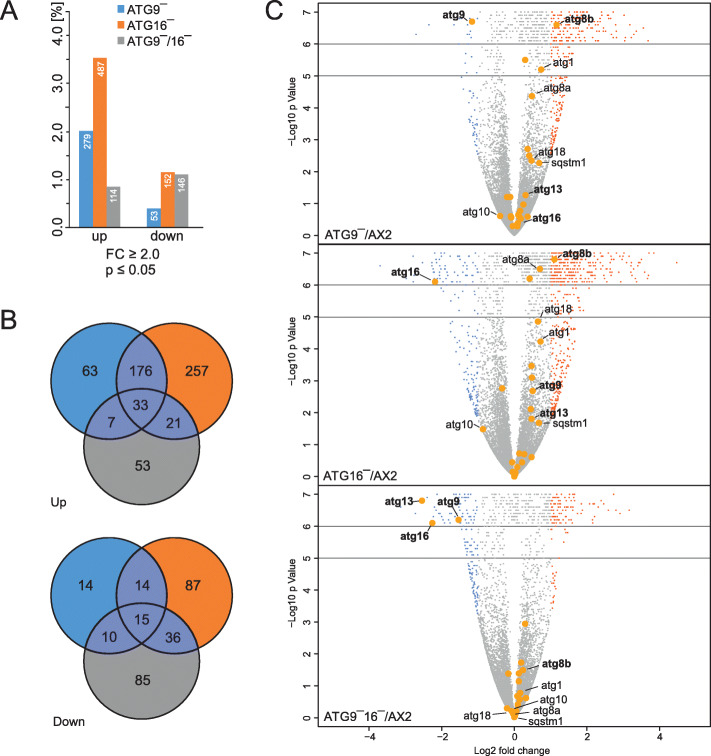
Differentially regulated genes in comparisons of ATG9‾, ATG16‾, and ATG9‾/16‾ strains with AX2. (**A**) Percentage of up- and downregulated genes in dependence of thresholds for fold change (2.0 ≥ FC ≤ 0.5) and *p*value ≤ 0.05 are shown. RNA was isolated from vegetative cells and nine biological replicates of each strain were analyzed. Transcriptome: 13,729 transcription units. (**B**) Venn diagrams of differentially regulated genes of ATG9‾ versus AX2 (left circle, blue), ATG16‾ versus AX2 (right circle, orange), and ATG9‾/16‾ versus AX2 (lower circle, grey). Differentially regulated genes common for two or three comparisons are shown in dark blue. Top, upregulated and bottom, downregulated genes. Only those genes with fold change ≥ 2.0 or ≤ 0.5 and *p* ≤ 0.05 were used as input. (**C**) Presentation of differentially regulated genes in a volcanoplot for each strain comparison. Differentially regulated genes with Log2 fold change ≥ 1 and *p* ≤ 0.05 are labelled red, genes with Log2 fold change ≤ − 1 and *p* ≤ 0.05 are labelled blue, differentially regulated autophagy related genes are highlighted by bigger orange-filled circles. For better visualization, all genes with a *p*-value between 10^− 5^ and 10^− 6^ are randomly distributed in the area from 10^–5^ to 10^–6^ and all genes with a *p-value* = 0 (i.e. values < 10^− 6^) are randomly distributed in the area from 10^–6^ and 10^–7^. Autophagy genes with a fold change ≥ 1.5 or ≤ 0.67 and *p* ≤ 0.05 in at least one of the comparisons are indicated by their Demerec name. The plot was created using the R environment (v. 2.15.0)

We performed RNA_seq_ to determine genome-wide transcriptional changes of ATG9‾, ATG16‾ and ATG9‾/16‾ cells in comparison to wild-type AX2. First, we compared the differential regulation of the entire transcriptome of the different mutant strains to AX2 in dependence of different thresholds for *p*-value. The analysis revealed for fold changes (FC) ≥ 2.0 and for *p*-values ≤ 0.01 versus ≤ 0.05 only a marginal decrease for the number of up-regulated genes for the ATG9‾ strain, while no reduction in the number of differentially regulated genes was seen for all other comparisons. Therefore, we performed further analyses for genes with an FC ≥ 2.0 and a p-value ≤ 0.05. The DEGs are listed in Table S1. We observed 3- to 5-fold higher numbers of up- than of down-regulated genes for ATG16‾ and ATG9‾ cells, respectively, while for ATG9‾/16‾ cells the number of down-regulated genes was slightly higher than the number of up-regulated genes. In total, 487 (3.55 %) up- and 152 (1.11 %) down-regulated genes were reported for ATG16‾ cells and 279 (2.03 %) and 53 (0.39 %), respectively, for ATG9‾ cells. For ATG9‾/16‾ only 114 (0.83 %) up-, but 146 (1.06 %) down-regulated genes were reported (Fig. 1 A, Table S1). The very low number of up-regulated genes in the ATG9‾/16‾strain suggests independent, and for a subset of genes also opposite, regulation in the ATG9‾ and the ATG16‾ strains. We further compared the up- and down-regulated genes of all three mutant strains graphically in Venn diagrams. The absolute number of up-regulated genes common to either two strains was very similar for the ATG9‾ and ATG9‾/16‾ and the ATG16‾ and ATG9‾/16‾ strains, while the ATG9‾ and ATG16‾ strains shared considerably more genes (Fig. 1B). If we look at the percentages of common differentially up-regulated genes, we find that ATG9‾ cells share 75 % of the up-regulated genes with ATG16‾ cells, and, vice versa, ATG16‾ cells 43 % of the up-regulated genes with ATG9‾ cells. In contrast, ATG9‾ cells share only 14 % and ATG16‾ cells only 11 % of the up-regulated genes with ATG9‾/16‾ cells. For the down-regulated genes the proportion of genes shared with ATG9‾/16‾ cells is much higher. Here, ATG9‾ shares 47 % and ATG16‾ 34 % with ATG9‾/16‾ cells (Table S2). In total, 610 genes were up- and 261 were down-regulated in either one, two, or all three strains. Of these genes, the three strains had only 33 (3.8 %) of the up- and 15 (4.3 %) of the down-regulated genes in common (Fig. 1B).

To further analyze the differentially regulated genes we used volcano-plots, where statistical significance versus differential regulation of RNA_seq_ data is plotted [18]. In the generated plots for the three knock-out strains, significantly up- and down-regulated genes (FC ≥ 2.0, *p*-value ≤ 0.05) are displayed as red and blue dots, respectively, and un-regulated genes as grey dots (Fig. 1 C). Two findings are obvious: (i) the much higher fraction of up-regulated genes in the ATG9‾ and ATG16‾ cells and (ii) the significantly higher number of up-regulated genes in the ATG9‾ and ATG16‾ strains and the much lower number in the ATG9‾/16‾ strain. We highlighted genes encoding core autophagy proteins (orange circles) and added the gene names for those genes that displayed a 0.67 ≥ FC ≥ 1.5 and a *p*-value ≤ 0.05 in at least one of the knock-out strains (Fig. 1 C). We found that four of these genes, namely *atg1*, *atg8a*, *atg8b*, and *atg18*, were significantly up-regulated in ATG16‾ and *atg1* and *atg8b* in ATG9‾ cells. Of these only *atg8b* was more than 2-fold up-regulated in both strains. However, none of these four genes was up-regulated in ATG9‾/16‾ cells. As expected, *atg9* and *atg16* were down-regulated in the corresponding knock-out strains. In addition, *atg13* was strongly down-regulated in the ATG9‾/16‾ strain, *atg10* in ATG16‾ cells and *sqstm1* (**S**e**Q**ue**ST**oso**M**e-**1** also known as p62) was up-regulated in the ATG9‾ and ATG16‾ strains (Fig. 1 C, Table 1).

**Table 1:** Differentially regulated autophagy genes

Gene ID	Gene Name	ATG9‾		ATG16‾		ATG9‾/16‾	
		**FC**	***p*-value**	**FC**	***p*-value**	**FC**	***p*-value**
DDB_G0292390	*atg1*	1.67	0.000	1.65	0.000	1.12	0.167
DDB_G0286191	*atg8a*	1.40	0.000	1.63	0.000	0.98	0.756
DDB_G0290491	*atg8b*	2.24	0.000	2.15	0.000	1.18	0.032
DDB_G0285375	*atg18*	1.39	0.004	1.57	0.000	0.95	0.621
DDB_G0285323	*atg9*	0.45	0.000	1.42	0.002	0.34	0.000
DDB_G0275323	*atg16*	1.06	0.432	0.22	0.000	0.21	0.000
DDB_G0268840	*atg10*	0.76	0.245	0.55	0.033	0.87	0.505
DDB_G0269162	*atg13*	1.24	0.054	1.39	0.016	0.17	0.000
DDB_G0270098	*sqstm1*	1.60	0.005	1.55	0.024	0.97	0.860

*FC* fold change

The up- and down-regulated genes of each of the three mutant strains were separately analyzed for the enrichment of genes in GO categories of the biological process, molecular function and cellular component ontologies with the program Panther [19]. Given a gene and a reference list, the program calculates the enrichment and statistical significance of every GO term by comparing the observed number of genes or gene products in a specific category with the number of genes or gene products that might appear in the same category if a selection performed from the same reference list were completely random. For the up-regulated genes, there was no enrichment of genes in any category of the three ontologies reported for ATG9‾ and ATG9‾/16‾ cells. For ATG16‾ cells genes involved in “pyrimidine ribonucleoside metabolic process” in the biological process ontology were enriched 16-fold and genes linked to “membrane” in the cellular component ontology were 1.3-fold enriched (Table S3). Considering the high number of up-regulated genes, in particular in ATG9‾ and ATG16‾ cells, this result was unexpected. For the down-regulated genes, there was no enrichment of genes in any category of the three ontologies reported for ATG9‾ cells. For ATG16‾ and ATG9‾/16‾ cells genes involved in “DNA integration” and “sexual reproduction” in the biological process ontology were enriched more than 10-fold and more than 5-fold, respectively (Table S3). The former enrichment was due to the down-regulation of mainly *gag* genes from retrotransposons, the latter by the down-regulation of mainly genes encoding proteins with EGF-like domains. Furthermore, the analysis revealed for ATG9‾/16‾ cells in the cellular component ontology an enrichment of genes encoding “anchored components of membrane” (Table S3). All five down-regulated genes encode ponticulin-like proteins. Ponticulin is a *D. discoideum* membrane protein that links the plasma membrane to the cortical actin cytoskeleton [20].

### A significant number of proteins are increased or diminished in mutant strains

We next performed proteome analyses and identified from a total of 52,106 detected peptides with a FDR ≤ 0.01 49,400 (94.81 %) unique peptides (Table S4). Based on these unique peptides a total of 6,101 protein groups with a FDR ≤ 0.01 were identified of which 4,959 contained at least two unique peptides (Table S5). Proteome coverage was highly reproducible across the three biological replicates as more than 72 % of all identified proteins were detected in all three biological replicates and more than 13 % in two biological replicates. Proteins that were identified in only one biological replicate were excluded from downstream analysis. 5,224 of the identified proteins were found in all four strains.

**Fig. 2:**
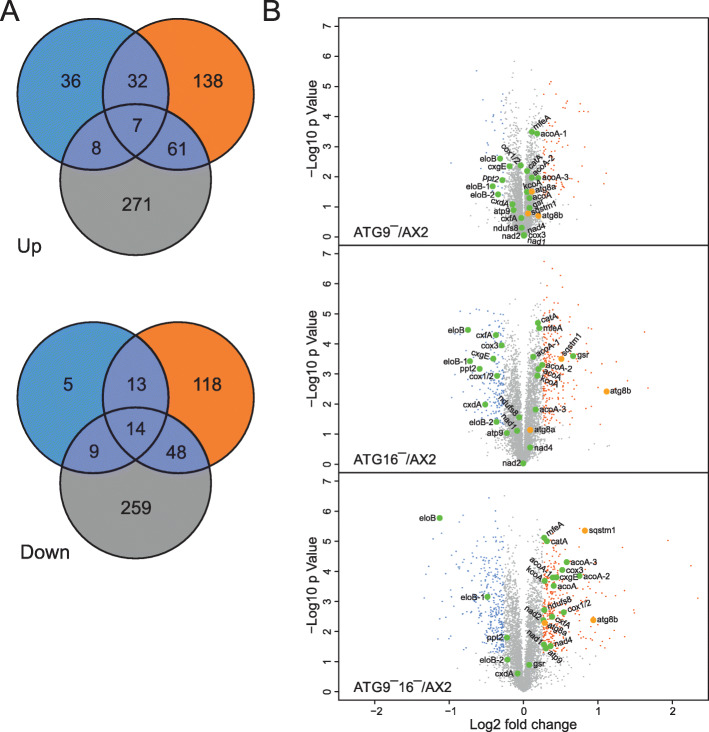
Differentially regulated proteins (DEPs). (**A**) Venn diagrams of identified DEPs in ATG9‾ (blue), ATG16‾ (orange), and ATG9‾/16‾ (grey) cells in comparison to AX2 wild-type. Top, up-regulated and bottom, down-regulated DEPs. (**B**) Presentation of DEPs in a volcano plot for each strain comparison. DEPs with Log2 fold change ≥ 0.262 and p ≤ 0.05 are labelled red, genes with Log2 fold change ≤ − 0.263 and p ≤ 0.05 are labelled blue. The autophagy proteins, ATG8a, ATG8b and Sqstm1, are highlighted by bigger orange-filled circles and DEPs from Table 4, i.e. proteins involved in lipid metabolism, oxidative phosphorylation and ROS clearance, by bigger green-filled circles and labelled with their Demerec names (see Table 4, Gene Product). The plot was created using the R environment (v. 2.15.0)

For the identification of DEPs we used an absolute threshold for FC ≥ 1.2 with a *p*-value ≤ 0.05 and found in comparison to AX2 wild-type cells 124 (83 up and 41 down) DEPs in ATG9‾ cells, 431 (238 up and 193 down) in ATG16‾ cells, and 677 (347 up and 330 down) in ATG9‾/16‾ cells (Table S6). Hierarchical clustering of the DEPs from the three biological replicates of ATG9‾, ATG16‾, and ATG9‾/16‾ cells in comparison to AX2 confirmed the high reproducibility of differential regulation across the biological replicates (Figure S1). The overall number of DEPs was lowest in ATG9‾, intermediate in ATG16‾ cells and highest in ATG9‾/16‾ cells. This result indicates a more severe cellular disbalance in ATG16‾ cells in comparison to ATG9‾ cells and a further escalation in the double mutant. Venn diagrams revealed that ATG9‾ and ATG16‾ strains shared 39 up- and 27 down-regulated proteins and ATG16‾ and ATG9‾/16‾ cells 68 up- and 62 down-regulated proteins, while the ATG9‾ and ATG9‾/16‾ cells only had 15 up- and 23 down-regulated proteins in common. (Fig. 2 A). We generated a heat map using the mean values of the DEPs from ATG9‾, ATG16‾ and ATG9‾/16‾ cells and confirmed their variation (Figure S2). The seven proteins, which were up- and the fourteen proteins, which were down-regulated in all three strains were of particular interest (Table S7). Of these 21 common DEPs, protein information was only available for 13 (four up- and nine down-regulated) proteins. Remarkably, three of the nine common down- and one of the four up-regulated proteins are involved in metabolic processes. Of the down-regulated proteins, thymidylate synthase (thyA) is involved in the *de novo* biosynthesis of pyrimidine deoxyribonucleotides, elongase B (eloB) in the elongation of fatty acids and ATP citrate synthase (acly) in the generation of citrate for the citrate cycle. Thus, nucleotide, lipid and carbohydrate metabolism might be affected in mutant strains. On the other hand the peroxisomal ATP citrate synthase homolog (cshA) was up-regulated in all three strains (Table S7).

Next we analysed all detected proteins of each strain comparison by volcano-plots (Fig. 2B). Proteins with a fold change ≥ 1.2 or ≤ 0.83 and p ≤ 0.05 are highlighted by red and blue points, respectively. The plots nicely illustrate the increasing number of DEPs from from ATG9‾, ATG16‾ to ATG9‾/16‾ cells in comparison to AX2 wild-type. GO annotation and manual inspection (see below) revealed specific DEPs involved in lipid metabolism, oxidative phosphorylation and ROS clearance. These are compiled in Table 4 and highlighted by big green circles (Fig. 2B). Furthermore, the increased autophagy proteins, ATG8a in ATG9‾/16‾, ATG8b in ATG16‾ and ATG9‾/16‾ cells are highlighted by big orange circles (Fig. 2B). Deletion of the core autophagy proteins ATG9 and/or ATG16 should impair autophagy flux, which may cause an increase of the autophagy substrate Sqstm1. Indeed, the protein level of Sqstm1 was increased in ATG16‾ and ATG9‾/16‾ but not in ATG9‾ cells (Fig. 2B; orange circles).

We also looked for proteins that were oppositely regulated in mutant strains. We found that 17 DEPs (14 up and 3 down) in ATG9‾ and 59 DEPs (35 up and 24 down) in ATG16‾ cells were oppositely regulated in ATG9‾/16‾ cells (Table S8). Interestingly, 10 of these DEPs (7 up and 3 down) were similarly regulated in the single mutants while they were oppositely regulated in the double mutant (Table S8). Only for 4 of these DEPs, i.e. lmcB, agnB, and alrE (all increased) and p17 (decreased), protein information was available. The cellular functions of *lmcB*, a vegetative specific gene repressed at the onset of development, of *agnB*, encoding an argonaut-like protein, and of *p17* or *sctA*, which encodes a secreted protein, are unknown. The differential expression and/or degradation of the latter suggests imbalances in secretion. *alrE* encodes an aldo-keto reductase and is involved in the detoxification of lipid peroxidation by-products that are produced by oxidative stress [21]. Furthermore, 2 proteins encoding dscA-1 and DDB_G0279397, were similarly dis-regulated in ATG9‾ and ATG9‾/16‾ cells, but oppositely regulated in ATG16‾ cells (Table S8).

### Parallel reaction monitoring (PRM) largely confirms Tandem Mass Tag (TMT) results

We selected six DEPs from all three comparisons for PRM to cross-check the TMT-based proteomics data. The peptide sequences used for PRM are listed in Table S9. For 4 of the 6 DEPs the PRM results showed similar trends for all three comparisons, for the other 2 DEPs there was partial agreement. In summary, this result largely supports the reliability of the TMT data (Table 2).

**Table 2:**
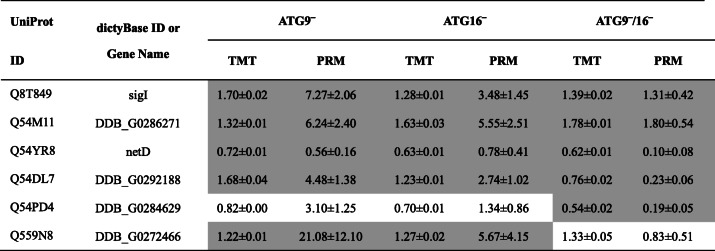
Comparison of quantification results between TMT and PRM

Grey background indicates the quantification results that showed similar trends between TMT and PRM. sig, srfA-induced gene; net, nuclear envelope transmembrane. The mean ratio of the fold change of mutant to AX2 and the standard error is displayed. Fold change and standard error are rounded at two places after the decimal point

### GO annotation reveals enrichment of proteins in distinct functional categories

To identify statistically significant enriched GO terms in our gene lists we used the program Panther [19]. A selection of enriched biological process, molecular function, and cellular component GO terms in the ATG9‾, ATG16‾, and ATG9‾/16‾ strains are listed in Table 3 and the full list of all enriched GO terms is available as supplementary information (Table S10). For the 83 up-regulated proteins of the ATG9‾ strain Panther did not report any GO term enrichment and for the 238 up-regulated proteins of the ATG16‾ strain the only enriched GO term was “phagocytic vesicle” in the cellular component ontology. 20 of the up-regulated proteins were placed by Panther in this category resulting in a 3-fold enrichment. In contrast, a number of enriched GO terms were reported for the 347 up-regulated proteins of the ATG9‾/16‾ cells. In the molecular function ontology, the three strongest enriched categories were acyl-CoA oxidase activity (15-fold), acetyl-CoA L-glutamate acetyltransferase activity (34-fold) and G-protein-β/γ-subunit complex binding (15-fold). This was reflected in the biological process ontology by fatty acid oxidation (9-fold), arginine biosynthetic process (22-fold), and adenylate cyclase-modulating G protein-coupled receptor signaling pathway (8-fold). It is worth mentioning that in the biological process ontology proteins involved in autophagy, actin cytoskeleton organization and regulation of phagocytosis were also enriched. In the cellular component ontology, this is mirrored by autophagosome, actin cytoskeleton, phagocytic cup and heterotrimeric G-protein complex. In addition, proteins of the GO terms peroxisome and mitochondrial inner membrane were enriched. The reporting of the latter category is caused by the up-regulation of 15 proteins of which 12 are classified as subunits of the mitochondrial respiratory chain (Table 3, see also Table 4).

**Table 3:**
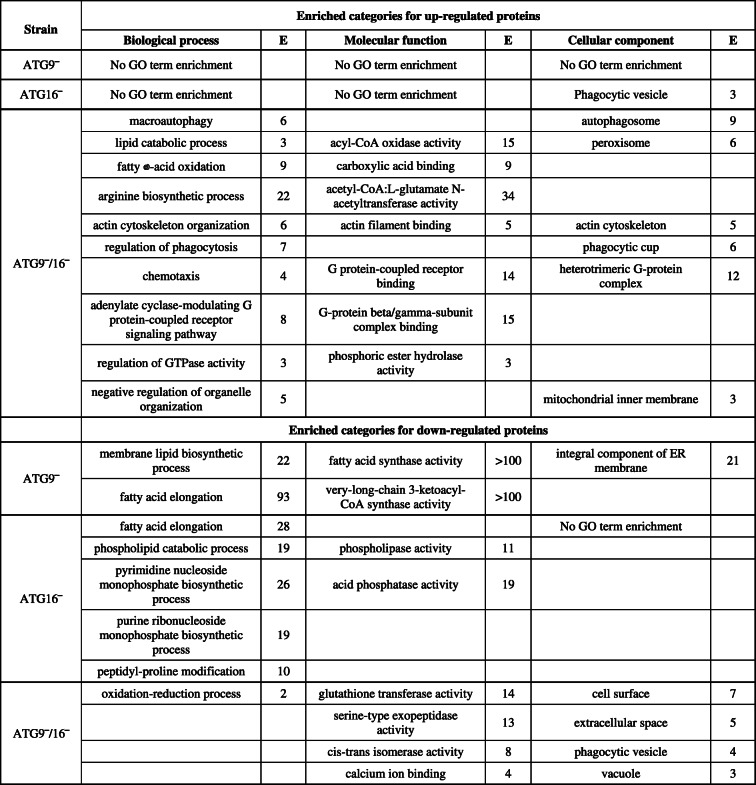
GO term enrichment analysis of DEPs in ATG9‾, ATG16‾ and ATG9‾/16‾

*E* enrichment (rounded integer), *ER* endoplasmic reticulum

**Table 4:**
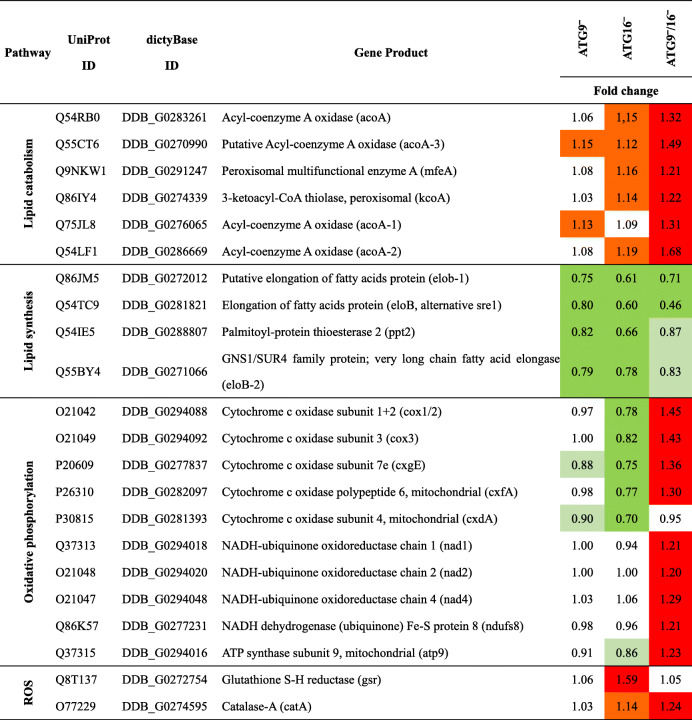
Specific DEPs involved in lipid metabolism, oxidative phosphorylation and ROS clearance (ROS)

Green, diminished; light green, slightly diminished (≤ 0.9 and ≥ 0.83); red, increased; orange, slightly increased (≥ 1.1 and ≤ 1.2). Fold change is rounded at two places after the decimal point

For the 41 down-regulated DEPs of the ATG9‾ strain strong enrichments for proteins involved in membrane lipid biosynthesis (22-fold) and fatty acid elongation (93-fold) in the biological process ontology were reported. Proteins with the corresponding enzymatic activities were more than 100-fold enriched in the molecular function ontology and proteins of the endoplasmic reticulum membrane were 21-fold enriched (Table 3). Three of the four proteins in this category are involved in fatty acid elongation. Interestingly, proteins required for fatty acid elongation were also enriched in the 193 DEPs of the ATG16‾ strain. Here, analysis of the biological process ontology also revealed strong enrichments of proteins involved in phospholipid catabolism, nucleoside monophosphate biosynthesis and peptidyl-proline modification. Proteins with phospholipase activity and with acid phosphatase activity were enriched in the molecular function ontology. No GO term enrichment was reported for the cellular component ontology. Despite the large number of 330 DEPs of the ATG9‾/16‾ strain, only a few enriched GO terms were reported. These were oxidation-reduction process in the biological process ontology, glutathione transferase activity, serine-type exopeptidase activity, cis-trans isomerase activity and calcium binding in the molecular function ontology, and cell surface, extracellular space, phagocytic vesicle and vacuole in the cellular component ontology (Table 3). In summary, we obtained mainly enrichment of GO terms for the ATG9‾/16‾ strain for the up-regulated DEPs and for the ATG9‾ and ATG16‾ strains for the down-regulated DEPs.

### Lipid metabolism and oxidative phosphorylation appear disturbed in mutant strains

GO term enrichment analysis showed that among the down-regulated proteins in the ATG9‾ and ATG16‾ strains, proteins involved in fatty acid elongation were over-represented, indicating that lipid synthesis was impaired in these strains (Table 3). In addition, we found that the cytochrome c oxidase (COX) subunits Cox1/2, Cox3, CxdA, CxfA, and CxgE were more or less unchanged in the ATG9‾ strain, all diminished in the ATG16‾ strain, but, with exception of CxdA, all were increased in the ATG9‾/16‾ strain (Table 4; Fig. 2B). In this strain several subunits of the NADH-ubiquinone oxidoreductase as well as the NADH dehydrogenase Fe-S protein 8 and the ATP synthase subunit 9 were also increased (Table 4; Fig. 2B). These are all proteins involved in oxidative phosphorylation indicating an up-regulation of mitochondrial respiration in the ATG9‾/16‾ strain. Mitochondrial respiration is the primary cellular source of reactive oxygen species (ROS) which need to be detoxified by anti-oxidant enzymes. Interestingly, we found that two enzymes involved in the removal of ROS downstream of the superoxide dismutase (SOD) were increased in our mutants, catalase A in the ATG9‾/16‾ and glutathione reductase in the ATG16‾ strain. Table 4 provides an overview of the differential expression of DEPs in selected enriched categories of the GO analysis in ATG9‾, ATG16‾ and ATG9‾/16‾ strains.

### Comparison of RNA_seq_ and proteomics data

**Fig. 3:**
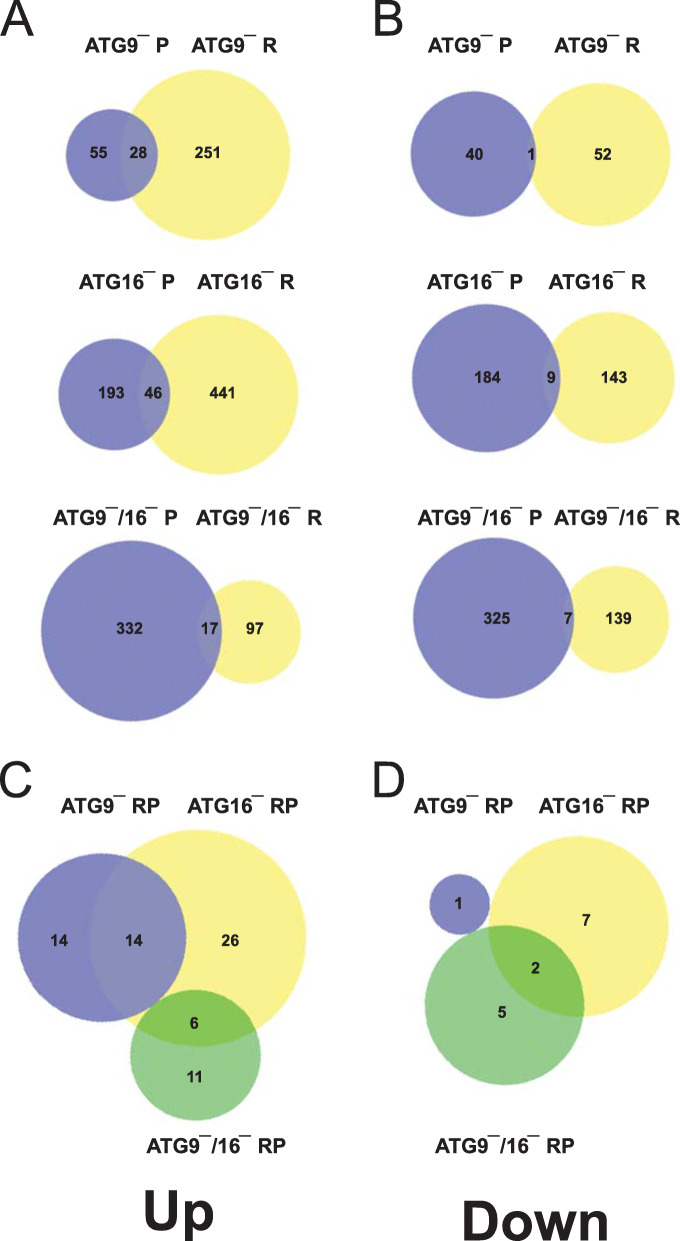
Proportional Venn diagrams of differentially regulated genes and proteins of ATG9‾, ATG16‾, and ATG9‾/16‾ cells versus AX2. (**A**) Up-regulated genes and proteins. (**B**) Down-regulated genes and proteins. (**C, D**) Presence of DEGs and DEPs of one strain, as identified by RNAseq and proteome analysis, in the other two strains. R, RNAseq; P, proteome analysis

We performed transcriptomic and proteomic analyses with ATG9‾, ATG16‾ and ATG9‾/16‾ strains. Both genome scale analyses delivered large and significant changes on the RNA and protein level in mutant strains in comparison to AX2 wild-type cells. We next addressed the question whether the differential regulation of RNAs was reflected on the protein level and *vice versa*. For up-regulated cellular entities we found that 28 (10 %) of the reported RNAs from ATG9‾ cells were also increased on the protein level (28 or 34 % of 83 proteins in total). Similarly, 46 (9.4 %) of the reported RNAs from ATG16‾ cells were also increased on the protein level (46 or 19 % of 239 proteins in total). For the ATG9‾/16‾ strain we found that 17 (14.9 %) of the reported RNAs were also increased on the protein level (17 or 4.9 % of 349 proteins in total) (Fig. 3 A, Table S11). We next compared these common entities among all three strains and found that of the 46 DEGs and DEPs of the ATG16‾ strain, 14 were also present in the ATG9‾ strain and 6 in the ATG9‾/16‾ strain (Table S11). Of these 20 entities, there was information for 11 proteins. However, for most of them the cellular function is so far unknown. Of the common up-regulated DEPs in the ATG9‾ and ATG16‾ strains hsp69 (heat shock protein 69) may function in the removal of protein aggregates that are caused by autophagy impairment and trafH (TNF receptor-associated factor H) may play a role in the response to pathogens as its mammalian homologues play pivotal roles in mediating inflammatory responses [22]. Of the common up-regulated DEPs in the ATG16‾ and ATG9‾/16‾ strains argS1 (arginine-tRNA ligase) and cinC (elongation factor 2) are involved in protein synthesis. This could mirror deranged protein synthesis in ATG16‾ and ATG9‾/16‾ cells. There was no up- or down-regulated gene and gene product that was common for all three strains (Fig. 3 C, Table S11). For down-regulated cellular entities we found that only 1 (1.9 %) of the reported RNAs from ATG9‾ cells and 9 (5.9 %) from ATG16‾ cells were also diminished on the protein level (2.4 % of 41 and 4.7 % of 193 proteins, respectively). For the ATG9‾/16‾ strain we found that 7 (4.8 %) of the reported RNAs were also diminished on the protein level (2.1 % of 332 total proteins) (Fig. 3B, Table S11). We next compared these common entities among all three strains and found that of the 9 differentially regulated genes and proteins of the ATG16‾ strain, only 2 were also present in the ATG9‾/16‾ strain (Fig. 3D, Table S11). One of the 2 entities was ATG16 and was down-regulated as expected. The other one was a B_lectin domain-containing protein with unknown function. In summary and quite unexpected we found that (i) only a small proportion of the transcriptional changes were reflected in corresponding changes on the protein level, and, vice versa, (ii) most proteome changes were not caused by transcriptional changes. Thus, most changes on the protein level in our mutant strains seem to be due to changes in protein translation and/or degradation. This could be due to the fact, that autophagy is the major lysosomal route for the turnover of cytoplasmic components, including damaged organelles and long-lived proteins and protein machineries [23].

## Discussion

We performed RNA_seq_ and TMT proteomics to identify DEGs and DEPs in ATG9‾, ATG16‾ and ATG9‾/16‾ strains in comparison to AX2 wild-type cells. RNA_seq_ delivers global transcriptional changes and TMT coupled with LC-MS/MS can precisely and simultaneously compare multiple samples for protein and peptide quantification [24, 25]. Precise quantitative PRM analysis was used to confirm the validity of a subset of the TMT results (Table 2 and S9) [26]. Furthermore, hierarchical clustering of DEPs from mutant cells in comparison to AX2 displayed high reproducibility across three biological replicates (Figure S1).

Overall, our results showed massive dysregulation of genes and proteins in mutant strains. RNA_seq_ analysis revealed 332 (279 up and 53 down), 639 (487 up and 152 down) and 260 (114 up and 146 down) DEGs in ATG9‾, ATG16‾ and ATG9‾/16‾ strains, respectively (Fig. 1, Table S1). TMT data revealed 124 (83 up and 41 down), 431 (238 up and 193 down) and 677 (347 up and 330 down) DEPs in ATG9‾, ATG16‾ and ATG9‾/16‾ strains, respectively (Fig. 2, Table S6). With 639 DEGs most transcriptional changes were occurring in ATG16‾ cells, while approximately half as many were seen in ATG9‾ and less than half as many in ATG9‾/16‾ cells (Table S2). This suggests that part of the DEGs in ATG9‾ and ATG16‾ cells are oppositely regulated, resulting in non-regulation or regulation below the threshold fold change in the double mutant. In contrast, we detected the lowest number of DEPs in ATG9‾, an intermediate number in ATG16‾ and the highest number in ATG9‾/16‾ cells, indicating an increasing disturbance of protein homeostasis from ATG9‾ to ATG16‾ to ATG9‾/16‾ cells. We were of course very interested in changes of autophagy components. On the transcriptional level we found that e.g. *atg1, atg8a, atg8b, atg18, atg10*, and *sqstm1* were at least 1.5-fold differentially regulated in *atg16* knock-out cells (Table 1). However, with exception of *atg8b and sqstm1* these changes were not reflected on the protein level (Fig. 2B, Table S6). Overall, we saw for only 71 (11.6 %) of the 610 up-regulated DEGs of the ATG9‾, ATG16‾ and ATG9‾/16‾ cells also an increase on the protein level. The situation was even worse for the down-regulated DEGs. There, only 15 (5.7 %) of the 261 DEGs were also decreased on the protein level (Figs. 1 and 3, Table S11). Thus, only a minority of the transcriptional changes were reflected on the protein level and *vice versa*. Weak correlations between mRNA and protein levels in large scale genomic analyses have also been noted in other studies [27]. It appears that regulatory processes affecting protein translation and/or degradation play a more dominant role which may be responsible for the weak correlation [28]. Autophagy is the major lysosomal route for degradation of damaged organelles and long-lived proteins and plays a pivotal role in protein homeostasis. In addition, we previously found, that the proteasomal activity of ATG9‾, ATG16‾, and ATG9‾/16‾ cells was strongly decreased which probably exacerbates the protein disbalance [15, 17]. Therefore, we speculate that most changes on the protein level in our mutant strains were not caused by transcriptional changes, but are likely due to changes in protein homeostasis. In the following part we will therefore discuss only the changes we detected on the protein level in the mutant strains.

Our further analysis was, however, hampered by the fact that most of the DEPs have so far no assigned cellular function. Approximately 34 % of all DEPs are designated “uncharacterized” and many more are without clear function (Table S6). Furthermore, only a small number of DEPs, 7 increased and 14 decreased, were common in the three mutant strains (Fig. 2 A and S2, Table S7). These common DEPs pointed to a disturbed metabolism in the autophagy mutants. We next carried out GO analysis of the DEPs from ATG9‾, ATG16‾ and ATG9‾/16‾ strains. For ATG9‾ and ATG16‾ cells we found for the down-regulated DEPs in the biological process and molecular function ontologies an enrichment of proteins involved in membrane lipid synthesis and fatty acid elongation (Table 3 and S10). Further analysis showed that four proteins (putative elongation of fatty acids protein, very long chain fatty acid elongase, palmitoyl-protein thioesterase 2 and elongation of fatty acids protein), which are involved in lipid synthesis, were diminished in these strains. Although no enrichment in this category was reported in the ATG9‾/16‾ strain (probably due to the high number of DEPs in this strain) we found that these proteins were also diminished in this strain (Table 4; Fig. 2B). Furthermore, we found that six proteins involved in lipid catabolism were slightly increased in ATG9‾ and ATG16‾ cells and strongly increased in the ATG9‾/16‾ strain (Table 2; Fig. 2B). These results suggest that the fatty acid metabolism is impaired in mutant strains. There is a clear connection between autophagy and lipid metabolism. It was reported that autophagy is required for liquid droplet breakdown and pharmacological inhibition of autophagy significantly increased hepatocyte triacylglycerol content [29]. In contrast, our results indicate that lipid content might be decreased in our autophagy mutants (Table 4; Fig. 2B). In a combined proteomic and genetic analysis the lipid desaturase Desat1 was found to be essential in starvation-induced autophagy in *Drosophila melanogaster* [30]. Furthermore, SCD1, its orthologue in mammalian cells, is important for the translocation of ULK1/ATG1 to the sites of autophagosome formation [31], while its yeast orthologue Ole1 is required for delivery of ATG9 to the autophagosome [32]. Thus, not only autophagy influences lipid metabolism, but there appears to be a regulatory cross-talk between these two essential cellular processes. A possible scenario is, that the dramatically reduced recycling of cellular building blocks in our autophagy mutants results in a shortage of energy. This would then trigger a decrease in lipid synthesis and an increase in lipid catabolism. We will analyze lipid content and lipid metabolism in our knock-out mutants in comparison to AX2 wild-type cells in future experiments.

Recently, it has been found that overexpression of the COX7A1 subunit of COX suppressed the autophagic flux and resulted in the accumulation of autophagosomes in the human non-small cell lung cancer cell line NCI-H838 [33]. Our TMT analysis showed that six COX subunits were decreased in the ATG16‾ strain (Table 4). COX is the primary site of cellular oxygen consumption and is essential for the generation of ATP [34]. A decrease in COX likely impairs the generation of ATP and may lead to the observed reduction in cell proliferation and viability during starvation in this mutant [17]. Furthermore, COX is the terminal oxidase of cell respiration and its deficiency causes an increase in mitochondrial reactive oxygen species (ROS) [35, 36]. This could also be the case in the ATG16‾ strain as the glutathione reductase (gsr) was strongly and the catalase-A (catA) slightly increased (Table 4; Fig. 2B). The latter was also increased in the ATG9‾/16‾ strain. The activity of the anti-oxidant enzyme catA, which converts hydrogen peroxide into water, is often used as an indicator for the level of ROS in cells [35–37]. In further analyses we will quantifiy ROS levels in wild-type and mutant strains and also determine the effect of autophagy inhibitors on ROS levels. This should reveal the function of autophagy in general and of ATG9 and ATG16 in particular in the elimination of ROS in *Dictyostelium*. In contrast to the ATG16‾ strain, five of these six COX subunits were increased in the ATG9‾/16‾ strain (Table 4; Fig. 2B). In addition, four subunits of the NADH dehydrogenase and subunit 9 of the ATP synthase, all involved in oxidative phosphorylation, were also increased (Table 2). This suggests an up-regulation of the respiratory chain in this strain, which fits to the increase of enzymes involved in lipid catabolism. These potential metabolic changes could result in an increased ATP production and may explain the significantly less severe growth defect in this strain in comparison to ATG16‾ cells [17]. We currently do not have an explanation for the opposite regulation of the six COX subunits in ATG16‾ and ATG9‾/16‾ cells. Further analyses of the function of the mitochondria in these mutants need to be addressed in future studies.

Deletion of either one of the two core autophagy-related proteins, ATG9 and ATG16, in *D. discoideum* resulted in pleiotropic cellular phenotypes and severely impaired autophagy [16, 17]. Our proteomics results indicate that in part a disturbance of lipid metabolism in all three mutant strains and of the respiratory chain in the ATG16‾ and ATG9‾/16‾ strains may be responsible for these phenotypes.

## Conclusions

In the current study, we used for the first time a combined approach of RNA_seq_ and TMT-based quantitative proteomics to identify distinct changes on the RNA and protein level in ATG9‾, ATG16‾ and ATG9‾/16‾ cells. RNA_seq_ results revealed that 332 (279 up and 53 down), 639 (487 up and 152 down) and 260 (114 up and 146 down) DEGs and TMT data showed 124 (83 up and 41 down), 431 (238 up and 193 down) and 677 (347 up and 330 down) DEPs in ATG9‾, ATG16‾ and ATG9‾/16‾ strains, respectively. Comparison of DEGs and DEPs showed that only a small proportion of the transcriptional changes were reflected in corresponding changes on the protein level. This suggests that most proteome changes were due to changes in protein translation and/or degradation and were not caused by transcriptional changes. GO analysis of DEPs revealed that lipid metabolism is likely disturbed in mutant strains. Furthermore, several enzymes of oxidative phosphorylation were found to be oppositely regulated in ATG16‾ and ATG9‾/16‾ cells. Despite this, both mutants appear to have an increase in ROS, as the anti-oxidant enzymes were increased in these strains. This study adds to our knowledge of cellular disbalances caused by autophagy dysfunction.

## Methods

### *Dictyostelium* strains and cell culture

*D. discoideum* AX2 was used as wild-type strain. The generation and phenotypes of ATG9‾, ATG16‾ and ATG9‾/16‾ strains are described elsewhere [16, 17]. AX2 and mutant cells were grown at 21 °C in AX2 medium (for 1 L: 14.3 g bacteriological peptone, 7.15 g yeast extract, 18 g maltose, 0.62 g Na_2_HPO_4_ × 2H_2_O, 0.49 g KH_2_PO_4_, pH 6.7) with shaking at 150 rpm in Erlenmeyer flasks. Log phase cells with a cell titer of 2–4 × 10^6^ cells/ml were harvested and washed twice with Soerensen buffer (2.0 mM Na_2_HPO_4_, 14.6 mM KH_2_PO_4_, pH 6.0) [17]. Cell pellets (2 × 10^7^ cells) were frozen in liquid nitrogen and stored at -80 °C until use for proteome analyses.

### RNA_seq_ analysis

Isolation and quality control of total RNA from vegetative *D. discoideum* cells and RNA_seq_ experiments were performed as described [38, 39]. Nine biological replicates of each strain were analyzed. Obtained sequences were filtered and preprocessed as described [40], aligned to the AX4 reference genome [41], and evaluated using QuickNGS version 1.26 [40]. The RNA_seq_ raw data, fragments per kilobase of transcript per million mapped reads (FPKM) values, and experimental information have been submitted to gene expression omnibus (GEO) (https://www.ncbi.nlm.nih.gov/geo/) and are available under the accession number GSE162070. RNA_seq_ analysis was done as described by using the DESeq2 package [39, 42]. Volcano plots and Venn diagrams were generated by using the statistical software environment R and Venny, respectively [39]. The Gene Ontology statistical overrepresentation tests were performed with PANTHER version 15.0 (released 2020-02-14) with the annotation sets “GO biological process”, “GO molecular function”, “GO cellular component”, and using Fisher’s Exact test with a FDR < 0.05 [43].

### Sample preparation, protein digestion, and TMT labeling

*Dictyostelium* cell pellets were lysed with SDT buffer (4 % SDS, 1 mM DTT, 100 mM Tris-HCl, pH 7.6) and then boiled for 15 min at 100 °C. The cell suspension was centrifuged at 14,000 x*g* for 40 min and the supernatant passed through 0.22 μm filters. The protein concentration of the filtered supernatant was determined using the BCA Protein Assay Kit (Bio-Rad, USA), and purity was determined by SDS-PAGE. 200 µg protein of each sample was digested with 4 µg trypsin (V5280, Promega) at 37 °C for 16 h. The resulting tryptic peptides were labeled using the TMT 6plex Reagent Kit (Thermo Fisher Scientific, USA) according to the manufacturer’s instructions. Three independent biological replicates of each strain were analyzed. TMT analysis was performed by Shanghai Applied Protein Technology Co., Ltd (Shanghai, China).

### Liquid chromatography-tandem mass spectrometry (LC-MS/MS) analysis

The labeled peptides were fractionated using the Pierce High pH Reversed-Phase Peptide Fractionation Kit (84,868, Thermo Scientific) according to the manufacturer’s instructions. Then equal amounts of the peptides were loaded onto a Thermo Fisher Scientific reverse phase trap column (Acclaim PepMap100, ø 100 μm x 2 cm, nanoViper C18) connected to the C18-reversed phase analytical Thermo Fisher Scientific EASY-Column™ (ø 75 μm × 10 cm, 3 μm resin, C18) in solvent A (99.9 % H_2_O, and 0.1 % formic acid), and eluted with solvent B (84 % acetonitrile, 15.9 % H_2_O, and 0.1 % formic acid) with a linear gradient (0–50 % solvent B for 100 min, 50–100 % solvent B for 8 min, and 100 % solvent B for 12 min at 300 nL/min) using the Easy nLC system. MS/MS was carried out with a Q-Exactive mass spectrometer (Thermo Finnigan LLC, San Jose, CA, USA) in the positive ion mode and a data-dependent manner choosing the most abundant precursor ions with a full MS scan from 300 to 1,800 m/z and resolution of 70,000 at m/z of 200. Target value determination was based on automatic gain control and dynamic exclusion duration was 60 s. MS/MS scans were acquired at a resolution of 17,500 at m/z of 200. Normalized collision energy was 30 eV and the underfill ratio was set at 0.1 % [44].

### Proteomic data analysis

The data files produced by 15 fractions MS/MS were processed by Proteome Discoverer 1.4 and searched by Mascot 2.2 (Matrix Science, MA) against 12,746 *D. discoideum* protein-coding sequences deposited in the UniProt database (downloaded on January 16, 2020). The parameters for data analysis were the following: trypsin was used for cleavage with a maximum of two missed cleavages; oxidation (M) and TMT6 plex (Y) were chosen as the variable modifications; carbamidomethyl (C), TMT 6 plex (N-term), and TMT 6 plex (K) were chosen as the fixed modifications; peptide mass tolerance was set at ± 20 ppm; and the fragment mass tolerance was set at 0.1 Da. All reported data were based on a FDR ≤ 0.01 and each identified protein had at least one unique peptide. The protein ratios were calculated as the median of only the unique protein peptides. All the peptide ratios were normalized against the median protein ratios. The median protein ratio should be 1 after the normalization. To determine the DEPs between different strains, a fold change > 1.2 and < 0.83, and a *p*-value < 0.05 were considered to represent up- or down-regulation in two comparable groups, respectively. The mass spectrometry proteomics data have been deposited to the ProteomeXchange Consortium (http://www.proteomexchange.org/) via the PRIDE [45] partner repository with the dataset identifier PXD023730.

### Bioinformatics

Volcano plots and Venn diagrams were generated by using the statistical software environment R and BioVenn (http://www.biovenn.nl/), respectively [39, 46]. The Gene Ontology statistical overrepresentation tests were performed with PANTHER version 15.0 (released 2020-02-14) with the annotation sets “GO biological process”, “GO molecular function”, “GO cellular component”, and using Fisher’s Exact test with a FDR < 0.05 [43]. PANTHER was used to annotate Gene Ontology (GO) and a GO term with *p* < 0.05 after fisher’s exact test was considered significantly enriched [19].

### Parallel reaction monitoring (PRM) validation

The PRM method was applied to validate the accuracy of the TMT results and was done by Shanghai Applied Protein Technology Co., Ltd [47, 48]. Briefly, peptides were prepared according to the TMT protocol, and an AQUA stable isotope peptide was spiked in each sample as internal standard reference. Tryptic peptides were loaded on a home-made 75 μm*200 mm, 3 μm-C18 stage tip column for desalting prior to reversed-phase chromatography on an Easy nLC-1200 system (Thermo Fisher Scientific, CA, USA). Peptides were eluted by a 5 to 35 % acetonitrile gradient for 45 min followed by 5 % acetonitrile for 15 min. PRM analysis was performed on a Q Exactive Plus mass spectrometer (Thermo Fisher Scientific, CA, USA). A unique peptide with high strength and confidence for each target protein was used to optimize the collision energy charge state and retention time of the most significantly regulated peptides. The mass spectrometer was operated in positive ion mode and with the following parameters: The full MS1 scan was acquired with the resolution of 70,000 (at 200 m/z), automatic gain control (AGC) target values 3.0 × 10^− 6^, and 250 ms maximum ion injection times. Full MS scans were followed by 20 PRM scans at 35,000 resolution (at m/z 200) with AGC 3.0 × 10^− 6^ and 200 ms maximum injection times. The targeted peptides were isolated with a 2 thomson (Th) window. Peptides were selected for MS2 scans using HCD operating mode with a normalized collision energy setting of 27 %. The raw data were analyzed using Skyline 3.5.0 [49] where signal intensities for individual peptide sequences for each of the significantly altered proteins were quantified relative to each sample and normalized to standard reference.

## Supplementary Material

**Additional file 1.**

**Additional file 2.**

## Data Availability

The RNA_seq_ raw data, fragments per kilobase of transcript per million mapped reads (FPKM) values, and experimental information have been submitted to gene expression omnibus (GEO) (https://www.ncbi.nlm.nih.gov/geo/) and are available under the accession number GSE162070 (link: https://www.ncbi.nlm.nih.gov/geo/query/acc.cgi?acc=GSE162070). The mass spectrometry proteomics data have been deposited to the ProteomeXchange Consortium (http://www.proteomexchange.org/) with the dataset identifier PXD023730. All other datasets supporting the conclusions of this article are included within the article and its additional files “Supplementary information”.

## Abbreviations

AGC: automatic gain control
ATG: autophagy-related
COX: cytochrome c oxidase
DEG: differentially expressed genes
DEP: differentially expressed proteins
FC: fold change
FPKM: fragments per kilobase of transcript per million mapped reads
GEO: gene expression omnibus
GO: gene ontology
HCD: higher-energy c-trap dissociation
PE: phosphatidylethanolamine
PRM: parallel reaction monitoring
ROS: reactive oxygen species
SOD: superoxide dismutase
TGN: trans-Golgi network
Th: thomson
TMT: tandem mass tag
