# Correction to: RNA_seq_ and quantitative proteomic analysis of *Dictyostelium* knock-out cells lacking the core autophagy proteins ATG9 and/or ATG16

**DOI:** 10.1186/s12864-021-07863-0

**Published:** 2021-07-19

**Authors:** Qiuhong Xiong, Ning Song, Ping Li, Sarah Fischer, Roman Konertz, Prerana Wagle, Gernot Glöckner, Changxin Wu, Ludwig Eichinger

**Affiliations:** 1 Institutes of Biomedical Sciences, Key Laboratory of Chemical Biology and Molecular Engineering of Ministry of Education, Shanxi University, No. 92 Wucheng Road, Taiyuan, 030006 China; 2 Center for Biochemistry, Medical Faculty, University of Cologne, Joseph-Stelzmann-Str. 52, 50931 Cologne, Germany; 3 Cologne Excellence Cluster on Cellular Stress Responses in Aging Associated Diseases, University of Cologne, Joseph-Stelzmann-Str. 26, 50931 Cologne, Germany

**Correction to: BMC Genomics 22, 444 (2021)**

**https://doi.org/10.1186/s12864-021-07756-2**

Following publication of the original article [1], it was reported that there was an error in Table 3. The corrected Table 3 is included in this Correction article and the original article has been updated.

**Table 3:**
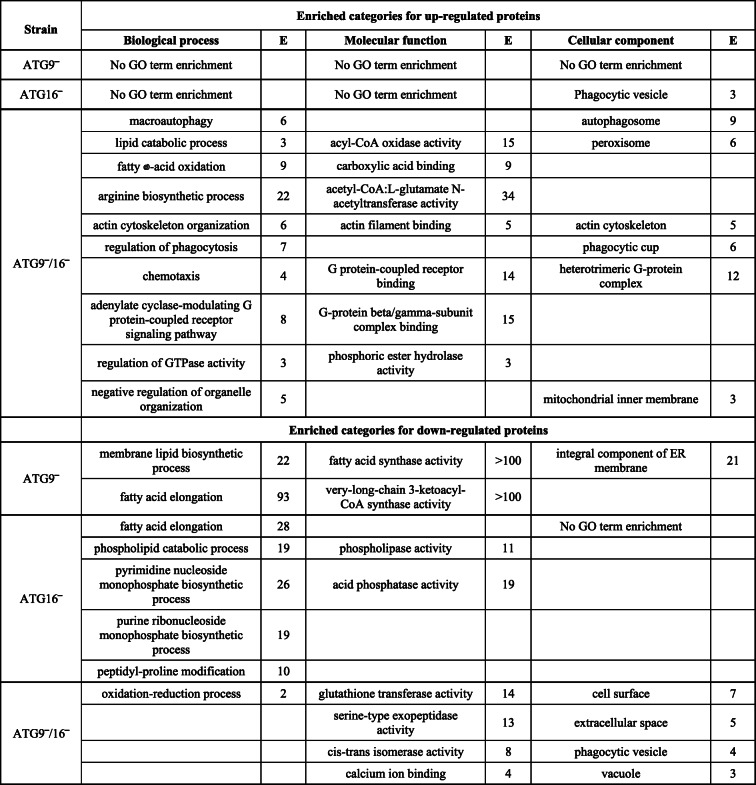
GO term enrichment analysis of DEPs in ATG9^−^, ATG16^−^ and ATG9^−^/16^−^

*E* enrichment (rounded integer), *ER* endoplasmic reticulum
