# Supplementary material for: RNAseq and quantitative proteomic analysis of Dictyostelium knock-out cells lacking the core autophagy proteins ATG9 and/or ATG16
Source: BMC Genomics. 2021 Jun 15;22:444. doi: 10.1186/s12864-021-07756-2 (PMC8204557; doi:10.1186/s12864-021-07756-2)
Supplement: Supplementary file 1 — Additional file 1. [file 12864_2021_7756_MOESM1_ESM.pdf]

Figure S1

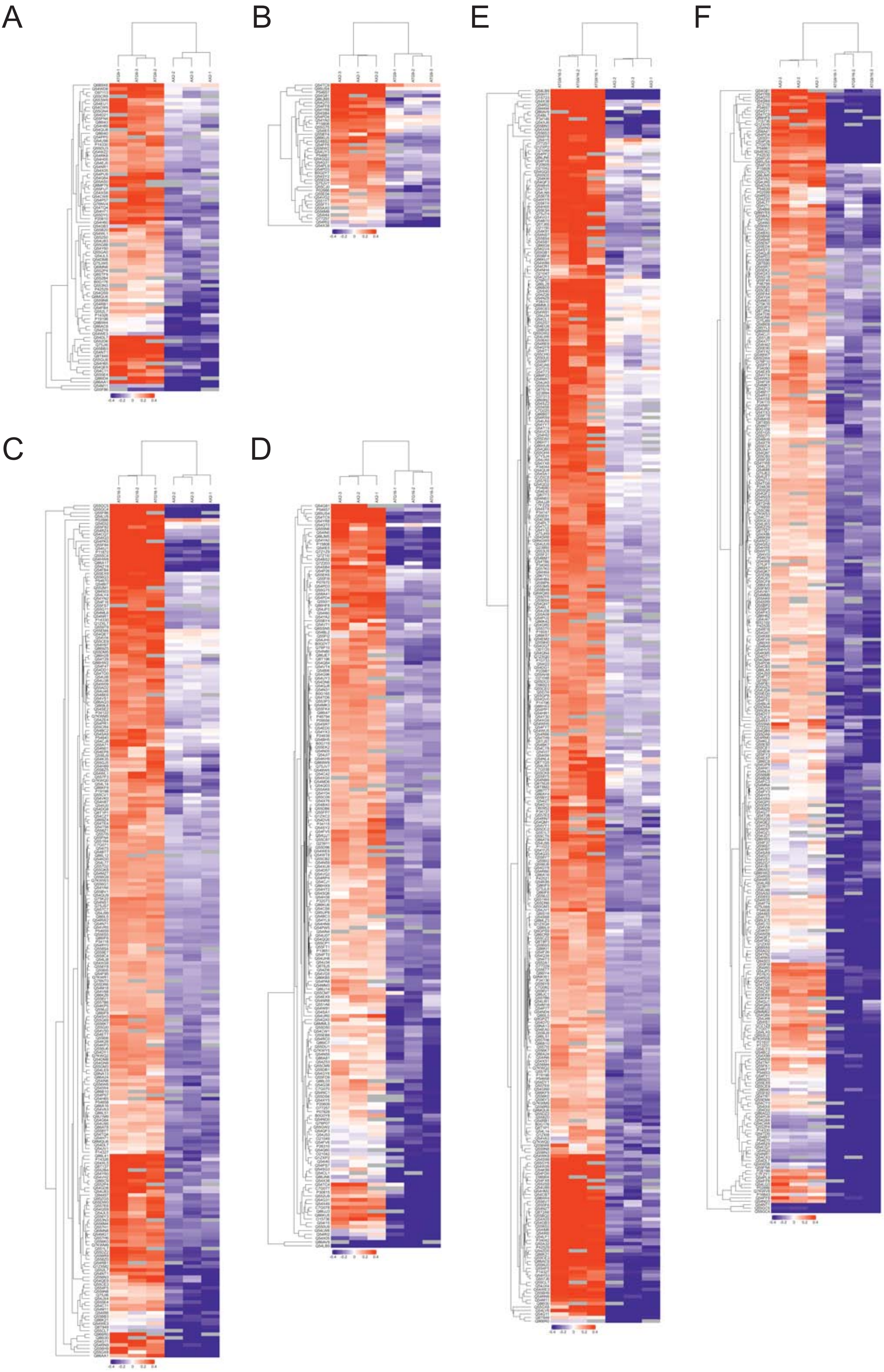

Figure S1: Hierarchical clustering analysis of DEPs of mutant strains versus AX2.

Figure S2

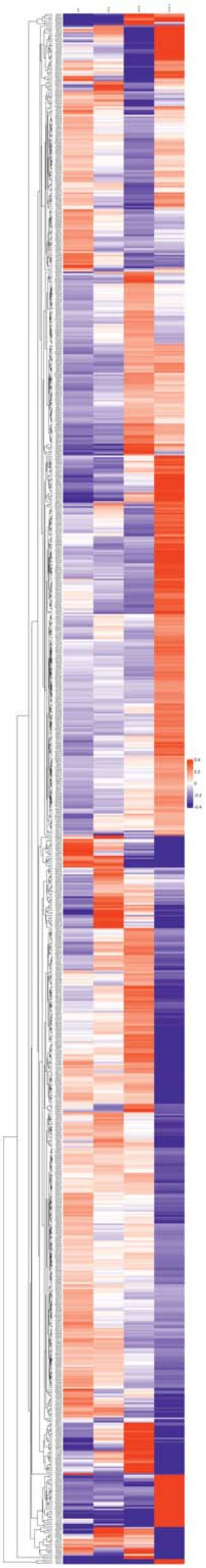

Figure S2: Heat map of DEPs of three mutant strains versus AX2.
