# Supplementary material for: RNAseq and quantitative proteomic analysis of Dictyostelium knock-out cells lacking the core autophagy proteins ATG9 and/or ATG16
Source: BMC Genomics. 2021 Jun 15;22:444. doi: 10.1186/s12864-021-07756-2 (PMC8204557; doi:10.1186/s12864-021-07756-2)
Supplement: Supplementary file 2 — Additional file 2. [file 12864_2021_7756_MOESM2_ESM.zip › Xiong et al Supplementary tables revision2.pdf]

Table S1. List of DEGs in ATG9¯, ATG16¯ and ATG9¯/16¯ cells in comparison to AX2. Cells were subjected to RNA_{Seq} analysis and fold changes (FC) and p-values of genes from mutant strains versus AX2 were determined. Nine biological replicates were analyzed. For each strain comparison only those genes with a p-value ≤ 0.05 and at least 2-fold differential regulation are depicted. #, number; N/A, not available.

Up-regulated genes in ATG9¯ versus AX2 cells

#	DDB_G ID	UniProt ID	GeneName	GeneProduct	FC	p-value
1	DDB_G0288573	Q54IR6	DDB_G0288573	unknown	12.87	0.000
2	DDB_G0276097	Q75JI6	DDB_G0276097	putative transmembrane protein	11.76	0.000
3	DDB_G0293202	Q54C11	trafH	TNF receptor-associated factor H	10.27	0.000
4	DDB_G0275487	Q86ID4	DDB_G0275487	unknown	9.79	0.000
5	DDB_G0269482	Q55DY0	DDB_G0269482	unknown	9.34	0.000
6	DDB_G0292188	Q54DL7	DDB_G0292188	unknown	9.08	0.000
7	DDB_G0290637	Q54FU4	DDB_G0290637	unknown	8.34	0.000
8	DDB_G0276219	Q552D6	DDB_G0276219	putative transmembrane protein	8.23	0.000
9	DDB_G0289681	Q54H65	DDB_G0289681	unknown	8.14	0.000
10	DDB_G0289787	Q54H60	DDB_G0289787	unknown	7.03	0.000
11	DDB_G0281691	Q54TL1	DDB_G0281691	unknown	7.01	0.000
12	DDB_G0289507	Q54HF0	act25	actin	6.92	0.000
13	DDB_G0292652	Q54CW8	DDB_G0292652	unknown	6.61	0.000
14	DDB_G0284121	Q54Q26	DDB_G0284121	transmembrane protein	6.61	0.000
15	DDB_G0268556	Q55FQ6	psiE	PA14 domain-containing protein	6.39	0.000
16	DDB_G0268874	Q55EI9	DDB_G0268874	unknown	6.29	0.000
17	DDB_G0271920	Q86I91	DDB_G0271920	unknown	6.06	0.000
18	DDB_G0268318	Q55GE7	DDB_G0268318	unknown	6.04	0.000
19	DDB_G0283913	Q54QE8	DDB_G0283913	heat shock protein Hsp20 domain-containing protein	5.93	0.000
20	DDB_G0275033	Q86I68	DDB_G0275033	unknown	5.71	0.000
21	DDB_G0288745	Q1ZXD1	DDB_G0288745	unknown	5.49	0.000
22	DDB_G0274291	Q86AA1	lyT2-4	putative T4-like lysozyme 2	5.47	0.000
23	DDB_G0272506	Q7KWU5	DDB_G0272506	unknown	5.29	0.000
24	DDB_G0284779	Q54P57	DDB_G0284779	unknown	5.15	0.000
25	DDB_G0290377	Q54G64	agnB	argonaut-like protein	5.15	0.000
26	DDB_G0280615	Q54V50	DDB_G0280615	unknown	5.13	0.000
27	DDB_G0282333	Q54SN7	DDB_G0282333	endonuclease/exonuclease/phosphatase domain-containing protein	5.06	0.000
28	DDB_G0290305	Q54G91	DDB_G0290305	unknown	4.98	0.000
29	DDB_G0274831	Q556F2	lyT1-4	putative T4-like lysozyme 1	4.95	0.000
30	DDB_G0289505	Q54HF1	act24	actin	4.93	0.000
31	DDB_G0289675	Q54H71	adprh	ADP-ribosylarginine hydrolase	4.91	0.000
32	DDB_G0280501	Q54V98	DDB_G0280501	unknown	4.83	0.000
33	DDB_G0290993	Q54F96	DDB_G0290993	unknown	4.72	0.000
34	DDB_G0281087	Q54UG8	gtaV	GATA zinc finger domain-containing protein 22	4.71	0.000
35	DDB_G0281817	Q54TF4	DDB_G0281817	unknown	4.61	0.000
36	DDB_G0283197	Q54RC8	DDB_G0283197	C2H2-type zinc finger-containing protein	4.46	0.000
37	DDB_G0278391	Q54Y65	DDB_G0278391	unknown	4.44	0.000

38	DDB_G0271140	Q8T6J0	abcA7	ABC transporter A family protein	4.32	0.000
39	DDB_G0285791	Q54MW2	DDB_G0285791	unknown	4.19	0.000
40	DDB_G0282293	Q6TMI9	rnpA	RNA recognition motif-containing protein RRM, putative RNA binding protein	4.16	0.000
41	DDB_G0276479	Q86HV8	ctnC	countin3	4.08	0.000
42	DDB_G0283979	Q54QA1	DDB_G0283979	unknown	4.03	0.000
43	DDB_G0283553	Q54QX5	DDB_G0283553	patatin family protein	4.03	0.000
44	DDB_G0289411	Q54HJ4	DDB_G0289411	unknown	3.98	0.000
45	DDB_G0288419	Q54IY9	DDB_G0288419	unknown	3.92	0.000
46	DDB_G0268208	Q55F86	DDB_G0268208	unknown	3.87	0.000
47	DDB_G0283911	Q54QE9	hsp69	heat shock protein 69	3.82	0.000
48	DDB_G0291646	Q54EB9	DDB_G0291646	unknown	3.82	0.000
49	DDB_G0290931	Q54FD5	trafL	TNF receptor-associated factor L	3.77	0.000
50	DDB_G0293274	Q54C10	DDB_G0293274	B-box zinc finger-containing protein/FNIP repeat-containing protein	3.75	0.000
51	DDB_G0286153	Q54M68	DDB_G0286153	unknown	3.74	0.000
52	DDB_G0277809	Q54Z64	cfaA	counting factor associated protein	3.70	0.000
53	DDB_G0289487	P07829	act3	actin	3.69	0.000
54	DDB_G0286911	Q54L47	DDB_G0286911	unknown	3.68	0.000
55	DDB_G0288623	Q966Q9	cbpH	calcium-binding protein	3.61	0.000
56	DDB_G0271138	Q55BC0	abcA8	ABC transporter A family protein	3.60	0.000
57	DDB_G0349499	Q558U0	DDB_G0349499	unknown	3.55	0.000
58	DDB_G0288571	Q54IR7	DDB_G0288571	unknown	3.54	0.000
59	DDB_G0279707	Q54WE3	iliP	DUF3430 family protein	3.52	0.000
60	DDB_G0286551	Q54LH9	gerE	germination protein GerE	3.46	0.000
61	DDB_G0288737	Q54IJ4	DDB_G0288737	unknown	3.46	0.000
62	DDB_G0268146	Q55FE9	DDB_G0268146	unknown	3.46	0.000
63	DDB_G0278177	Q54YM0	DDB_G0278177	unknown	3.45	0.000
64	DDB_G0268144	Q6B9X6	vwkA	alpha protein kinase VwkA	3.39	0.000
65	DDB_G0276793	Q7KWW7	DDB_G0276793	unknown	3.39	0.000
66	DDB_G0280919	Q54UN8	DDB_G0280919	unknown	3.38	0.000
67	DDB_G0272867	Q558Z0	argS1	arginyl-tRNA synthetase	3.38	0.000
68	DDB_G0289467	Q58A41	DD8-14	AAA ATPase domain-containing protein	3.34	0.000
69	DDB_G0280425	Q54VE1	DDB_G0280425	unknown	3.34	0.000
70	DDB_G0290959	Q54FB1	chtC	cheater C	3.33	0.000
71	DDB_G0288417	Q54IZ0	DDB_G0288417	unknown	3.30	0.000
72	DDB_G0272919	Q1ZXN2	DDB_G0272919	unknown	3.29	0.000
73	DDB_G0278011	Q1ZXJ6	racQ	Rho GTPase	3.29	0.000
74	DDB_G0272769	Q86L41	DDB_G0272769	unknown	3.25	0.000
75	DDB_G0268850	Q55EK5	DDB_G0268850	putative ATP binding protein	3.23	0.000
76	DDB_G0278647	Q54YE1	tps7	terpene synthase 7	3.16	0.000
77	DDB_G0278173	Q54YM2	DDB_G0278173	unknown	3.15	0.000
78	DDB_G0290381	Q54G62	DDB_G0290381	unknown	3.14	0.000
79	DDB_G0274637	Q554W2	DDB_G0274637	unknown	3.10	0.000
80	DDB_G0275171	Q86I15	DDB_G0275171	unknown	3.09	0.000
81	DDB_G0281753	Q54TH1	DDB_G0281753	unknown	3.06	0.000
82	DDB_G0272714	Q86II9	DDB_G0272714	unknown	3.04	0.000

83	DDB_G0277989	Q54YZ5	DDB_G0277989	putative protein kinase	3.03	0.000
84	DDB_G0285215	Q54NJ3	DDB_G0285215	unknown	2.99	0.000
85	DDB_G0293948	Q54B19	DDB_G0293948	unknown	2.99	0.000
86	DDB_G0276747	Q550Z2	DDB_G0276747	unknown	2.98	0.000
87	DDB_G0283595	Q54QX7	DDB_G0283595	patatin family protein	2.94	0.000
88	DDB_G0288399	Q54J01	DDB_G0288399	unknown	2.93	0.000
89	DDB_G0275161	Q86I43	DDB_G0275161	unknown	2.91	0.000
90	DDB_G0290379	Q54G63	DDB_G0290379	unknown	2.90	0.000
91	DDB_G0276351	Q8SSN4	DDB_G0276351	putative glutathione S-transferase	2.88	0.000
92	DDB_G0291796	Q54E55	araA	putative regulator of adhesion and motility 4	2.88	0.000
93	DDB_G0271916	P54681	rtoA	unknown	2.87	0.000
94	DDB_G0276361	Q86AC9	DDB_G0276361	unknown	2.87	0.000
95	DDB_G0285923	Q54MI4	DDB_G0285923	unknown	2.85	0.000
96	DDB_G0277389	Q75J87	DDB_G0277389	unknown	2.85	0.000
97	DDB_G0276705	Q550Z4	DDB_G0276705	unknown	2.84	0.000
98	DDB_G0267414	Q55G81	repD	transcription factor IIH component	2.84	0.000
99	DDB_G0289549	Q54HC9	DDB_G0289549	unknown	2.81	0.000
100	DDB_G0283429	Q54R22	DDB_G0283429	unknown	2.80	0.000
101	DDB_G0286907	P0CG77	ubqD	ubiquitin D	2.80	0.000
102	DDB_G0281345	Q54U30	DDB_G0281345	unknown	2.80	0.000
103	DDB_G0272829	Q86L54	DDB_G0272829	unknown	2.80	0.000
104	DDB_G0294575	Q1ZXJ5	DDB_G0294575	ankyrin repeat-containing protein	2.79	0.000
105	DDB_G0291592	Q54EB8	DDB_G0291592	unknown	2.78	0.000
106	DDB_G0272827	Q966R0	cbpI	EF-hand domain-containing protein	2.78	0.000
107	DDB_G0268802	Q55EP5	gpaJ	G-protein subunit alpha 10	2.76	0.000
108	DDB_G0269254	Q6TMJ3	sigJ	unknown	2.76	0.000
109	DDB_G0290215	Q54GE2	DDB_G0290215	unknown	2.73	0.000
110	DDB_G0268852	Q55EK4	DDB_G0268852	putative ATP binding protein	2.72	0.000
111	DDB_G0285289	Q54NF4	spoB	spore-specific protein B	2.72	0.000
112	DDB_G0273451	Q557Q0	DDB_G0273451	AAA+ ATPase, core domain-containing protein	2.71	0.000
113	DDB_G0267790	Q55G73	DDB_G0267790	unknown	2.69	0.000
114	DDB_G0289917	Q54GU5	DDB_G0289917	unknown	2.69	0.000
115	DDB_G0294515	N/A	dutA	structural RNA	2.69	0.000
116	DDB_G0279995	Q54VZ5	DDB_G0279995	putative glycoside hydrolase	2.69	0.000
117	DDB_G0272182	Q75JW5	DDB_G0272182	putative arginine deiminase	2.68	0.000
118	DDB_G0291714	Q8T9W4	abcB3	ABC transporter B family protein AbcB3	2.66	0.000
119	DDB_G0281607	Q54TR0	DDB_G0281607	unknown	2.63	0.000
120	DDB_G0289915	Q54GU6	DDB_G0289915	Phospholipid scramblase 3	2.61	0.000
121	DDB_G0274611	N/A	DDB_G0274611_ps	pseudogene	2.60	0.000
122	DDB_G0285165	Q54NL1	abcC9	ABC transporter C family protein	2.60	0.000
123	DDB_G0286129	Q54M93	DDB_G0286129	F-box/WD repeat-containing protein	2.59	0.000
124	DDB_G0282919	Q54RU5	DDB_G0282919_TE	Tdd-4	2.59	0.000
125	DDB_G0267612	Q55GM2	DDB_G0267612	unknown	2.58	0.000
126	DDB_G0278765	Q54XT1	DDB_G0278765	unknown	2.57	0.000

127	DDB_G0270212	Q58A40	DDB_G0270212	galactose-binding domain-containing protein	2.56	0.000
128	DDB_G0293794	Q54BA7	DDB_G0293794	unknown	2.55	0.000
129	DDB_G0267578	Q55GP1	DDB_G0267578	unknown	2.53	0.000
130	DDB_G0282737	Q54S05	DDB_G0282737	unknown	2.53	0.000
131	DDB_G0282715	Q54S16	DDB_G0282715	Neutral and basic amino acid transport protein rBAT	2.51	0.000
132	DDB_G0285615	Q54MX8	iliA	unknown	2.50	0.000
133	DDB_G0284619	Q54PE1	DDB_G0284619	unknown	2.50	0.000
134	DDB_G0274121	Q555Z5	abcA4	ABC transporter A family protein	2.49	0.000
135	DDB_G0268142	Q55FF1	DDB_G0268142	unknown	2.49	0.000
136	DDB_G0280703	Q54UZ9	DDB_G0280703	elongation factor 1beta-related protein	2.48	0.000
137	DDB_G0277711	Q54ZA1	DDB_G0277711_RTE	TRE5-B ORF2	2.48	0.000
138	DDB_G0278549	Q54XW7	gacB	RhoGAP domain-containing protein	2.47	0.000
139	DDB_G0276729	Q550X9	DDB_G0276729	unknown	2.47	0.000
140	DDB_G0279483	Q54WR4	pldB	phospholipase D1	2.47	0.000
141	DDB_G0288033	Q54JI3	DDB_G0288033	unknown	2.46	0.000
142	DDB_G0287097	Q54KU2	DDB_G0287097	FNIP repeat-containing protein	2.46	0.000
143	DDB_G0272072	Q86LA5	DDB_G0272072	unknown	2.45	0.000
144	DDB_G0284645	Q54PC5	DDB_G0284645	unknown	2.45	0.000
145	DDB_G0279307	Q54WZ3	vacC	vacuolin C, band 7 family protein	2.44	0.000
146	DDB_G0277379	P54643	pspD	spore coat protein SP87	2.42	0.000
147	DDB_G0280649	N/A	N/A	N/A	2.42	0.000
148	DDB_G0274613	Q554L2	DDB_G0274613	RING zinc finger-containing protein, putative protein serine/threonine kinase, CMGC group	2.42	0.000
149	DDB_G0292334	Q54DC6	DDB_G0292334	unknown	2.40	0.000
150	DDB_G0278667	Q54Y89	DDB_G0278667	unknown	2.40	0.000
151	DDB_G0281135	Q54UD9	DDB_G0281135	unknown	2.40	0.000
152	DDB_G0285685	Q54MS9	DDB_G0285685	unknown	2.40	0.000
153	DDB_G0291952	Q1ZXB2	DDB_G0291952	protease pro-fragment	2.39	0.000
154	DDB_G0280977	Q8ST87	abcC10	ABC transporter C family protein	2.39	0.000
155	DDB_G0278613	Q54YP0	DDB_G0278613	EGF-like domain-containing protein, matrilin-like protein	2.39	0.000
156	DDB_G0279899	Q54W64	DDB_G0279899	unknown	2.38	0.000
157	DDB_G0287841	Q54JR1	DDB_G0287841	unknown	2.38	0.000
158	DDB_G0282141	P13231	hatA	actin binding protein, hisactophilin I	2.38	0.000
159	DDB_G0288791	Q54IF5	hlcs1	biotin--[acetyl-CoA-carboxylase] ligase 1	2.37	0.000
160	DDB_G0282991	Q54RQ2	DDB_G0282991	unknown	2.36	0.000
161	DDB_G0285345	Q54ND0	DDB_G0285345	unknown	2.36	0.000
162	DDB_G0277791	Q54Z85	racO	Rho GTPase RacO	2.35	0.000
163	DDB_G0294338	Q54AM7	DDB_G0294338_RTE	DGLT-A	2.35	0.000
164	DDB_G0288519	Q54IU1	DDB_G0288519	unknown	2.35	0.000
165	DDB_G0270214	Q55C60	DD7-1	galactose-binding domain-containing protein	2.35	0.000
166	DDB_G0287581	Q54K61	DDB_G0287581	unknown	2.34	0.000
167	DDB_G0272773	Q86L36	DDB_G0272773	DUF3430 family protein	2.34	0.000
168	DDB_G0276731	Q550Y0	DDB_G0276731	Zinc finger protein 717	2.33	0.000
169	DDB_G0282567	Q54SA8	DDB_G0282567	unknown	2.33	0.000

170	DDB_G0281075	Q54UH6	DDB_G0281075	unknown	2.32	0.000
171	DDB_G0268446	Q55FE6	cbhA	cellobiohydrolase A	2.31	0.000
172	DDB_G0289641	Q54H89	DDB_G0289641	unknown	2.31	0.000
173	DDB_G0279119	Q54X91	DDB_G0279119	unknown	2.31	0.000
174	DDB_G0272999	Q558Y3	pkd2	polycystin-2	2.31	0.000
175	DDB_G0276905	Q86JA8	DDB_G0276905	unknown	2.31	0.000
176	DDB_G0286983	Q54L10	DDB_G0286983	unknown	2.30	0.000
177	DDB_G0289683	Q54H64	DDB_G0289683	unknown	2.30	0.000
178	DDB_G0272666	Q86JC1	DDB_G0272666	putative ankyrin repeat protein	2.30	0.000
179	DDB_G0276745	Q550Y9	DDB_G0276745	unknown	2.29	0.000
180	DDB_G0291758	Q54E82	DDB_G0291758	unknown	2.29	0.000
181	DDB_G0288713	Q54IJ5	DDB_G0288713	unknown	2.28	0.000
182	DDB_G0277793	Q54Z79	DDB_G0277793	unknown	2.28	0.000
183	DDB_G0274477	Q86HQ5	DDB_G0274477	unknown	2.27	0.000
184	DDB_G0282059	Q54T10	DDB_G0282059	unknown	2.26	0.000
185	DDB_G0285237	Q54NJ0	DDB_G0285237	unknown	2.25	0.000
186	DDB_G0270306	Q55BY9	DDB_G0270306	putative transcriptional regulator	2.25	0.000
187	DDB_G0291083	Q54F65	DDB_G0291083	unknown	2.25	0.000
188	DDB_G0290491	Q54G11	atg8b	autophagy protein 8b	2.24	0.000
189	DDB_G0273021	Q7KWN4	sigN5	SrfA-induced protein N5	2.24	0.001
190	DDB_G0270750	Q55C86	DDB_G0270750	Kelch repeat-containing protein	2.23	0.000
191	DDB_G0283833	Q54QH1	DDB_G0283833	unknown	2.23	0.000
192	DDB_G0270700	Q55CM2	DDB_G0270700	calcium-binding EGF domain-containing protein	2.22	0.000
193	DDB_G0268600	Q55F82	uduB	unknown	2.22	0.000
194	DDB_G0272280	Q559T9	DDB_G0272280	AhpC/TSA family protein	2.21	0.000
195	DDB_G0277783	Q54Z46	DDB_G0277783_TE	DDT-A	2.21	0.001
196	DDB_G0279721	P0CG81	ubqH	ubiquitin H	2.21	0.000
197	DDB_G0282867	Q54RW8	DDB_G0282867	unknown	2.21	0.000
198	DDB_G0291121	P14326	cinB	esterase/lipase/thioesterase domain-containing protein, vegetative specific protein H5	2.21	0.000
199	DDB_G0280813	Q54UU9	DDB_G0280813	phosphatidylinositol 3-kinase, FYVE-type zinc finger-containing protein	2.20	0.000
200	DDB_G0285983	Q54MG0	lsr2	long serine homopolymer repeat protein 2	2.20	0.001
201	DDB_G0274165	Q86KD2	DDB_G0274165	unknown	2.20	0.000
202	DDB_G0281679	Q54TL7	DDB_G0281679	RING zinc finger-containing protein	2.20	0.000
203	DDB_G0291658	Q54ES2	DDB_G0291658	unknown	2.19	0.001
204	DDB_G0269112	P22698	celB	cellulose-binding protein	2.19	0.001
205	DDB_G0273615	Q557E4	fpaB-2	ubiquitin ligase subunit SKP1	2.19	0.001
206	DDB_G0278231	Q54YH8	DDB_G0278231	unknown	2.19	0.000
207	DDB_G0284781	Q54P56	DDB_G0284781	unknown	2.17	0.001
208	DDB_G0280921	Q54UN7	DDB_G0280921	Phox domain-containing protein	2.16	0.000
209	DDB_G0294577	Q1ZXG2	DDB_G0294577	Rab GTPase	2.16	0.001
210	DDB_G0272955	Q559E7	DDB_G0272955	putative phytanoyl-CoA dioxygenase	2.16	0.000
211	DDB_G0274707	Q555N8	gpt5	putative glycophosphotransferase	2.15	0.001
212	DDB_G0280513	Q54V91	DDB_G0280513	unknown	2.15	0.000

213	DDB_G0281555	Q54TL0	kif7	kinesin family member 7	2.15	0.000
214	DDB_G0286287	Q54LZ7	DDB_G0286287	unknown	2.15	0.000
215	DDB_G0272218	Q75JX5	DDB_G0272218	unknown	2.14	0.000
216	DDB_G0286169	Q1ZXE7	rabZ	Rab GTPase	2.14	0.001
217	DDB_G0277531	Q54ZQ4	DDB_G0277531	EGF-like domain-containing protein	2.13	0.001
218	DDB_G0285485	Q54N59	DDB_G0285485	P-loop containing nucleoside triphosphate hydrolase family protein	2.12	0.000
219	DDB_G0276697	Q550Z8	DDB_G0276697	unknown	2.12	0.001
220	DDB_G0279571	Q54WL1	DDB_G0279571	polymorphic membrane protein repeat-containing protein	2.12	0.000
221	DDB_G0272562	P16643	csbB	contact site B protein B	2.12	0.000
222	DDB_G0281571	Q54TS2	comE	ankyrin repeat-containing protein, FNIP repeat-containing protein	2.12	0.000
223	DDB_G0285299	Q54NF0	DDB_G0285299	xanthine/uracil permease family protein	2.12	0.001
224	DDB_G0276817	Q7KWL4	DDB_G0276817	unknown	2.11	0.001
225	DDB_G0275117	Q8T2T4	DDB_G0275117	unknown	2.11	0.001
226	DDB_G0289029	Q54I39	DDB_G0289029	IST1-like protein	2.11	0.000
227	DDB_G0281781	Q54TF3	DDB_G0281781	unknown	2.11	0.000
228	DDB_G0290975	Q54FB4	DDB_G0290975	alpha/beta hydrolase fold-3 domain-containing protein	2.11	0.000
229	DDB_G0290065	Q54GM1	DDB_G0290065	unknown	2.10	0.000
230	DDB_G0277153	Q76P13	DDB_G0277153	unknown	2.10	0.000
231	DDB_G0276601	Q86HE9	DDB_G0276601	unknown	2.10	0.001
232	DDB_G0274163	Q86KD3	DDB_G0274163	unknown	2.09	0.000
233	DDB_G0274517	Q86HL8	osbE	oxysterol binding family protein, member 5	2.09	0.001
234	DDB_G0272720	Q86IJ6	DDB_G0272720	unknown	2.09	0.000
235	DDB_G0275521	Q86IB2	DDB_G0275521	unknown	2.09	0.000
236	DDB_G0272560	P19198	capA-1	cAMP-binding protein	2.09	0.000
237	DDB_G0284843	Q54P36	DDB_G0284843	transmembrane protein	2.08	0.001
238	DDB_G0279191	Q54WZ2	vacB	vacuolin B, prohibitin domain-containing protein	2.08	0.000
239	DDB_G0268886	Q55EI3	vps13E	vacuolar protein sorting-associated protein 13 family protein	2.07	0.000
240	DDB_G0270490	Q55E76	DDB_G0270490	unknown	2.07	0.001
241	DDB_G0284765	Q54P66	DDB_G0284765	DUF3430 family protein	2.07	0.001
242	DDB_G0269202	Q8STF9	gdcA	gp64 and disintegrin-like, cysteine-rich protein	2.06	0.000
243	DDB_G0270914	Q55DD9	DDB_G0270914	unknown	2.06	0.001
244	DDB_G0269462	Q55DZ2	DDB_G0269462	ubiquitin domain-containing protein	2.06	0.000
245	DDB_G0272767	Q86L42	DDB_G0272767	unknown	2.06	0.000
246	DDB_G0271104	Q55BJ7	DDB_G0271104	unknown	2.05	0.002
247	DDB_G0290109	Q54GJ9	DDB_G0290109	unknown	2.05	0.000
248	DDB_G0289227	Q54HU0	DDB_G0289227	unknown	2.05	0.001
249	DDB_G0282639	Q54S71	DDB_G0282639	unknown	2.04	0.000
250	DDB_G0277811	Q1ZXJ7	cfaB	counting factor associated protein	2.04	0.000
251	DDB_G0287879	Q54JX4	DDB_G0287879	unknown	2.04	0.000
252	DDB_G0272833	P16642	csbA	contact site B protein A	2.04	0.000
253	DDB_G0269458	P0CG88	ubqJ	ubiquitin J	2.04	0.001
254	DDB_G0291518	Q54EG8	DDB_G0291518	putative transmembrane protein	2.04	0.000

255	DDB_G0293056	Q54CC3	DDB_G0293056	Glucose-repressible alcohol dehydrogenase transcriptional effector	2.03	0.001
256	DDB_G0281515	Q54TT2	DDB_G0281515	equilibrative nucleoside transporter (ENT) family protein	2.03	0.002
257	DDB_G0293910	Q54B50	DDB_G0293910	unknown	2.03	0.002
258	DDB_G0290969	Q54FC2	DDB_G0290969	unknown	2.03	0.000
259	DDB_G0276347	Q8T127	DDB_G0276347	microtubule interacting and transport domain-containing protein (MIT)	2.03	0.000
260	DDB_G0292120	Q54DM8	cnrK	RING zinc finger-containing protein, putative cell number regulator	2.03	0.000
261	DDB_G0284345	Q54PT3	cyp556A1	cytochrome P450 family protein	2.03	0.002
262	DDB_G0281793	Q54TE4	DDB_G0281793	unknown	2.03	0.000
263	DDB_G0281395	Q54U11	DDB_G0281395	unknown	2.03	0.002
264	DDB_G0293720	Q54BJ0	DDB_G0293720	unknown	2.03	0.000
265	DDB_G0286887	Q54L57	DDB_G0286887	unknown	2.02	0.002
266	DDB_G0283113	Q54RJ2	eriA	putative RNase III, RNA exonuclease	2.02	0.000
267	DDB_G0287593	Q8T6H3	abcC6	ABC transporter C family protein	2.02	0.001
268	DDB_G0272843	Q558S9	DDB_G0272843	unknown	2.02	0.001
269	DDB_G0279705	Q54WE4	aplE	amoebapore-like protein E	2.01	0.003
270	DDB_G0282329	Q54SN9	DDB_G0282329	unknown	2.01	0.003
271	DDB_G0275799	Q553I2	DDB_G0275799	unknown	2.01	0.000
272	DDB_G0288489	Q54IV8	spoA	spore-specific protein A	2.01	0.003
273	DDB_G0276383	Q551R4	DDB_G0276383	putative guanylate cyclase	2.01	0.000
274	DDB_G0272704	Q86A22	cyp515A1	cytochrome P450 family protein	2.01	0.002
275	DDB_G0273009	Q86L39	DDB_G0273009	unknown	2.01	0.000
276	DDB_G0278649	Q54YD3	iliK	TatD-related deoxyribonuclease	2.00	0.000
277	DDB_G0272929	Q86II1	isg12	unknown	2.00	0.000
278	DDB_G0269450	Q55E02	DDB_G0269450	unknown	2.00	0.000
279	DDB_G0279817	Q54W93	DDB_G0279817	unknown	2.00	0.001

Down-regulated genes in ATG9¯ versus AX2 cells

#	DDB_G ID	UniProt ID	GeneName	GeneProduct	FC	p-value
1	DDB_G0293762	Q54BC0	DDB_G0293762	carbohydrate-binding domain-containing protein	-6.52	0.000
2	DDB_G0292102	Q54DP2	fscJ	GPCR family protein, frizzled and smoothened-like sans CRD protein	-5.10	0.000
3	DDB_G0267252	Q55H30	DDB_G0267252	unknown	-3.16	0.000
4	DDB_G0270058	Q55CG8	DDB_G0270058	unknown	-2.88	0.000
5	DDB_G0283727	Q54QN6	DDB_G0283727	short-chain dehydrogenase/reductase (SDR) family protein	-2.85	0.000
6	DDB_G0290779	Q54FK7	rabL	GTP binding protein RARE7L, Rab GTPase	-2.74	0.000
7	DDB_G0268838	Q55EL6	DDB_G0268838	unknown	-2.74	0.000
8	DDB_G0292098	Q54DP4	DDB_G0292098	unknown	-2.64	0.000
9	DDB_G0271624	Q55AX3	DDB_G0271624	unknown	-2.55	0.000
10	DDB_G0291616	Q54EC4	DDB_G0291616	unknown	-2.53	0.000
11	DDB_G0267258	Q55H27	DDB_G0267258_RTE	DIRS1 ORF2 fragment	-2.48	0.000
12	DDB_G0284295	Q54PU0	iliG	Endo-1,4-beta-glucanase family protein, glycoside hydrolase family 9 protein	-2.45	0.000
13	DDB_G0292094	Q54DP8	DDB_G0292094	unknown	-2.44	0.000
14	DDB_G0270922	Q55DB9	DDB_G0270922	unknown	-2.41	0.000

15	DDB_G0274177	Q86AS3	DDB_G0274177	EGF-like domain-containing protein	-2.38	0.000
16	DDB_G0278289	Q1ZXI9	fslO	frizzled and smoothened-like protein O	-2.37	0.000
17	DDB_G0276513	Q551I0	DDB_G0276513	unknown	-2.36	0.000
18	DDB_G0276037	Q8MNM6	DDB_G0276037	zinc-containing alcohol dehydrogenase (ADH)	-2.35	0.000
19	DDB_G0291123	P34114	glpD	glycogen phosphorylase 2	-2.34	0.000
20	DDB_G0274161	Q86KD5	DDB_G0274161	nmrA-like family protein	-2.34	0.000
21	DDB_G0290887	Q54FF6	DDB_G0290887	unknown	-2.32	0.000
22	DDB_G0289629	Q54H83	DDB_G0289629	unknown	-2.31	0.000
23	DDB_G0268352	Q55G63	DDB_G0268352	EGF-like domain-containing protein	-2.31	0.000
24	DDB_G0272026	Q86JM1	DDB_G0272026	unknown	-2.28	0.000
25	DDB_G0272466	Q559N8	DDB_G0272466	short-chain dehydrogenase/reductase (SDR) family protein, glucose/ribitol dehydrogenase family protein	-2.27	0.000
26	DDB_G0288219	Q54J86	DDB_G0288219	unknown	-2.26	0.000
27	DDB_G0275077	Q869X2	pks17	fatty acid synthase Pks17	-2.25	0.000
28	DDB_G0285323	Q54NA3	atg9	autophagy protein 9	-2.24	0.000
29	DDB_G0282039	Q54T21	DDB_G0282039	unknown	-2.21	0.000
30	DDB_G0276671	Q551B6	DDB_G0276671	unknown	-2.21	0.000
31	DDB_G0289765	Q54H11	DDB_G0289765	unknown	-2.20	0.001
32	DDB_G0286717	Q54LC2	ponC1	putative actin binding protein, ponticulin-related protein	-2.19	0.000
33	DDB_G0285687	Q54MS8	DDB_G0285687	unknown	-2.18	0.001
34	DDB_G0267244	Q55H34	DDB_G0267244_RTE	DIRS1 ORF2 fragment	-2.18	0.000
35	DDB_G0288103	Q54JE1	sibB	type A von Willebrand factor (VWFA) domain-containing protein	-2.16	0.000
36	DDB_G0289649	Q54H78	DDB_G0289649	unknown	-2.13	0.001
37	DDB_G0279383	Q54WV1	DDB_G0279383	unknown	-2.13	0.001
38	DDB_G0274499	B0G109	DDB_G0274499	patatin family protein	-2.13	0.000
39	DDB_G0278681	Q54Y58	DDB_G0278681	unknown	-2.11	0.000
40	DDB_G0279455	Q54WT2	DDB_G0279455	unknown	-2.10	0.001
41	DDB_G0269206	Q8T673	abcG21	ABC transporter G family protein	-2.06	0.001
42	DDB_G0287665	Q54K31	DDB_G0287665	unknown	-2.05	0.001
43	DDB_G0291354	Q54ET1	DDB_G0291354	unknown	-2.05	0.000
44	DDB_G0293456	Q54BS6	DDB_G0293456	unknown	-2.04	0.002
45	DDB_G0267322	Q55GZ6	DDB_G0267322_RTE	DIRS1 ORF2 fragment	-2.04	0.001
46	DDB_G0283559	Q54QW9	DDB_G0283559	unknown	-2.04	0.002
47	DDB_G0283143	Q54RJ9	DDB_G0283143	unknown	-2.04	0.000
48	DDB_G0280881	Q54UR0	DDB_G0280881	putative glutathione S-transferase	-2.04	0.000
49	DDB_G0286723	Q54LB9	ponC5	putative actin binding protein, ponticulin-related protein	-2.03	0.000
50	DDB_G0294246	Q54AS3	DDB_G0294246_TE	Tdd-4	-2.03	0.000
51	DDB_G0290423	Q54G39	DDB_G0290423	major facilitator superfamily protein	-2.02	0.000
52	DDB_G0290349	Q54GB7	DDB_G0290349	unknown	-2.01	0.003
53	DDB_G0279953	Q54W29	DDB_G0279953	unknown	-2.00	0.003

Up-regulated genes in ATG16⁻ versus AX2 cells

#	DDB_G ID	UniProt ID	GeneName	GeneProduct	FC	p-value
1	DDB_G0278391	Q54Y65	DDB_G0278391	unknown	22.21	0.000
2	DDB_G0288573	Q54IR6	DDB_G0288573	unknown	14.34	0.000
3	DDB_G0275487	Q86ID4	DDB_G0275487	unknown	13.07	0.000
4	DDB_G0279995	Q54VZ5	DDB_G0279995	putative glycoside hydrolase	12.96	0.000
5	DDB_G0276097	Q75JI6	DDB_G0276097	putative transmembrane protein	12.72	0.000
6	DDB_G0293202	Q54C11	trafH	TNF receptor-associated factor H	12.03	0.000
7	DDB_G0271140	Q8T6J0	abcA7	ABC transporter A family protein	11.33	0.000
8	DDB_G0281817	Q54TF4	DDB_G0281817	unknown	11.00	0.000
9	DDB_G0288571	Q54IR7	DDB_G0288571	unknown	10.73	0.000
10	DDB_G0271920	Q86I91	DDB_G0271920	unknown	10.32	0.000
11	DDB_G0281345	Q54U30	DDB_G0281345	unknown	10.09	0.000
12	DDB_G0291714	Q8T9W4	abcB3	ABC transporter B family protein AbcB3	9.76	0.000
13	DDB_G0276219	Q552D6	DDB_G0276219	putative transmembrane protein	9.46	0.000
14	DDB_G0269482	Q55DY0	DDB_G0269482	unknown	9.35	0.000
15	DDB_G0289411	Q54HJ4	DDB_G0289411	unknown	9.27	0.000
16	DDB_G0284121	Q54Q26	DDB_G0284121	transmembrane protein	9.26	0.000
17	DDB_G0268208	Q55F86	DDB_G0268208	unknown	8.95	0.000
18	DDB_G0271138	Q55BC0	abcA8	ABC transporter A family protein	8.53	0.000
19	DDB_G0286551	Q54LH9	gerE	germination protein GerE	8.12	0.000
20	DDB_G0281691	Q54TL1	DDB_G0281691	unknown	7.96	0.000
21	DDB_G0290637	Q54FU4	DDB_G0290637	unknown	7.83	0.000
22	DDB_G0289787	Q54H60	DDB_G0289787	unknown	7.63	0.000
23	DDB_G0289681	Q54H65	DDB_G0289681	unknown	7.62	0.000
24	DDB_G0292188	Q54DL7	DDB_G0292188	unknown	7.50	0.000
25	DDB_G0279817	Q54W93	DDB_G0279817	unknown	7.35	0.000
26	DDB_G0288745	Q1ZXD1	DDB_G0288745	unknown	7.03	0.000
27	DDB_G0268318	Q55GE7	DDB_G0268318	unknown	6.68	0.000
28	DDB_G0268556	Q55FQ6	psiE	PA14 domain-containing protein	6.45	0.000
29	DDB_G0286911	Q54L47	DDB_G0286911	unknown	6.40	0.000
30	DDB_G0282333	Q54SN7	DDB_G0282333	endonuclease/exonuclease/phosphatase domain-containing protein	6.35	0.000
31	DDB_G0281753	Q54TH1	DDB_G0281753	unknown	6.34	0.000
32	DDB_G0283913	Q54QE8	DDB_G0283913	heat shock protein Hsp20 domain-containing protein	6.24	0.000
33	DDB_G0289507	Q54HF0	act25	actin	6.24	0.000
34	DDB_G0268874	Q55EI9	DDB_G0268874	unknown	6.22	0.000
35	DDB_G0285791	Q54MW2	DDB_G0285791	unknown	6.02	0.000
36	DDB_G0280615	Q54V50	DDB_G0280615	unknown	5.86	0.000
37	DDB_G0294577	Q1ZXG2	DDB_G0294577	Rab GTPase	5.78	0.000
38	DDB_G0274291	Q86AA1	lyT2-4	putative T4-like lysozyme 2	5.60	0.000
39	DDB_G0282175	Q54SW3	grlF	GPCR family 3 protein 6	5.53	0.000
40	DDB_G0280501	Q54V98	DDB_G0280501	unknown	5.42	0.000
41	DDB_G0275033	Q86I68	DDB_G0275033	unknown	5.41	0.000
42	DDB_G0277215	Q86K86	iptA	adenylate dimethylallyltransferase	5.33	0.000

43	DDB_G0274831	Q556F2	lyT1-4	putative T4-like lysozyme 1	5.27	0.000
44	DDB_G0284779	Q54P57	DDB_G0284779	unknown	5.22	0.000
45	DDB_G0282919	Q54RU5	DDB_G0282919_TE	Tdd-4	5.18	0.000
46	DDB_G0276603	Q86HE8	DDB_G0276603	unknown	5.17	0.000
47	DDB_G0286239	Q54M29	DDB_G0286239	alpha/beta hydrolase fold-1 domain-containing protein, serine hydrolase-like protein	4.91	0.000
48	DDB_G0272827	Q966R0	cbpI	EF-hand domain-containing protein	4.74	0.000
49	DDB_G0283553	Q54QX5	DDB_G0283553	patatin family protein	4.72	0.000
50	DDB_G0283979	Q54QA1	DDB_G0283979	unknown	4.68	0.000
51	DDB_G0292652	Q54CW8	DDB_G0292652	unknown	4.67	0.000
52	DDB_G0290377	Q54G64	agnB	argonaut-like protein	4.66	0.000
53	DDB_G0283197	Q54RC8	DDB_G0283197	C2H2-type zinc finger-containing protein	4.61	0.000
54	DDB_G0349499	Q558U0	DDB_G0349499	unknown	4.59	0.000
55	DDB_G0267426	Q8MQU6	cshA	citrate synthase	4.54	0.000
56	DDB_G0290305	Q54G91	DDB_G0290305	unknown	4.49	0.000
57	DDB_G0276793	Q7KWW7	DDB_G0276793	unknown	4.48	0.000
58	DDB_G0276601	Q86HE9	DDB_G0276601	unknown	4.41	0.000
59	DDB_G0288419	Q54IY9	DDB_G0288419	unknown	4.41	0.000
60	DDB_G0287097	Q54KU2	DDB_G0287097	FNIP repeat-containing protein	4.39	0.000
61	DDB_G0282353	Q54SN0	cyp513E1	cytochrome P450 family protein	4.38	0.000
62	DDB_G0282171	Q54SW5	DDB_G0282171	unknown	4.35	0.000
63	DDB_G0290931	Q54FD5	trafL	TNF receptor-associated factor L	4.32	0.000
64	DDB_G0267896	Q55FZ1	DDB_G0267896	unknown	4.29	0.000
65	DDB_G0281135	Q54UD9	DDB_G0281135	unknown	4.28	0.000
66	DDB_G0282293	Q6TMI9	rnpA	RNA recognition motif-containing protein RRM, putative RNA binding protein	4.26	0.000
67	DDB_G0290841	Q54FI2	DDB_G0290841	unknown	4.22	0.000
68	DDB_G0272720	Q86IJ6	DDB_G0272720	unknown	4.19	0.000
69	DDB_G0276479	Q86HV8	ctnC	countin3	4.19	0.000
70	DDB_G0267612	Q55GM2	DDB_G0267612	unknown	4.18	0.000
71	DDB_G0272714	Q86II9	DDB_G0272714	unknown	4.17	0.000
72	DDB_G0272506	Q7KWU5	DDB_G0272506	unknown	4.13	0.000
73	DDB_G0279307	Q54WZ3	vacC	vacuolin C, band 7 family protein	4.10	0.000
74	DDB_G0289505	Q54HF1	act24	actin	4.03	0.000
75	DDB_G0283595	Q54QX7	DDB_G0283595	patatin family protein	4.02	0.000
76	DDB_G0278679	Q1ZXI7	cyp513F1	cytochrome P450 family protein	4.00	0.000
77	DDB_G0278647	Q54YE1	tps7	terpene synthase 7	3.97	0.000
78	DDB_G0278177	Q54YM0	DDB_G0278177	unknown	3.90	0.000
79	DDB_G0281087	Q54UG8	gtaV	GATA zinc finger domain-containing protein 22	3.88	0.000
80	DDB_G0291197	Q54F11	hbx3	homeobox transcription factor Hbx3	3.80	0.000
81	DDB_G0270990	Q55CT6	DDB_G0270990	putative acyl-CoA oxidase	3.78	0.000
82	DDB_G0289675	Q54H71	adprh	ADP-ribosylarginine hydrolase	3.76	0.000
83	DDB_G0273051	Q95ZG5	drnA-1	putative RNAse III DrnA	3.74	0.000
84	DDB_G0268142	Q55FF1	DDB_G0268142	unknown	3.66	0.000
85	DDB_G0272769	Q86L41	DDB_G0272769	unknown	3.64	0.000

86	DDB_G0291760	Q54E80	DDB_G0291760	unknown	3.57	0.000
87	DDB_G0281781	Q54TF3	DDB_G0281781	unknown	3.57	0.000
88	DDB_G0291758	Q54E82	DDB_G0291758	unknown	3.56	0.000
89	DDB_G0289723	Q54H38	abhd	alpha/beta hydrolase fold-1 domain-containing protein	3.52	0.000
90	DDB_G0280425	Q54VE1	DDB_G0280425	unknown	3.52	0.000
91	DDB_G0271134	P22699	celA	cellulase 270-6	3.51	0.000
92	DDB_G0288489	Q54IV8	spoA	spore-specific protein A	3.51	0.000
93	DDB_G0290221	Q54GD9	arv1	arv1-like family protein	3.50	0.000
94	DDB_G0275481	Q86ID7	DDB_G0275481	unknown	3.50	0.000
95	DDB_G0278613	Q54YP0	DDB_G0278613	EGF-like domain-containing protein, matrilin-like protein	3.46	0.000
96	DDB_G0275171	Q86I15	DDB_G0275171	unknown	3.44	0.000
97	DDB_G0289467	Q58A41	DD8-14	AAA ATPase domain-containing protein	3.43	0.000
98	DDB_G0286169	Q1ZXE7	rabZ	Rab GTPase	3.42	0.000
99	DDB_G0272829	Q86L54	DDB_G0272829	unknown	3.41	0.000
100	DDB_G0284925	Q54NY1	DDB_G0284925	unknown	3.36	0.000
101	DDB_G0291592	Q54EB8	DDB_G0291592	unknown	3.35	0.000
102	DDB_G0280375	Q54VR7	DDB_G0280375	unknown	3.34	0.000
103	DDB_G0277809	Q54Z64	cfaA	counting factor associated protein	3.32	0.000
104	DDB_G0282021	Q54T40	DDB_G0282021	unknown	3.31	0.000
105	DDB_G0271666	P11872	prtB	proteosomal alpha-subunit 7-1	3.30	0.000
106	DDB_G0272867	Q558Z0	argS1	arginyl-tRNA synthetase	3.28	0.000
107	DDB_G0293274	Q54C10	DDB_G0293274	B-box zinc finger-containing protein/FNIP repeat-containing protein	3.28	0.000
108	DDB_G0271914	Q8T1Z7	DDB_G0271914	CMP/dCMP deaminase, zinc-binding domain-containing protein	3.25	0.000
109	DDB_G0286129	Q54M93	DDB_G0286129	F-box/WD repeat-containing protein	3.25	0.000
110	DDB_G0289171	Q54HW8	DDB_G0289171	unknown	3.24	0.000
111	DDB_G0286983	Q54L10	DDB_G0286983	unknown	3.23	0.000
112	DDB_G0286637	Q54LH3	DDB_G0286637	3-methyl-2-oxobutanoate hydroxyl-methyltransferase, 2-dehydropantoate 2-reductase	3.20	0.000
113	DDB_G0284931	Q54NX8	DDB_G0284931	unknown	3.20	0.000
114	DDB_G0282737	Q54S05	DDB_G0282737	unknown	3.19	0.000
115	DDB_G0283911	Q54QE9	hsp69	heat shock protein 69	3.19	0.000
116	DDB_G0288417	Q54IZ0	DDB_G0288417	unknown	3.18	0.000
117	DDB_G0285539	Q54N32	DDB_G0285539	NUDIX hydrolase family protein	3.17	0.000
118	DDB_G0282441	Q54SI8	DDB_G0282441	unknown	3.17	0.000
119	DDB_G0270700	Q55CM2	DDB_G0270700	calcium-binding EGF domain-containing protein	3.17	0.000
120	DDB_G0274637	Q554W2	DDB_G0274637	unknown	3.17	0.000
121	DDB_G0284295	Q54PU0	iliG	Endo-1,4-beta-glucanase family protein, glycoside hydrolase family 9 protein	3.16	0.000
122	DDB_G0284615	Q54PE3	DDB_G0284615	EGF-like domain-containing protein	3.16	0.000
123	DDB_G0272194	Q86IM3	DDB_G0272194	unknown	3.16	0.000
124	DDB_G0280503	Q54V97	DDB_G0280503	unknown	3.16	0.000
125	DDB_G0294515	N/A	dutA	structural RNA	3.16	0.000
126	DDB_G0293720	Q54BJ0	DDB_G0293720	unknown	3.15	0.000
127	DDB_G0272724	Q86IK1	DDB_G0272724	unknown	3.14	0.000

128	DDB_G0285299	Q54NF0	DDB_G0285299	xanthine/uracil permease family protein	3.12	0.000
129	DDB_G0278011	Q1ZXJ6	racQ	Rho GTPase	3.12	0.000
130	DDB_G0293208	Q54C15	DDB_G0293208	unknown	3.10	0.000
131	DDB_G0274477	Q86HQ5	DDB_G0274477	unknown	3.10	0.000
132	DDB_G0277265	Q86AP3	DDB_G0277265	unknown	3.09	0.000
133	DDB_G0278115	Q54YR8	netD	nuclear envelope transmembrane protein 4	3.06	0.000
134	DDB_G0271472	Q55B19	DDB_G0271472	unknown	3.06	0.000
135	DDB_G0284619	Q54PE1	DDB_G0284619	unknown	3.06	0.000
136	DDB_G0285289	Q54NF4	spoB	spore-specific protein B	3.06	0.000
137	DDB_G0270138	Q55CB0	rasU	Ras GTPase RasU	3.04	0.000
138	DDB_G0270140	Q55CA9	rasZ	Ras GTPase RasZ	3.04	0.000
139	DDB_G0286907	P0CG77	ubqD	ubiquitin D	3.04	0.000
140	DDB_G0269474	Q1ZXQ4	fcsB	fatty acyl-CoA synthetase	3.03	0.000
141	DDB_G0273451	Q557Q0	DDB_G0273451	AAA+ ATPase, core domain-containing protein	3.03	0.000
142	DDB_G0281853	Q54TD1	iliL	putative cell surface glycoprotein	3.02	0.000
143	DDB_G0288033	Q54JI3	DDB_G0288033	unknown	3.02	0.000
144	DDB_G0279707	Q54WE3	iliP	DUF3430 family protein	3.01	0.000
145	DDB_G0291646	Q54EB9	DDB_G0291646	unknown	3.01	0.000
146	DDB_G0291748	Q54E87	DDB_G0291748	unknown	3.00	0.000
147	DDB_G0268144	Q6B9X6	vwkA	alpha protein kinase VwkA	2.99	0.000
148	DDB_G0286153	Q54M68	DDB_G0286153	unknown	2.99	0.000
149	DDB_G0271576	Q55AV5	DDB_G0271576_RTE	TRE3-B ORF2	2.98	0.000
150	DDB_G0284921	Q54NY4	DDB_G0284921	unknown	2.97	0.000
151	DDB_G0272150	Q75JT4	grlJ	GPCR family 3 protein 9	2.96	0.000
152	DDB_G0267578	Q55GP1	DDB_G0267578	unknown	2.96	0.000
153	DDB_G0272947	Q86IJ4	DDB_G0272947	unknown	2.95	0.000
154	DDB_G0282991	Q54RQ2	DDB_G0282991	unknown	2.95	0.000
155	DDB_G0289915	Q54GU6	DDB_G0289915	Phospholipid scramblase 3	2.95	0.000
156	DDB_G0272666	Q86JC1	DDB_G0272666	putative ankyrin repeat protein	2.94	0.000
157	DDB_G0286887	Q54L57	DDB_G0286887	unknown	2.92	0.000
158	DDB_G0289029	Q54I39	DDB_G0289029	IST1-like protein	2.92	0.000
159	DDB_G0275689	Q9NGP5	abcG2	ABC transporter G family protein	2.90	0.000
160	DDB_G0292506	Q54D31	DDB_G0292506	unknown	2.90	0.000
161	DDB_G0269202	Q8STF9	gdcA	gp64 and disintegrin-like, cysteine-rich protein	2.88	0.000
162	DDB_G0278173	Q54YM2	DDB_G0278173	unknown	2.88	0.000
163	DDB_G0288623	Q966Q9	cbpH	calcium-binding protein	2.88	0.000
164	DDB_G0290885	Q54FF8	DDB_G0290885	unknown	2.87	0.000
165	DDB_G0272949	Q559F5	bcas3	putative proppin	2.86	0.000
166	DDB_G0272442	Q559S7	DDB_G0272442	unknown	2.86	0.000
167	DDB_G0285833	Q54MP6	DDB_G0285833	unknown	2.84	0.000
168	DDB_G0288219	Q54J86	DDB_G0288219	unknown	2.83	0.000
169	DDB_G0268892	Q55EH9	DDB_G0268892	unknown	2.83	0.000
170	DDB_G0280919	Q54UN8	DDB_G0280919	unknown	2.83	0.000
171	DDB_G0288887	Q54IB0	DDB_G0288887	unknown	2.82	0.000

172	DDB_G0284781	Q54P56	DDB_G0284781	unknown	2.82	0.000
173	DDB_G0289917	Q54GU5	DDB_G0289917	unknown	2.82	0.000
174	DDB_G0281395	Q54U11	DDB_G0281395	unknown	2.81	0.000
175	DDB_G0276361	Q86AC9	DDB_G0276361	unknown	2.81	0.000
176	DDB_G0272955	Q559E7	DDB_G0272955	putative phytanoyl-CoA dioxygenase	2.80	0.000
177	DDB_G0274335	Q86IY2	DDB_G0274335	unknown	2.79	0.000
178	DDB_G0277863	P22549	pdiA	cAMP phosphodiesterase inhibitor	2.79	0.000
179	DDB_G0279571	Q54WL1	DDB_G0279571	polymorphic membrane protein repeat-containing protein	2.79	0.000
180	DDB_G0290381	Q54G62	DDB_G0290381	unknown	2.79	0.000
181	DDB_G0274337	Q86IY3	DDB_G0274337	unknown	2.78	0.000
182	DDB_G0279119	Q54X91	DDB_G0279119	unknown	2.78	0.000
183	DDB_G0288791	Q54IF5	hlcs1	biotin--[acetyl-CoA-carboxylase] ligase 1	2.78	0.000
184	DDB_G0282141	P13231	hatA	actin binding protein, hisactophilin I	2.78	0.000
185	DDB_G0285225	Q54NI0	DDB_G0285225	unknown	2.77	0.000
186	DDB_G0291772	Q54E81	DDB_G0291772	unknown	2.77	0.000
187	DDB_G0270306	Q55BY9	DDB_G0270306	putative transcriptional regulator	2.77	0.000
188	DDB_G0285215	Q54NJ3	DDB_G0285215	unknown	2.77	0.000
189	DDB_G0287047	Q54KX8	DDB_G0287047	unknown	2.76	0.000
190	DDB_G0270490	Q55E76	DDB_G0270490	unknown	2.76	0.000
191	DDB_G0277989	Q54YZ5	DDB_G0277989	putative protein kinase	2.75	0.000
192	DDB_G0272919	Q1ZXN2	DDB_G0272919	unknown	2.75	0.000
193	DDB_G0292518	Q54DB0	DDB_G0292518	hssA/2C/7E family protein	2.73	0.000
194	DDB_G0282209	Q54SU8	DDB_G0282209	type A von Willebrand factor (VWFA) domain-containing protein	2.73	0.000
195	DDB_G0290993	Q54F96	DDB_G0290993	unknown	2.73	0.000
196	DDB_G0285775	Q54MW7	DDB_G0285775	putative transmembrane protein	2.72	0.000
197	DDB_G0272787	Q86B06	sds	L-serine ammonia-lyase, L-serine dehydratase, serine deaminase	2.72	0.000
198	DDB_G0275253	Q8T2U1	DDB_G0275253	unknown	2.72	0.000
199	DDB_G0290959	Q54FB1	chtC	cheater C	2.72	0.000
200	DDB_G0284917	Q54NY6	DDB_G0284917	unknown	2.71	0.000
201	DDB_G0272508	Q7KWU2	DDB_G0272508	unknown	2.70	0.001
202	DDB_G0272744	Q7KWP9	DDB_G0272744	hssA/2C/7E family protein	2.70	0.000
203	DDB_G0274023	P19198	capA-2	cAMP-binding protein	2.69	0.000
204	DDB_G0274613	Q554L2	DDB_G0274613	RING zinc finger-containing protein, putative protein serine/threonine kinase, CMGC group	2.69	0.000
205	DDB_G0285165	Q54NL1	abcC9	ABC transporter C family protein	2.68	0.000
206	DDB_G0285627	Q54MW9	DDB_G0285627	unknown	2.67	0.000
207	DDB_G0272993	Q558Z4	DDB_G0272993	unknown	2.64	0.000
208	DDB_G0273017	Q558S7	DDB_G0273017	isocitrate lyase	2.64	0.000
209	DDB_G0272718	Q86IJ5	DDB_G0272718	unknown	2.64	0.000
210	DDB_G0272560	P19198	capA-1	cAMP-binding protein	2.64	0.000
211	DDB_G0288713	Q54IJ5	DDB_G0288713	unknown	2.64	0.001
212	DDB_G0292858	Q54CM2	DDB_G0292858	patatin family protein	2.63	0.000
213	DDB_G0277791	Q54Z85	racO	Rho GTPase RacO	2.63	0.000
214	DDB_G0283429	Q54R22	DDB_G0283429	unknown	2.63	0.000

215	DDB_G0283281	Q54RB1	DDB_G0283281	strictosidine synthase family protein	2.62	0.000
216	DDB_G0285923	Q54MI4	DDB_G0285923	unknown	2.62	0.000
217	DDB_G0275799	Q553I2	DDB_G0275799	unknown	2.61	0.000
218	DDB_G0271802	Q75JD9	DDB_G0271802	unknown	2.60	0.000
219	DDB_G0289677	Q54H69	hbx14	homeobox transcription factor Hbx14	2.60	0.000
220	DDB_G0276905	Q86JA8	DDB_G0276905	unknown	2.60	0.000
221	DDB_G0267894	Q55FZ2	DDB_G0267894	unknown	2.59	0.000
222	DDB_G0272558	Q559H7	DDB_G0272558	unknown	2.59	0.000
223	DDB_G0277389	Q75J87	DDB_G0277389	unknown	2.59	0.000
224	DDB_G0275521	Q86IB2	DDB_G0275521	unknown	2.58	0.000
225	DDB_G0275161	Q86I43	DDB_G0275161	unknown	2.58	0.000
226	DDB_G0287793	Q54JU2	DDB_G0287793	glutathione S-transferase, glutathione transferase	2.57	0.000
227	DDB_G0279191	Q54WZ2	vacB	vacuolin B, prohibitin domain-containing protein	2.57	0.000
228	DDB_G0291796	Q54E55	araA	putative regulator of adhesion and motility 4	2.57	0.000
229	DDB_G0292996	P03967	rasD	Ras GTPase RasD	2.56	0.001
230	DDB_G0289043	Q54I29	DDB_G0289043	unknown	2.56	0.000
231	DDB_G0286287	Q54LZ7	DDB_G0286287	unknown	2.56	0.000
232	DDB_G0270750	Q55C86	DDB_G0270750	Kelch repeat-containing protein	2.56	0.000
233	DDB_G0282059	Q54T10	DDB_G0282059	unknown	2.56	0.000
234	DDB_G0286673	Q54LE9	DDB_G0286673	unknown	2.55	0.000
235	DDB_G0281079	Q54UH2	DDB_G0281079	unknown	2.55	0.000
236	DDB_G0279721	P0CG81	ubqH	ubiquitin H	2.55	0.000
237	DDB_G0271852	Q86HF8	rtaA	lipid-translocating exporter family protein RtaA	2.54	0.000
238	DDB_G0269112	P22698	celB	cellulose-binding protein	2.54	0.000
239	DDB_G0286227	Q54M37	DDB_G0286227	unknown	2.53	0.000
240	DDB_G0268802	Q55EP5	gpaJ	G-protein subunit alpha 10	2.52	0.000
241	DDB_G0288935	Q54I80	panC	pantoate-beta-alanine ligase	2.51	0.000
242	DDB_G0274339	Q86IY4	DDB_G0274339	acetyl-CoA C-acyltransferase, beta-ketothiolase, 3-ketoacyl-CoA thiolase	2.51	0.000
243	DDB_G0289395	Q6UUW5	crlB	cAMP receptor-like protein, G-protein-coupled receptor (GPCR) family protein	2.50	0.000
244	DDB_G0274655	Q555C4	rigA	unknown	2.50	0.000
245	DDB_G0267702	Q55GE8	DDB_G0267702	unknown	2.50	0.001
246	DDB_G0272262	Q559Z7	DDB_G0272262	unknown	2.49	0.001
247	DDB_G0291430	Q54EN6	DDB_G0291430	unknown	2.49	0.000
248	DDB_G0272742	Q7KWP5	DDB_G0272742	unknown	2.49	0.000
249	DDB_G0269664	Q55DG8	DDB_G0269664	unknown	2.48	0.000
250	DDB_G0275117	Q8T2T4	DDB_G0275117	unknown	2.48	0.000
251	DDB_G0291716	Q54E65	DDB_G0291716	unknown	2.47	0.000
252	DDB_G0286485	Q54LQ8	DDB_G0286485	PHO85 cyclin-2	2.47	0.000
253	DDB_G0274391	P10901	alfA	alpha-L-fucosidase	2.47	0.000
254	DDB_G0287765	Q54JW3	DDB_G0287765	NADH:flavin oxidoreductase/NADH oxidase domain-containing protein	2.46	0.001
255	DDB_G0292016	Q54DV3	DDB_G0292016	von Willebrand factor A domain-containing protein 5A	2.46	0.000
256	DDB_G0276177	N/A	N/A	N/A	2.46	0.000

257	DDB_G0282341	Q54SN3	DDB_G0282341	enoyl-CoA hydratase/isomerase family protein	2.45	0.000
258	DDB_G0277709	Q54ZA2	DDB_G0277709	unknown	2.45	0.001
259	DDB_G0272957	Q559E6	DDB_G0272957	Protein FRA10AC1	2.45	0.000
260	DDB_G0290177	Q54GG6	DDB_G0290177	putative transmembrane protein	2.44	0.000
261	DDB_G0287583	P15521	gerC	spore germination protein C	2.44	0.000
262	DDB_G0267640	Q55GK0	gtaE	GATA zinc finger domain-containing protein 5	2.44	0.000
263	DDB_G0272809	Q7KWN1	DDB_G0272809	unknown	2.44	0.000
264	DDB_G0289497	Q54HF8	DDB_G0289497	unknown	2.44	0.000
265	DDB_G0270652	Q55D22	DDB_G0270652	molybdenum cofactor sulfurase domain-containing protein	2.44	0.001
266	DDB_G0270212	Q58A40	DDB_G0270212	galactose-binding domain-containing protein	2.44	0.000
267	DDB_G0293948	Q54B19	DDB_G0293948	unknown	2.44	0.000
268	DDB_G0288737	Q54IJ4	DDB_G0288737	unknown	2.44	0.000
269	DDB_G0267476	Q6TMJ6	sigK	EGF-like domain-containing protein	2.43	0.000
270	DDB_G0277137	Q550E0	DDB_G0277137	SUR2-type hydroxylase/desaturase catalytic region-containing protein	2.43	0.000
271	DDB_G0272182	Q75JW5	DDB_G0272182	putative arginine deiminase	2.43	0.000
272	DDB_G0279023	Q54XE0	DDB_G0279023	unknown	2.41	0.000
273	DDB_G0269700	Q55DD2	DDB_G0269700	RCC1 and BTB domain-containing protein 1	2.41	0.000
274	DDB_G0287809	Q54JT1	DDB_G0287809	unknown	2.41	0.000
275	DDB_G0277921	Q54YR6	sigE	transmembrane protein	2.41	0.002
276	DDB_G0282567	Q54SA8	DDB_G0282567	unknown	2.41	0.001
277	DDB_G0277379	P54643	pspD	spore coat protein SP87	2.41	0.000
278	DDB_G0289113	Q54HZ6	DDB_G0289113	unknown	2.40	0.000
279	DDB_G0273013	Q1ZXM2	uglB	uracil glycosylase, uracil-DNA glycosylase	2.40	0.000
280	DDB_G0267790	Q55G73	DDB_G0267790	unknown	2.40	0.000
281	DDB_G0272835	Q86IG3	plip	phosphatidylinositol phosphatase, phosphoinositide phosphatase	2.39	0.000
282	DDB_G0275951	Q553A0	DDB_G0275951	putative transmembrane protein	2.39	0.001
283	DDB_G0289683	Q54H64	DDB_G0289683	unknown	2.39	0.001
284	DDB_G0280703	Q54UZ9	DDB_G0280703	elongation factor 1beta-related protein	2.39	0.000
285	DDB_G0274121	Q555Z5	abcA4	ABC transporter A family protein	2.39	0.000
286	DDB_G0291576	Q54EC9	DDB_G0291576	DUF781 family protein	2.38	0.002
287	DDB_G0278503	Q54XZ8	DDB_G0278503	unknown	2.38	0.000
288	DDB_G0276347	Q8T127	DDB_G0276347	microtubule interacting and transport domain-containing protein (MIT)	2.38	0.000
289	DDB_G0284345	Q54PT3	cyp556A1	cytochrome P450 family protein	2.38	0.000
290	DDB_G0293060	Q54CC1	DDB_G0293060	unknown	2.37	0.000
291	DDB_G0271722	Q75JE6	DDB_G0271722	putative extracellular matrix protein	2.37	0.000
292	DDB_G0295493	N/A	srpB	SRP RNA, signal recognition particle RNA	2.37	0.000
293	DDB_G0289473	Q54HG6	DDB_G0289473	P-type ATPase, Ca2+-ATPase	2.37	0.000
294	DDB_G0288877	Q54I71	aarA	beta-catenin related protein	2.37	0.000
295	DDB_G0279483	Q54WR4	pldB	phospholipase D1	2.37	0.000
296	DDB_G0295797	C7G076	DDB_G0295797	EGF-like domain-containing protein	2.36	0.003
297	DDB_G0274303	Q86J03	DDB_G0274303	CDGSH iron-sulfur domain-containing protein	2.36	0.000
298	DDB_G0267414	Q55G81	repD	transcription factor IIH component	2.36	0.000

299	DDB_G0292116	Q54DN0	DDB_G0292116	glucose/ribitol dehydrogenase family protein	2.35	0.000
300	DDB_G0272811	Q7KWP7	wasB	WASP-related protein B	2.35	0.002
301	DDB_G0290379	Q54G63	DDB_G0290379	unknown	2.35	0.000
302	DDB_G0281727	Q54TI5	DDB_G0281727	unknown	2.34	0.000
303	DDB_G0272736	Q7KWM8	tgrN1	immunoglobulin E-set domain-containing protein	2.34	0.000
304	DDB_G0272252	Q55A13	DDB_G0272252	unknown	2.34	0.000
305	DDB_G0272935	Q559G8	mocos	molybdenum cofactor sulfurase	2.33	0.000
306	DDB_G0273475	Q86AT9	dhkI-1	histidine kinase I	2.33	0.000
307	DDB_G0271916	P54681	rtoA	unknown	2.33	0.001
308	DDB_G0295485	B0G154	DDB_G0295485	EGF-like domain-containing protein	2.32	0.000
309	DDB_G0283465	Q54QZ9	DDB_G0283465	unknown	2.32	0.000
310	DDB_G0272823	Q86A15	DDB_G0272823	unknown	2.32	0.000
311	DDB_G0273383	Q557T0	cwc15-1	putative pre-mRNA-splicing factor CWC15	2.32	0.000
312	DDB_G0281387	Q54TY7	srfA	MADS-box transcription factor, SRF-related protein	2.32	0.000
313	DDB_G0282715	Q54S16	DDB_G0282715	Neutral and basic amino acid transport protein rBAT	2.32	0.000
314	DDB_G0288399	Q54J01	DDB_G0288399	unknown	2.32	0.002
315	DDB_G0285447	Q54N85	DDB_G0285447	putative transmembrane protein, DUF829 family protein	2.31	0.000
316	DDB_G0283077	Q54RL7	DDB_G0283077	putative glycoside hydrolase	2.31	0.003
317	DDB_G0294575	Q1ZXJ5	DDB_G0294575	ankyrin repeat-containing protein	2.31	0.000
318	DDB_G0276967	Q9GPS3	racF2	Rho GTPase RacF2	2.30	0.000
319	DDB_G0272728	Q86A30	DDB_G0272728	unknown	2.30	0.004
320	DDB_G0283505	Q54R32	DDB_G0283505	transmembrane protein	2.30	0.002
321	DDB_G0272841	Q558S4	DDB_G0272841	acid phosphatase	2.30	0.000
322	DDB_G0272674	Q86IF3	DDB_G0272674	unknown	2.30	0.000
323	DDB_G0290087	Q54GK9	vps2A	SNF7 family protein	2.30	0.000
324	DDB_G0286363	Q54LX4	DDB_G0286363	unknown	2.30	0.000
325	DDB_G0273615	Q557E4	fpaB-2	ubiquitin ligase subunit SKP1	2.30	0.004
326	DDB_G0272716	Q86IJ0	DDB_G0272716	putative transmembrane protein	2.29	0.004
327	DDB_G0269270	Q55EE4	DDB_G0269270	AhpC/TSA family protein	2.29	0.000
328	DDB_G0268784	Q55EQ7	DDB_G0268784	major facilitator superfamily 1 transporter (MFS-1)	2.28	0.003
329	DDB_G0280211	Q54VP7	DDB_G0280211	unknown	2.28	0.000
330	DDB_G0269018	Q55EK1	DDB_G0269018	unknown	2.27	0.000
331	DDB_G0281607	Q54TR0	DDB_G0281607	unknown	2.27	0.000
332	DDB_G0290215	Q54GE2	DDB_G0290215	unknown	2.27	0.000
333	DDB_G0268850	Q55EK5	DDB_G0268850	putative ATP binding protein	2.27	0.000
334	DDB_G0272682	Q86IG2	DDB_G0272682	endonuclease/exonuclease/phosphatase domain-containing protein, cry34 related protein	2.26	0.000
335	DDB_G0271936	Q55AE5	DDB_G0271936	unknown	2.26	0.000
336	DDB_G0286305	Q54LY4	DDB_G0286305	zinc-containing alcohol dehydrogenase (ADH)	2.25	0.000
337	DDB_G0284927	Q54NY0	DDB_G0284927	unknown	2.25	0.001
338	DDB_G0279493	Q54WQ8	DDB_G0279493	unknown	2.25	0.000
339	DDB_G0286537	Q54LN1	DDB_G0286537	unknown	2.25	0.000

340	DDB_G0272650	Q556H7	DDB_G0272650	unknown	2.25	0.002
341	DDB_G0282329	Q54SN9	DDB_G0282329	unknown	2.25	0.004
342	DDB_G0290969	Q54FC2	DDB_G0290969	unknown	2.25	0.000
343	DDB_G0268852	Q55EK4	DDB_G0268852	putative ATP binding protein	2.25	0.000
344	DDB_G0282995	Q54RQ0	DDB_G0282995	unknown	2.24	0.000
345	DDB_G0280621	Q54V47	arrJ	ADP-ribosylation factor-related, ARF-related	2.24	0.002
346	DDB_G0272925	Q559A0	gacU	RhoGAP domain-containing protein	2.24	0.000
347	DDB_G0282647	Q54S65	DDB_G0282647	unknown	2.24	0.000
348	DDB_G0273537	Q557I5	DDB_G0273537	unknown	2.24	0.005
349	DDB_G0270214	Q55C60	DD7-1	galactose-binding domain-containing protein	2.24	0.000
350	DDB_G0279791	Q54WC4	plnA	perilipin	2.23	0.000
351	DDB_G0270126	Q55CB7	rasY	Ras GTPase RasY	2.23	0.000
352	DDB_G0273009	Q86L39	DDB_G0273009	unknown	2.23	0.000
353	DDB_G0289733	Q54H33	DDB_G0289733	DUF962 family protein	2.22	0.000
354	DDB_G0272738	Q7KWM9	iunH	N-D-ribosylpurine ribohydrolase, inosine-uridine nucleoside N-ribohydrolase	2.22	0.000
355	DDB_G0272514	Q7KWT8	DDB_G0272514	unknown	2.21	0.000
356	DDB_G0287671	Q54K28	DDB_G0287671	unknown	2.21	0.004
357	DDB_G0272997	Q558Y5	DDB_G0272997	unknown	2.21	0.000
358	DDB_G0289641	Q54H89	DDB_G0289641	unknown	2.21	0.000
359	DDB_G0289487	P07829	act3	actin	2.21	0.000
360	DDB_G0292772	Q54CT6	tgrG1	immunoglobulin E-set domain-containing protein	2.20	0.006
361	DDB_G0281973	Q54T67	DDB_G0281973	unknown	2.20	0.006
362	DDB_G0286715	Q54LC3	ponC2	putative actin binding protein, ponticulin-related protein	2.20	0.002
363	DDB_G0275217	Q554H9	DDB_G0275217_RTE	TRE3-B ORF1	2.20	0.006
364	DDB_G0272855	Q558X8	DDB_G0272855	unknown	2.20	0.006
365	DDB_G0267580	Q55GP0	DDB_G0267580	unknown	2.20	0.001
366	DDB_G0272995	Q558Z1	DDB_G0272995	unknown	2.20	0.000
367	DDB_G0280513	Q54V91	DDB_G0280513	unknown	2.20	0.000
368	DDB_G0287133	Q54KT6	DDB_G0287133	unknown	2.19	0.000
369	DDB_G0272746	Q7KWQ0	DDB_G0272746	unknown	2.19	0.000
370	DDB_G0278765	Q54XT1	DDB_G0278765	unknown	2.19	0.000
371	DDB_G0282759	Q54SI2	DDB_G0282759	unknown	2.18	0.000
372	DDB_G0282121	Q54SZ0	DDB_G0282121	unknown	2.18	0.001
373	DDB_G0280217	Q54VP3	DDB_G0280217	unknown	2.17	0.007
374	DDB_G0291348	Q54ET4	DDB_G0291348	fungal transcriptional regulatory protein, putative zinc cluster transcription factor	2.17	0.007
375	DDB_G0272658	Q7KWN7	sigN1	SrfA-induced protein N1	2.17	0.007
376	DDB_G0270522	Q55E12	DDB_G0270522	unknown	2.17	0.000
377	DDB_G0276475	Q86HB8	cfaC	counting factor associated protein	2.17	0.000
378	DDB_G0284645	Q54PC5	DDB_G0284645	unknown	2.17	0.000
379	DDB_G0291706	Q54EA2	DDB_G0291706	unknown	2.16	0.008
380	DDB_G0281767	B0G136	DDB_G0281767	ankyrin repeat-containing protein, regulator of chromosome condensation (RCC1) domain-containing protein, BTB/POZ domain-containing protein	2.16	0.000

381	DDB_G0273439	Q86KI1	ap2a1-1	adaptor-related protein complex 2, alpha subunit, alpha adaptin	2.16	0.000
382	DDB_G0275197	Q1ZXL2	cyp518B1	cytochrome P450 family protein	2.16	0.008
383	DDB_G0273003	Q86L45	snrpD3	LSM (like-Sm) domain-containing protein, putative small nuclear ribonucleoprotein D3	2.16	0.000
384	DDB_G0293910	Q54B50	DDB_G0293910	unknown	2.16	0.000
385	DDB_G0285345	Q54ND0	DDB_G0285345	unknown	2.16	0.000
386	DDB_G0269254	Q6TMJ3	sigJ	unknown	2.16	0.000
387	DDB_G0281735	Q54TI1	DDB_G0281735	putative transmembrane protein	2.15	0.001
388	DDB_G0284787	Q54P53	DDB_G0284787	unknown	2.15	0.005
389	DDB_G0272785	Q86B07	DDB_G0272785	acyloxyacyl hydrolase	2.15	0.000
390	DDB_G0278389	Q54Y66	rsmF	small GTPase	2.15	0.008
391	DDB_G0268010	Q55FP3	DDB_G0268010	unknown	2.15	0.007
392	DDB_G0293038	Q54CD5	DDB_G0293038	unknown	2.15	0.000
393	DDB_G0290491	Q54G11	atg8b	autophagy protein 8b	2.15	0.000
394	DDB_G0268446	Q55FE6	cbhA	cellobiohydrolase A	2.15	0.001
395	DDB_G0270890	Q55DM9	DDB_G0270890	unknown	2.14	0.008
396	DDB_G0275251	Q86I10	DDB_G0275251	unknown	2.14	0.008
397	DDB_G0290489	Q54G12	DDB_G0290489	unknown	2.14	0.001
398	DDB_G0269510	Q55DV8	vps37	Modifier of rudimentary (Modr) family protein	2.14	0.000
399	DDB_G0269458	P0CG88	ubqJ	ubiquitin J	2.14	0.002
400	DDB_G0280813	Q54UU9	DDB_G0280813	phosphatidylinositol 3-kinase, FYVE-type zinc finger-containing protein	2.14	0.000
401	DDB_G0280633	Q54V41	arrK	ADP-ribosylation factor-related, ARF-related	2.13	0.000
402	DDB_G0286277	Q54M04	DDB_G0286277	putative glycoside hydrolase	2.13	0.000
403	DDB_G0272983	Q559A6	DDB_G0272983	DNAJ heat shock N-terminal domain-containing protein	2.13	0.000
404	DDB_G0277793	Q54Z79	DDB_G0277793	unknown	2.13	0.002
405	DDB_G0294338	Q54AM7	DDB_G0294338_RTE	DGLT-A	2.13	0.008
406	DDB_G0279159	Q54X73	aco1	putative iron regulatory protein, aconitate hydratase, aconitase	2.12	0.000
407	DDB_G0270836	Q6RYT0	fbp	D-fructose-1,6-bisphosphate 1-phospho-hydrolase, fructose-1,6-bisphosphatase	2.12	0.000
408	DDB_G0272863	Q558Y8	DDB_G0272863	unknown	2.12	0.000
409	DDB_G0272819	P54658	hspC	heat shock protein C	2.12	0.000
410	DDB_G0269256	Q55EE4	DDB_G0269256	AhpC/TSA family protein	2.12	0.000
411	DDB_G0277147	Q550D5	stkA	GATA-binding transcription factor, GATA zinc finger domain-containing protein 1	2.12	0.000
412	DDB_G0279757	Q54WC3	DDB_G0279757	unknown	2.11	0.010
413	DDB_G0271488	Q55B11	vta1	unknown	2.11	0.000
414	DDB_G0290531	Q54FY5	DDB_G0290531	unknown	2.11	0.000
415	DDB_G0285929	Q54MI0	DDB_G0285929	unknown	2.10	0.009
416	DDB_G0287687	P15520	gerB	spore germination protein B	2.10	0.007
417	DDB_G0272865	Q558Y9	DDB_G0272865	unknown	2.10	0.000
418	DDB_G0271340	Q55BH8	DDB_G0271340	acyl-CoA oxidase	2.10	0.002
419	DDB_G0291918	Q54DY0	DDB_G0291918	putative protein serine/threonine kinase	2.10	0.000
420	DDB_G0272843	Q558S9	DDB_G0272843	unknown	2.10	0.003
421	DDB_G0280389	Q54VK8	DDB_G0280389	unknown	2.09	0.011

422	DDB_G0285383	Q54N95	DDB_G0285383	unknown	2.09	0.011
423	DDB_G0276143	Q551X9	dhkF	histidine kinase F	2.09	0.011
424	DDB_G0267716	Q55GD5	DDB_G0267716	unknown	2.09	0.001
425	DDB_G0275209	Q554F5	cnrD	ubiquitin system component Cue domain containing protein, putative cell number regulator	2.09	0.000
426	DDB_G0272676	Q86A14	sf3a1	ubiquitin domain-containing protein, splicing factor 3A subunit 1	2.09	0.000
427	DDB_G0278537	Q54XX4	beiB	cup-specific protein B	2.09	0.001
428	DDB_G0274165	Q86KD2	DDB_G0274165	unknown	2.09	0.000
429	DDB_G0280977	Q8ST87	abcC10	ABC transporter C family protein	2.09	0.000
430	DDB_G0285685	Q54MS9	DDB_G0285685	unknown	2.09	0.002
431	DDB_G0285615	Q54MX8	iliA	unknown	2.09	0.001
432	DDB_G0270996	Q55CT3	DDB_G0270996	unknown	2.08	0.000
433	DDB_G0269008	Q55EM5	DDB_G0269008	unknown	2.08	0.012
434	DDB_G0275111	Q8T2T8	DDB_G0275111	unknown	2.08	0.000
435	DDB_G0272662	Q86IL4	DDB_G0272662	putative protein tyrosine phosphatase, dual specificity	2.08	0.000
436	DDB_G0272929	Q86II1	isg12	unknown	2.08	0.000
437	DDB_G0277167	Q86AR0	DDB_G0277167	unknown	2.07	0.004
438	DDB_G0267652	N/A	N/A	N/A	2.07	0.007
439	DDB_G0272989	Q86IF6	vps5	Phox domain-containing protein, putative sorting nexin	2.07	0.000
440	DDB_G0273131	Q557D2	g6pd-1	glucose 6-phosphate-1-dehydrogenase	2.07	0.001
441	DDB_G0277849	Q94464	dymA	dynamin A	2.07	0.000
442	DDB_G0274163	Q86KD3	DDB_G0274163	unknown	2.07	0.008
443	DDB_G0277711	Q54ZA1	DDB_G0277711_RTE	TRE5-B ORF2	2.07	0.005
444	DDB_G0286895	Q54L53	grlD	GPCR family 3 protein 4	2.06	0.000
445	DDB_G0277911	Q54XZ0	mfeB	unknown	2.06	0.000
446	DDB_G0288019	Q54JJ1	DDB_G0288019	CMP/dCMP deaminase, zinc-binding domain-containing protein	2.06	0.000
447	DDB_G0272977	Q559B7	DDB_G0272977	unknown	2.06	0.000
448	DDB_G0272845	Q558U2	DDB_G0272845	AB-hydrolase associated lipase region containing protein	2.06	0.000
449	DDB_G0280921	Q54UN7	DDB_G0280921	Phox domain-containing protein	2.06	0.000
450	DDB_G0274611	N/A	DDB_G0274611_ps	pseudogene	2.06	0.013
451	DDB_G0286717	Q54LC2	ponC1	putative actin binding protein, ponticulin-related protein	2.05	0.000
452	DDB_G0276203	Q552I8	DDB_G0276203	unknown	2.05	0.000
453	DDB_G0289825	Q54GY9	DDB_G0289825	unknown	2.05	0.011
454	DDB_G0269080	Q55ES1	DDB_G0269080	unknown	2.05	0.013
455	DDB_G0271684	Q55AP1	grlA	GPCR family 3 protein 1	2.05	0.000
456	DDB_G0287685	Q54JV1	cinC	elongation factor 2, vegetative specific protein H6	2.05	0.000
457	DDB_G0279241	Q54X30	DDB_G0279241	PH domain-containing protein,<br>Arf guanyl-nucleotide exchange factor,<br>SEC7-like domain-containing protein	2.04	0.000
458	DDB_G0272680	Q86A16	DDB_G0272680	protein phosphatase 2C	2.04	0.000
459	DDB_G0272546	Q559J4	DDB_G0272546	unknown	2.04	0.005
460	DDB_G0287695	Q54K08	DDB_G0287695	FAD-binding monooxygenase	2.04	0.011
461	DDB_G0287993	Q54JK4	vps60	SNF7 family protein	2.04	0.000

462	DDB_G0276747	Q550Z2	DDB_G0276747	unknown	2.04	0.000
463	DDB_G0275735	Q552X5	rbdA	RNA-binding domain protein A	2.03	0.001
464	DDB_G0282283	Q54SV9	cyp515B1	cytochrome P450 family protein	2.03	0.001
465	DDB_G0272322	Q86KZ6	DDB_G0272322	unknown	2.03	0.000
466	DDB_G0274661	Q555E4	DDB_G0274661	unknown	2.03	0.000
467	DDB_G0286393	Q54LV6	DDB_G0286393	unknown	2.03	0.000
468	DDB_G0278297	Q54YD1	DDB_G0278297	metallophosphoesterase domain-containing protein	2.02	0.000
469	DDB_G0270540	Q55DX1	DDB_G0270540	unknown	2.02	0.001
470	DDB_G0269456	Q55DZ6	DDB_G0269456	unknown	2.02	0.015
471	DDB_G0281555	Q54TL0	kif7	kinesin family member 7	2.02	0.000
472	DDB_G0269698	Q55DD3	DDB_G0269698	unknown	2.01	0.004
473	DDB_G0285141	Q54NM4	osbJ	oxysterol binding family protein, member 10	2.01	0.000
474	DDB_G0272813	P34112	cdk1	p34-cdc2 protein, protein serine/threonine kinase, CDK family protein kinase, CMGC group, CDC2 subfamily protein kinase	2.01	0.000
475	DDB_G0274487	Q869R1	DDB_G0274487	Protein PNS1	2.01	0.000
476	DDB_G0286897	Q54L52	DDB_G0286897	unknown	2.01	0.000
477	DDB_G0284261	Q54PW0	nxnB	annexin I, putative actin binding protein	2.00	0.009
478	DDB_G0281219	Q54U83	DDB_G0281219	Nucleoside diphosphate-linked moiety X motif 6	2.00	0.000
479	DDB_G0279767	Q54WB7	DDB_G0279767	unknown	2.00	0.001
480	DDB_G0272690	Q86IG6	DDB_G0272690	unknown	2.00	0.000
481	DDB_G0273087	N/A	DDB_G0273087_ps	pseudogene	2.00	0.014
482	DDB_G0276407	Q86HD7	DDB_G0276407	adenylate kinase	2.00	0.001
483	DDB_G0272765	Q86L43	DDB_G0272765	unknown	2.00	0.000
484	DDB_G0274171	Q86KC7	stcC	unknown	2.00	0.005
485	DDB_G0272771	Q86L37	wdr7	WD40 repeat-containing protein	2.00	0.000
486	DDB_G0282061	Q54T09	DDB_G0282061	unknown	2.00	0.007
487	DDB_G0293794	Q54BA7	DDB_G0293794	unknown	2.00	0.000

Down-regulated genes in ATG16¯ versus AX2 cells

#	DDB_G ID	UniProt ID	GeneName	GeneProduct	FC	p-value
1	DDB_G0267238	Q23896	DDB_G0267238_RTE	DIRS1 ORF1	-12.95	0.000
2	DDB_G0269930	Q55CR9	DDB_G0269930	Autoinducer 2-degrading protein lsrG	-7.86	0.000
3	DDB_G0279891	Q54W68	DDB_G0279891	unknown	-6.90	0.000
4	DDB_G0291223	Q54EZ5	DDB_G0291223_RTE	DIRS1 ORF2/ORF3 fusion fragment	-6.88	0.000
5	DDB_G0270058	Q55CG8	DDB_G0270058	unknown	-6.78	0.000
6	DDB_G0279957	Q54W27	DDB_G0279957	unknown	-5.77	0.000
7	DDB_G0290285	Q54GB8	DDB_G0290285	unknown	-4.82	0.000
8	DDB_G0274377	Q86IU6	DDB_G0274377	unknown	-4.82	0.000
9	DDB_G0285917	Q54MI7	DDB_G0285917	unknown	-4.71	0.000
10	DDB_G0287665	Q54K31	DDB_G0287665	unknown	-4.68	0.000
11	DDB_G0294246	Q54AS3	DDB_G0294246_TE	Tdd-4	-4.61	0.000
12	DDB_G0280149	Q54VT3	DDB_G0280149	unknown	-4.61	0.000
13	DDB_G0285687	Q54MS8	DDB_G0285687	unknown	-4.57	0.000

14	DDB_G0275323	O15736	tipD	autophagy protein 16	-4.54	0.000
15	DDB_G0289629	Q54H83	DDB_G0289629	unknown	-4.41	0.000
16	DDB_G0288103	Q54JE1	sibB	type A von Willebrand factor (VWFA) domain-containing protein	-4.34	0.000
17	DDB_G0290349	Q54GB7	DDB_G0290349	unknown	-4.33	0.000
18	DDB_G0279953	Q54W29	DDB_G0279953	unknown	-4.18	0.000
19	DDB_G0276513	Q551I0	DDB_G0276513	unknown	-4.17	0.000
20	DDB_G0271438	Q55B42	DDB_G0271438	unknown	-4.17	0.000
21	DDB_G0267334	Q55GY8	DDB_G0267334_RTE	DIRS1 ORF1 fragment	-4.15	0.000
22	DDB_G0286247	Q54M25	ponB	putative actin binding protein, ponticulin-related protein	-4.06	0.000
23	DDB_G0275023	Q553U6	act22	actin	-4.05	0.000
24	DDB_G0269436	Q55E16	DDB_G0269436	unknown	-4.04	0.000
25	DDB_G0268148	Q55FE8	DDB_G0268148	unknown	-4.02	0.000
26	DDB_G0281535	Q54TV7	DDB_G0281535_RTE	Skipper GAG-PRO-POL	-3.90	0.000
27	DDB_G0269928	Q55CS0	DDB_G0269928	DUF3430 family protein	-3.87	0.000
28	DDB_G0267246	Q55H33	DDB_G0267246_RTE	DIRS1 ORF1	-3.80	0.000
29	DDB_G0292102	Q54DP2	fscJ	GPCR family protein, frizzled and smoothened-like sans CRD protein	-3.78	0.000
30	DDB_G0291616	Q54EC4	DDB_G0291616	unknown	-3.74	0.000
31	DDB_G0276037	Q8MNM6	DDB_G0276037	zinc-containing alcohol dehydrogenase (ADH)	-3.74	0.000
32	DDB_G0274705	Q555N6	DDB_G0274705	putative glutathione S-transferase	-3.68	0.000
33	DDB_G0275609	Q86H73	DDB_G0275609	unknown	-3.64	0.000
34	DDB_G0295799	C7G077	DDB_G0295799	unknown	-3.61	0.000
35	DDB_G0267184	Q55H61	DDB_G0267184_RTE	DIRS1 ORF2 fragment	-3.56	0.000
36	DDB_G0294324	Q54AN4	DDB_G0294324_RTE	DIRS1 ORF1 fragment	-3.40	0.000
37	DDB_G0290239	Q54GF0	DDB_G0290239	unknown	-3.38	0.000
38	DDB_G0270812	Q55BP3	DDB_G0270812	unknown	-3.37	0.000
39	DDB_G0280881	Q54UR0	DDB_G0280881	putative glutathione S-transferase	-3.36	0.000
40	DDB_G0293116	Q54C82	DDB_G0293116	unknown	-3.31	0.000
41	DDB_G0278287	N/A	iliE_ps	pseudogene	-3.26	0.000
42	DDB_G0289367	Q54HL3	agnE	argonaut-like protein	-3.25	0.000
43	DDB_G0272052	Q55AC2	DDB_G0272052	leucine-rich repeat-containing protein (LRR), putative cell surface glycoprotein	-3.13	0.000
44	DDB_G0267244	Q55H34	DDB_G0267244_RTE	DIRS1 ORF2 fragment	-2.99	0.000
45	DDB_G0286923	Q54L37	DDB_G0286923	NADH:flavin oxidoreductase/NADH oxidase domain-containing protein	-2.95	0.000
46	DDB_G0289905	Q54GV5	DDB_G0289905	unknown	-2.94	0.000
47	DDB_G0295801	C7G078	DDB_G0295801	B_lectin domain-containing protein	-2.93	0.000
48	DDB_G0283143	Q54RJ9	DDB_G0283143	unknown	-2.90	0.000
49	DDB_G0294278	Q54AQ7	DDB_G0294278	unknown	-2.87	0.000
50	DDB_G0291676	Q54EI4	DDB_G0291676	short-chain dehydrogenase/reductase (SDR) family protein, glucose/ribitol dehydrogenase family protein	-2.85	0.000
51	DDB_G0291221	Q54EZ9	DDB_G0291221_RTE	DIRS1 ORF1	-2.85	0.000
52	DDB_G0294200	Q54AU6	DDB_G0294200	unknown	-2.85	0.000
53	DDB_G0268838	Q55EL6	DDB_G0268838	unknown	-2.82	0.000
54	DDB_G0291354	Q54ET1	DDB_G0291354	unknown	-2.82	0.000
55	DDB_G0293980	Q54B30	DDB_G0293980	unknown	-2.82	0.000

56	DDB_G0288967	Q54I69	DDB_G0288967	unknown	-2.68	0.000
57	DDB_G0285025	Q54NR1	alrE	aldo-keto reductase	-2.67	0.000
58	DDB_G0290423	Q54G39	DDB_G0290423	major facilitator superfamily protein	-2.65	0.000
59	DDB_G0271624	Q55AX3	DDB_G0271624	unknown	-2.62	0.000
60	DDB_G0270950	Q55D49	DDB_G0270950_RTE	TRE5-B ORF2	-2.61	0.001
61	DDB_G0274245	Q86KL0	DDB_G0274245	unknown	-2.60	0.001
62	DDB_G0280167	Q54VS4	psiO	PA14 domain-containing protein	-2.57	0.000
63	DDB_G0272026	Q86JM1	DDB_G0272026	unknown	-2.53	0.000
64	DDB_G0293944	Q54B21	DDB_G0293944	unknown	-2.52	0.001
65	DDB_G0270654	Q55D18	DDB_G0270654	unknown	-2.48	0.001
66	DDB_G0267304	Q55H05	DDB_G0267304_RTE	DIRS1 ORF3 fragment	-2.47	0.000
67	DDB_G0294234	N/A	N/A	N/A	-2.47	0.000
68	DDB_G0269212	Q55DQ2	abcG11	ABC transporter G family protein	-2.46	0.000
69	DDB_G0288357	Q54J17	DDB_G0288357	unknown	-2.46	0.002
70	DDB_G0270974	Q55D00	DDB_G0270974	unknown	-2.45	0.000
71	DDB_G0279503	Q54WQ1	DDB_G0279503	unknown	-2.45	0.000
72	DDB_G0274755	Q556A8	gpt7	putative glycophosphotransferase, Stealth family protein	-2.44	0.000
73	DDB_G0293890	Q54B57	DDB_G0293890	unknown	-2.44	0.002
74	DDB_G0294360	Q54AL6	DDB_G0294360_RTE	Skipper RT-IN	-2.41	0.001
75	DDB_G0274161	Q86KD5	DDB_G0274161	nmrA-like family protein	-2.40	0.002
76	DDB_G0286801	Q54LI6	DDB_G0286801	unknown	-2.40	0.000
77	DDB_G0294180	Q54AV5	DDB_G0294180_RTE	DIRS1 ORF1/ORF3 fusion fragment	-2.39	0.000
78	DDB_G0294168	Q54AW1	DDB_G0294168_RTE	DIRS1 ORF1 fragment	-2.39	0.001
79	DDB_G0275551	Q86H68	DDB_G0275551	unknown	-2.39	0.002
80	DDB_G0284511	Q54PJ5	DDB_G0284511	unknown	-2.38	0.000
81	DDB_G0289283	Q7Z202	cupC	calcium up-regulated protein, ricin B lectin domain-containing protein	-2.37	0.000
82	DDB_G0277599	Q86KL9	DDB_G0277599	unknown	-2.37	0.000
83	DDB_G0289765	Q54H11	DDB_G0289765	unknown	-2.35	0.003
84	DDB_G0294248	Q54AS2	DDB_G0294248_RTE	DIRS1 ORF1 fragment	-2.35	0.000
85	DDB_G0267194	Q55H68	DDB_G0267194	unknown	-2.31	0.004
86	DDB_G0276671	Q551B6	DDB_G0276671	unknown	-2.30	0.004
87	DDB_G0294010	Q54B00	DDB_G0294010_TE	DDT-A	-2.30	0.003
88	DDB_G0282899	Q54RY4	rabV	Rab GTPase	-2.29	0.003
89	DDB_G0267258	Q55H27	DDB_G0267258_RTE	DIRS1 ORF2 fragment	-2.27	0.000
90	DDB_G0267262	Q55H25	DDB_G0267262_RTE	DIRS1 ORF2 fragment	-2.27	0.001
91	DDB_G0267188	Q55H58	DDB_G0267188_RTE	DIRS1 ORF2 fragment	-2.26	0.000
92	DDB_G0267322	Q55GZ6	DDB_G0267322_RTE	DIRS1 ORF2 fragment	-2.25	0.001
93	DDB_G0271654	Q55AT6	tgrK3	immunoglobulin E-set domain-containing protein	-2.25	0.003
94	DDB_G0292862	Q54CM0	ppt3	palmitoyl-protein thioesterase 3	-2.24	0.000
95	DDB_G0289229	Q54HT7	adcF	arrestin domain-containing protein	-2.23	0.004
96	DDB_G0267328	Q55GZ3	DDB_G0267328_RTE	DIRS1 ORF2 fragment	-2.22	0.000
97	DDB_G0295693	B0G178	DDB_G0295693	unknown	-2.22	0.002
98	DDB_G0285013	Q54NR8	DDB_G0285013	unknown	-2.21	0.000
99	DDB_G0284629	Q54PD4	DDB_G0284629	unknown	-2.21	0.000

100	DDB_G0285379	Q54N98	DDB_G0285379	unknown	-2.21	0.000
101	DDB_G0270114	Q55CC9	DDB_G0270114	unknown	-2.20	0.000
102	DDB_G0292932	Q54CG7	DDB_G0292932	unknown	-2.19	0.001
103	DDB_G0292482	Q54D49	DDB_G0292482	unknown	-2.18	0.001
104	DDB_G0293020	Q54CF2	DDB_G0293020	unknown	-2.18	0.002
105	DDB_G0282605	Q54S88	DDB_G0282605	leucine-rich repeat-containing protein (LRR), Rho GTPase domain containing protein	-2.17	0.005
106	DDB_G0271748	N/A	DDB_G0271748_ps	pseudogene	-2.15	0.002
107	DDB_G0290779	Q54FK7	rabL	GTP binding protein RARE7L, Rab GTPase	-2.14	0.000
108	DDB_G0291137	Q54F36	lyC2	lysozyme C family protein 2	-2.14	0.001
109	DDB_G0275223	Q554J1	DDB_G0275223	unknown	-2.14	0.008
110	DDB_G0291123	P34114	glpD	glycogen phosphorylase 2	-2.13	0.000
111	DDB_G0284397	Q54PP8	DDB_G0284397	unknown	-2.12	0.009
112	DDB_G0283839	Q54QG7	grlN	G-protein-coupled receptor (GPCR) family 3 protein 13	-2.12	0.000
113	DDB_G0278563	Q54XV8	lyC3	lysozyme C family protein 3	-2.11	0.008
114	DDB_G0284209	Q54Q00	DDB_G0284209	short-chain dehydrogenase/reductase (SDR) family protein	-2.11	0.000
115	DDB_G0283613	Q54QT7	cbpC	calcium-binding protein	-2.10	0.000
116	DDB_G0289101	Q54HZ9	DDB_G0289101	unknown	-2.10	0.011
117	DDB_G0294192	Q54AV0	DDB_G0294192	unknown	-2.10	0.008
118	DDB_G0289883	Q7Z1Z9	cupG	calcium up-regulated protein, ricin B lectin domain-containing protein	-2.10	0.000
119	DDB_G0294204	Q54AU4	DDB_G0294204_RTE	DIRS1 ORF1 fragment	-2.09	0.001
120	DDB_G0270092	Q55CE7	DDB_G0270092	unknown	-2.09	0.010
121	DDB_G0286685	Q54LE1	DDB_G0286685	unknown	-2.09	0.008
122	DDB_G0267332	Q55GZ0	DDB_G0267332	unknown	-2.09	0.010
123	DDB_G0273301	Q556X6	DDB_G0273301	unknown	-2.09	0.006
124	DDB_G0279337	Q54WX7	DDB_G0279337	unknown	-2.08	0.012
125	DDB_G0278001	Q54YY8	DDB_G0278001	unknown	-2.07	0.003
126	DDB_G0271028	Q55CG3	DDB_G0271028_RTE	TRE5-A ORF1	-2.06	0.003
127	DDB_G0271808	Q55AN5	DDB_G0271808	unknown	-2.06	0.001
128	DDB_G0286861	Q54L76	DDB_G0286861	unknown	-2.06	0.000
129	DDB_G0279955	Q54W28	DDB_G0279955	unknown	-2.06	0.007
130	DDB_G0292098	Q54DP4	DDB_G0292098	unknown	-2.05	0.006
131	DDB_G0290827	Q1ZXC0	DDB_G0290827	Rab GTPase domain-containing protein	-2.05	0.013
132	DDB_G0275963	Q552J5	DDB_G0275963	unknown	-2.05	0.011
133	DDB_G0267236	Q55H39	DDB_G0267236_RTE	DIRS1 ORF2 fragment	-2.05	0.000
134	DDB_G0276421	Q86HX2	DDB_G0276421	unknown	-2.04	0.002
135	DDB_G0279897	Q54W67	DDB_G0279897	unknown	-2.04	0.009
136	DDB_G0267306	Q55H04	DDB_G0267306_RTE	Skipper GAG-PRO-POL	-2.04	0.000
137	DDB_G0267300	Q55H07	DDB_G0267300_RTE	DIRS1 ORF1/ORF2 fusion fragment	-2.03	0.001
138	DDB_G0274117	Q86HQ2	abcG8	ABC transporter G family protein	-2.03	0.000
139	DDB_G0279917	Q54W19	abcD1	ABC transporter D family protein	-2.03	0.001
140	DDB_G0282039	Q54T21	DDB_G0282039	unknown	-2.02	0.003
141	DDB_G0276907	Q86JA7	DDB_G0276907	unknown	-2.02	0.003
142	DDB_G0290225	Q54GD5	DDB_G0290225	patatin family protein	-2.02	0.001

143	DDB_G0267326	Q55GZ4	DDB_G0267326_RTE	DIRS1 ORF2 fragment	-2.02	0.000
144	DDB_G0275523	Q86A00	DDB_G0275523	unknown	-2.02	0.004
145	DDB_G0284341	Q54PT6	lyrm5	LYR motif-containing protein 5	-2.02	0.000
146	DDB_G0283103	Q54RK1	DDB_G0283103	unknown	-2.02	0.010
147	DDB_G0279475	Q54WR8	gna1	glucosamine 6-phosphate N-acetyltransferase	-2.02	0.000
148	DDB_G0292894	Q54CJ8	DDB_G0292894	putative ornithine cyclodeaminase	-2.01	0.000
149	DDB_G0267198	Q55H66	DDB_G0267198	unknown	-2.01	0.012
150	DDB_G0281843	Q54TD6	DDB_G0281843	PAN-1 domain-containing protein	-2.01	0.000
151	DDB_G0267324	Q55GZ5	DDB_G0267324_RTE	DIRS1 ORF1	-2.01	0.004
152	DDB_G0267242	Q55H36	DDB_G0267242_RTE	DIRS1 ORF1 fragment	-2.00	0.006

Up-regulated genes in ATG9‾/16‾ versus AX2 cells

#	DDB_G ID	UniProt ID	GeneName	GeneProduct	FC	p-value
1	DDB_G0288573	Q54IR6	DDB_G0288573	unknown	8.98	0.000
2	DDB_G0267228	Q55H54	DDB_G0267228_TE	DDT-B	7.53	0.000
3	DDB_G0349499	Q558U0	DDB_G0349499	unknown	6.97	0.000
4	DDB_G0282919	Q54RU5	DDB_G0282919_TE	Tdd-4	6.66	0.000
5	DDB_G0267306	Q55H04	DDB_G0267306_RTE	Skipper GAG-PRO-POL	6.03	0.000
6	DDB_G0294360	Q54AL6	DDB_G0294360_RTE	Skipper RT-IN	5.25	0.000
7	DDB_G0283553	Q54QX5	DDB_G0283553	patatin family protein	4.61	0.000
8	DDB_G0269660	N/A	N/A	N/A	4.50	0.000
9	DDB_G0267250	Q55H31	DDB_G0267250_TE	DDT-B	4.47	0.000
10	DDB_G0281535	Q54TV7	DDB_G0281535_RTE	Skipper GAG-PRO-POL	4.45	0.000
11	DDB_G0269662	Q55DH1	DDB_G0269662_RTE	TRE5-B ORF2 fragment	4.21	0.000
12	DDB_G0290993	Q54F96	DDB_G0290993	unknown	3.67	0.000
13	DDB_G0283833	Q54QH1	DDB_G0283833	unknown	3.58	0.000
14	DDB_G0279995	Q54VZ5	DDB_G0279995	putative glycoside hydrolase	3.34	0.000
15	DDB_G0280011	Q54W47	DDB_G0280011_TE	transposable element	3.16	0.000
16	DDB_G0272849	Q558U5	DDB_G0272849	EGF-like domain-containing protein	3.16	0.000
17	DDB_G0279985	Q54W02	DDB_G0279985	putative transmembrane protein	3.06	0.000
18	DDB_G0276793	Q7KWW7	DDB_G0276793	unknown	3.06	0.000
19	DDB_G0293208	Q54C15	DDB_G0293208	unknown	3.05	0.000
20	DDB_G0272769	Q86L41	DDB_G0272769	unknown	3.03	0.000
21	DDB_G0277711	Q54ZA1	DDB_G0277711_RTE	TRE5-B ORF2	2.96	0.000
22	DDB_G0272785	Q86B07	DDB_G0272785	acyloxyacyl hydrolase	2.96	0.000
23	DDB_G0272688	Q86IG4	DDB_G0272688	NmrA-like protein	2.95	0.000
24	DDB_G0285585	Q54N02	DDB_G0285585	unknown	2.94	0.000
25	DDB_G0270212	Q58A40	DDB_G0270212	galactose-binding domain-containing protein	2.87	0.000
26	DDB_G0281207	Q54U91	DDB_G0281207	EGF-like domain-containing protein	2.84	0.000
27	DDB_G0272961	Q559D1	DDB_G0272961	unknown	2.78	0.000
28	DDB_G0281817	Q54TF4	DDB_G0281817	unknown	2.77	0.000
29	DDB_G0267362	Q55GX6	DDB_G0267362_RTE	Skipper GAG-PRO-POL	2.72	0.000
30	DDB_G0284729	Q54PA5	fslK	frizzled and smoothened-like protein K	2.70	0.000
31	DDB_G0284807	Q54P41	DDB_G0284807	IPT/TIG domain-containing protein, EGF-like domain-containing protein	2.69	0.000

32	DDB_G0270214	Q55C60	DD7-1	galactose-binding domain-containing protein	2.68	0.000
33	DDB_G0287035	Q9BKJ9	lmpB	lysosomal integral membrane glycoprotein LmpB	2.67	0.000
34	DDB_G0272843	Q558S9	DDB_G0272843	unknown	2.67	0.000
35	DDB_G0272827	Q966R0	cbpI	EF-hand domain-containing protein	2.67	0.000
36	DDB_G0272993	Q558Z4	DDB_G0272993	unknown	2.66	0.000
37	DDB_G0287685	Q54JV1	cinC	elongation factor 2, vegetative specific protein H6	2.63	0.000
38	DDB_G0285685	Q54MS9	DDB_G0285685	unknown	2.63	0.000
39	DDB_G0272783	Q86B08	rliA	major facilitator superfamily protein	2.62	0.000
40	DDB_G0283979	Q54QA1	DDB_G0283979	unknown	2.62	0.000
41	DDB_G0267610	Q55GM3	DDB_G0267610	NAD-dependent epimerase/dehydratase family protein	2.57	0.000
42	DDB_G0286609	Q1ZXE4	fslQ	frizzled and smoothened-like protein Q	2.54	0.000
43	DDB_G0268142	Q55FF1	DDB_G0268142	unknown	2.52	0.000
44	DDB_G0272977	Q559B7	DDB_G0272977	unknown	2.46	0.000
45	DDB_G0272955	Q559E7	DDB_G0272955	putative phytanoyl-CoA dioxygenase	2.45	0.000
46	DDB_G0306979	0	DDB_G0306979	EGF-like domain-containing protein	2.43	0.000
47	DDB_G0295669	B0G192	DDB_G0295669	isochorismatase hydrolase	2.41	0.000
48	DDB_G0289171	Q54HW8	DDB_G0289171	unknown	2.40	0.000
49	DDB_G0290655	Q54FX8	DDB_G0290655	unknown	2.39	0.000
50	DDB_G0272867	Q558Z0	argS1	arginyl-tRNA synthetase	2.38	0.000
51	DDB_G0272726	Q86IK2	DDB_G0272726	unknown	2.36	0.000
52	DDB_G0270730	Q55CD6	fslB	frizzled and smoothened-like protein B	2.35	0.000
53	DDB_G0272522	Q86KF9	sgkA	sphingosine kinase	2.33	0.000
54	DDB_G0272965	Q86IH2	DDB_G0272965	unknown	2.33	0.000
55	DDB_G0272730	Q7KWM5	alg2	alpha-1,3-mannosyltransferase	2.33	0.000
56	DDB_G0273009	Q86L39	DDB_G0273009	unknown	2.32	0.000
57	DDB_G0272767	Q86L42	DDB_G0272767	unknown	2.30	0.000
58	DDB_G0272765	Q86L43	DDB_G0272765	unknown	2.30	0.000
59	DDB_G0278975	Q54XG9	DDB_G0278975	unknown	2.28	0.000
60	DDB_G0272995	Q558Z1	DDB_G0272995	unknown	2.27	0.000
61	DDB_G0272935	Q559G8	mocos	molybdenum cofactor sulfurase	2.27	0.000
62	DDB_G0267252	Q55H30	DDB_G0267252	unknown	2.26	0.000
63	DDB_G0272837	Q558U1	ifkA	PEK family protein kinase IfkA	2.25	0.000
64	DDB_G0272829	Q86L54	DDB_G0272829	unknown	2.23	0.000
65	DDB_G0278647	Q54YE1	tps7	terpene synthase 7	2.23	0.000
66	DDB_G0268212	Q55F84	DDB_G0268212	putative DEAD/DEAH box helicase	2.21	0.000
67	DDB_G0278613	Q54YP0	DDB_G0278613	EGF-like domain-containing protein, matrilin-like protein	2.20	0.000
68	DDB_G0271042	Q55C92	DDB_G0271042	unknown	2.19	0.000
69	DDB_G0272937	Q559G7	DDB_G0272937	unknown	2.19	0.000
70	DDB_G0272668	Q86IL5	ppr2	protein phosphatase 4 regulatory SU 2	2.19	0.000
71	DDB_G0272981	Q559A9	pks13	putative polyketide synthase, beta-ketoacyl synthase family protein	2.18	0.000
72	DDB_G0281735	Q54TI1	DDB_G0281735	putative transmembrane protein	2.17	0.000
73	DDB_G0272666	Q86JC1	DDB_G0272666	putative ankyrin repeat protein	2.17	0.000
74	DDB_G0271138	Q55BC0	abcA8	ABC transporter A family protein	2.17	0.000

75	DDB_G0290511	N/A	N/A	N/A	2.15	0.000
76	DDB_G0290975	Q54FB4	DDB_G0290975	alpha/beta hydrolase fold-3 domain-containing protein	2.15	0.000
77	DDB_G0272973	Q559C0	DDB_G0272973	unknown	2.15	0.000
78	DDB_G0269538	Q55DT1	DDB_G0269538	unknown	2.14	0.000
79	DDB_G0272987	Q559A2	irlA	putative protein serine/threonine kinase, IRE family protein kinase	2.14	0.000
80	DDB_G0273383	Q557T0	cwc15-1	putative pre-mRNA-splicing factor CWC15	2.14	0.000
81	DDB_G0280977	Q8ST87	abcC10	ABC transporter C family protein	2.13	0.000
82	DDB_G0272869	Q558Z2	sun1	SUN domain-containing protein 1	2.12	0.000
83	DDB_G0272696	Q86IH5	DDB_G0272696	unknown	2.12	0.000
84	DDB_G0281345	Q54U30	DDB_G0281345	unknown	2.12	0.000
85	DDB_G0277531	Q54ZQ4	DDB_G0277531	EGF-like domain-containing protein	2.11	0.000
86	DDB_G0272771	Q86L37	wdr7	WD40 repeat-containing protein	2.11	0.000
87	DDB_G0272983	Q559A6	DDB_G0272983	DNAJ heat shock N-terminal domain-containing protein	2.11	0.000
88	DDB_G0276905	Q86JA8	DDB_G0276905	unknown	2.10	0.000
89	DDB_G0288571	Q54IR7	DDB_G0288571	unknown	2.10	0.000
90	DDB_G0271140	Q8T6J0	abcA7	ABC transporter A family protein	2.10	0.000
91	DDB_G0275659	Q869K4	DDB_G0275659	unknown	2.09	0.000
92	DDB_G0272997	Q558Y5	DDB_G0272997	unknown	2.09	0.000
93	DDB_G0276479	Q86HV8	ctnC	countin3	2.09	0.000
94	DDB_G0287609	Q54K46	DDB_G0287609	alpha/beta hydrolase fold-3 domain-containing protein	2.07	0.000
95	DDB_G0272680	Q86A16	DDB_G0272680	protein phosphatase 2C	2.07	0.000
96	DDB_G0277793	Q54Z79	DDB_G0277793	unknown	2.07	0.000
97	DDB_G0278679	Q1ZXI7	cyp513F1	cytochrome P450 family protein	2.07	0.000
98	DDB_G0272694	Q86IG9	gacH	RhoGAP domain-containing protein	2.06	0.000
99	DDB_G0272857	Q86L51	rapB	Rap GTPase RapB	2.06	0.000
100	DDB_G0272985	Q559A5	vps54	vacuolar protein sorting 54 family protein	2.06	0.000
101	DDB_G0288519	Q54IU1	DDB_G0288519	unknown	2.06	0.000
102	DDB_G0272742	Q7KWP5	DDB_G0272742	unknown	2.06	0.000
103	DDB_G0279817	Q54W93	DDB_G0279817	unknown	2.05	0.000
104	DDB_G0275179	Q553U5	DDB_G0275179	adenosine deaminase-related growth factor	2.04	0.000
105	DDB_G0272999	Q558Y3	pkd2	polycystin-2	2.04	0.000
106	DDB_G0272746	Q7KWQ0	DDB_G0272746	unknown	2.04	0.000
107	DDB_G0272682	Q86IG2	DDB_G0272682	endonuclease/exonuclease/phosphatase domain-containing protein, cry34 related protein	2.04	0.000
108	DDB_G0294254	Q54AR9	DDB_G0294254_RTE	Skipper RT-IN	2.02	0.000
109	DDB_G0273003	Q86L45	snrpD3	LSM (like-Sm) domain-containing protein, putative small nuclear ribonucleoprotein D3	2.02	0.000
110	DDB_G0272758	Q86L47	chtf18	chromosome transmission fidelity protein 18 homolog	2.01	0.000
111	DDB_G0272957	Q559E6	DDB_G0272957	Protein FRA10AC1	2.01	0.000
112	DDB_G0275915	Q552S6	DDB_G0275915	unknown	2.00	0.000
113	DDB_G0291121	P14326	cinB	esterase/lipase/thioesterase domain-containing protein, veg. specific protein H5	2.00	0.000
114	DDB_G0272760	Q86B14	mcm6	MCM family protein, putative DNA replication licensing factor	2.00	0.000

Down-regulated genes in ATG9^{-}/16^{-} versus AX2 cells

#	DDB_G ID	UniProt ID	GeneName	GeneProduct	FC	p-value
1	DDB_G0285917	Q54MI7	DDB_G0285917	unknown	-8.75	0.000
2	DDB_G0288967	Q54I69	DDB_G0288967	unknown	-6.61	0.000
3	DDB_G0269162	Q55BY0	atg13	autophagy protein 13	-5.83	0.000
4	DDB_G0289629	Q54H83	DDB_G0289629	unknown	-5.28	0.000
5	DDB_G0275323	O15736	tipD	autophagy protein 16	-4.78	0.000
6	DDB_G0293116	Q54C82	DDB_G0293116	unknown	-4.69	0.000
7	DDB_G0274755	Q556A8	gpt7	putative glycophosphotransferase, Stealth family protein	-4.59	0.000
8	DDB_G0280103	Q54VW2	DDB_G0280103	unknown	-4.45	0.000
9	DDB_G0274369	Q86IV0	DDB_G0274369	unknown	-4.37	0.000
10	DDB_G0289367	Q54HL3	agnE	argonaut-like protein	-4.23	0.000
11	DDB_G0282039	Q54T21	DDB_G0282039	unknown	-4.13	0.000
12	DDB_G0276513	Q551I0	DDB_G0276513	unknown	-4.00	0.000
13	DDB_G0286723	Q54LB9	ponC5	putative actin binding protein, ponticulin-related protein	-3.90	0.000
14	DDB_G0284999	Q54NT1	DDB_G0284999	unknown	-3.79	0.000
15	DDB_G0278219	Q54YI9	DDB_G0278219	unknown	-3.51	0.000
16	DDB_G0285687	Q54MS8	DDB_G0285687	unknown	-3.43	0.000
17	DDB_G0291738	Q54E92	rabG1	Rab GTPase	-3.41	0.000
18	DDB_G0289529	Q54HF4	DDB_G0289529	unknown	-3.32	0.000
19	DDB_G0294246	Q54AS3	DDB_G0294246_TE	Tdd-4	-3.28	0.000
20	DDB_G0289869	Q54GX4	DDB_G0289869	unknown	-3.17	0.000
21	DDB_G0270406	Q55BQ5	eIF4e3	eukaryotic translation initiation factor 4E member 3	-3.11	0.000
22	DDB_G0277853	Q54YG2	ecmA	extracellular matrix protein ST430	-3.10	0.000
23	DDB_G0286717	Q54LC2	ponC1	putative actin binding protein, ponticulin-related protein	-3.05	0.000
24	DDB_G0282745	Q54S01	DDB_G0282745	unknown	-3.04	0.000
25	DDB_G0278731	Q54XQ9	pgtD	IPT/TIG domain-containing protein, IQ calmodulin-binding domain-containing protein, putative glycosyltransferase, PKD/REJ (Polycystic Kidney Disease/ Receptor for Egg Jelly) domain-containing protein, GPS (GPCR Proteolytic Site) domain-containing protein	-3.01	0.000
26	DDB_G0271438	Q55B42	DDB_G0271438	unknown	-3.00	0.000
27	DDB_G0272240	Q75JP9	fscD	GPCR family protein, frizzled and smoothened-like sans CRD protein	-2.95	0.000
28	DDB_G0291616	Q54EC4	DDB_G0291616	unknown	-2.91	0.000
29	DDB_G0285323	Q54NA3	atg9	autophagy protein 9	-2.90	0.000
30	DDB_G0267188	Q55H58	DDB_G0267188_RTE	DIRS1 ORF2 fragment	-2.89	0.000
31	DDB_G0268454	Q55FC6	DDB_G0268454	unknown	-2.88	0.000
32	DDB_G0276597	Q86HF1	DDB_G0276597	unknown	-2.88	0.000
33	DDB_G0279957	Q54W27	DDB_G0279957	unknown	-2.87	0.000
34	DDB_G0288103	Q54JE1	sibB	type A von Willebrand factor (VWFA) domain-containing protein	-2.85	0.000
35	DDB_G0279953	Q54W29	DDB_G0279953	unknown	-2.85	0.000

36	DDB_G0286597	Q54LK5	DDB_G0286597	unknown	-2.84	0.000
37	DDB_G0278553	Q54XW3	DDB_G0278553	unknown	-2.82	0.000
38	DDB_G0280167	Q54VS4	psiO	PA14 domain-containing protein	-2.79	0.000
39	DDB_G0271150	Q55B32	DDB_G0271150	unknown	-2.79	0.000
40	DDB_G0293864	Q54B73	DDB_G0293864	unknown	-2.79	0.000
41	DDB_G0290349	Q54GB7	DDB_G0290349	unknown	-2.73	0.000
42	DDB_G0293048	Q54CC7	DDB_G0293048	unknown	-2.72	0.000
43	DDB_G0293854	Q54B78	beiD	cup-specific protein D	-2.72	0.000
44	DDB_G0290737	Q54FN2	pks34	putative polyketide synthase, beta-ketoacyl synthase family protein	-2.68	0.000
45	DDB_G0267208	Q55H49	DDB_G0267208_RTE	DIRS1 ORF1 fragment	-2.68	0.000
46	DDB_G0286721	Q54LC0	ponC3	putative actin binding protein, ponticulin-related protein	-2.64	0.000
47	DDB_G0275609	Q86H73	DDB_G0275609	unknown	-2.60	0.000
48	DDB_G0274115	Q8T685	abcG12	ABC transporter G family protein	-2.60	0.000
49	DDB_G0283839	Q54QG7	grlN	GPCR family 3 protein 13	-2.58	0.000
50	DDB_G0287665	Q54K31	DDB_G0287665	unknown	-2.56	0.000
51	DDB_G0295801	C7G078	DDB_G0295801	B_lectin domain-containing protein	-2.55	0.000
52	DDB_G0289533	Q54HE9	act27	actin	-2.55	0.000
53	DDB_G0279275	Q54X11	DDB_G0279275	unknown	-2.54	0.000
54	DDB_G0271778	Q55AJ4	cyp516B1	cytochrome P450 family protein	-2.51	0.000
55	DDB_G0271696	P18154	prtA	proteosomal alpha-subunit M3	-2.49	0.000
56	DDB_G0289487	P07829	act3	actin	-2.49	0.000
57	DDB_G0286247	Q54M25	ponB	putative actin binding protein, ponticulin-related protein	-2.48	0.000
58	DDB_G0271878	Q55AI0	DDB_G0271878	unknown	-2.48	0.000
59	DDB_G0275173	Q869W0	hbx2	homeobox transcription factor Hbx2	-2.48	0.000
60	DDB_G0280149	Q54VT3	DDB_G0280149	unknown	-2.47	0.000
61	DDB_G0289145	Q54HY0	pde7	cAMP/cGMP-stimulated cAMP/cGMP phosphodiesterase 7	-2.47	0.000
62	DDB_G0284397	Q54PP8	DDB_G0284397	unknown	-2.46	0.000
63	DDB_G0269206	Q8T673	abcG21	ABC transporter G family protein	-2.46	0.000
64	DDB_G0293456	Q54BS6	DDB_G0293456	unknown	-2.46	0.000
65	DDB_G0293762	Q54BC0	DDB_G0293762	carbohydrate-binding domain-containing protein	-2.44	0.000
66	DDB_G0269902	Q55CU2	act26	actin	-2.44	0.000
67	DDB_G0269360	Q55E80	DDB_G0269360	unknown	-2.43	0.000
68	DDB_G0270654	Q55D18	DDB_G0270654	unknown	-2.41	0.000
69	DDB_G0280543	Q54V27	lmcA	unknown	-2.41	0.000
70	DDB_G0276087	Q75JJ5	DDB_G0276087	Monoglyceride lipase	-2.41	0.000
71	DDB_G0290779	Q54FK7	rabL	GTP binding protein RARE7L, Rab GTPase	-2.40	0.000
72	DDB_G0289765	Q54H11	DDB_G0289765	unknown	-2.39	0.000
73	DDB_G0270114	Q55CC9	DDB_G0270114	unknown	-2.39	0.000
74	DDB_G0286719	Q54LC1	ponC4	putative actin binding protein, ponticulin-related protein	-2.38	0.000
75	DDB_G0289905	Q54GV5	DDB_G0289905	unknown	-2.37	0.000
76	DDB_G0271152	Q55B33	DDB_G0271152	unknown	-2.36	0.000
77	DDB_G0294248	Q54AS2	DDB_G0294248_RTE	DIRS1 ORF1 fragment	-2.33	0.000
78	DDB_G0267322	Q55GZ6	DDB_G0267322_RTE	DIRS1 ORF2 fragment	-2.32	0.000

79	DDB_G0289693	Q54H57	DDB_G0289693	unknown	-2.32	0.000
80	DDB_G0276907	Q86JA7	DDB_G0276907	unknown	-2.31	0.000
81	DDB_G0286883	Q54L59	DDB_G0286883	NUDIX hydrolase family protein, dinucleoside polyphosphate hydrolase	-2.30	0.000
82	DDB_G0267432	Q55GB1	abcG15	ABC transporter G family protein	-2.30	0.000
83	DDB_G0274377	Q86IU6	DDB_G0274377	unknown	-2.29	0.000
84	DDB_G0291221	Q54EZ9	DDB_G0291221_RTE	DIRS1 ORF1	-2.28	0.000
85	DDB_G0267258	Q55H27	DDB_G0267258_RTE	DIRS1 ORF2 fragment	-2.28	0.000
86	DDB_G0291902	Q54DZ4	DDB_G0291902	cyclin-like F-box containing protein	-2.28	0.000
87	DDB_G0279681	Q1ZXH5	DDB_G0279681	calcium-binding EF-hand domain-containing protein	-2.28	0.000
88	DDB_G0277831	Q9TX43	carD	cAMP receptor 4	-2.28	0.000
89	DDB_G0289253	Q5FBC4	tmcC	HAT repeat-containing protein	-2.26	0.000
90	DDB_G0283727	Q54QN6	DDB_G0283727	short-chain dehydrogenase/reductase (SDR) family protein	-2.25	0.000
91	DDB_G0292094	Q54DP8	DDB_G0292094	unknown	-2.25	0.000
92	DDB_G0293890	Q54B57	DDB_G0293890	unknown	-2.24	0.000
93	DDB_G0267206	Q55H44	DDB_G0267206_RTE	DIRS1 ORF2 fragment	-2.24	0.000
94	DDB_G0269026	Q55EH8	abcG23	ABC transporter G family protein	-2.24	0.000
95	DDB_G0274659	Q555D7	DDB_G0274659	unknown	-2.24	0.000
96	DDB_G0284707	Q54P86	tps6	terpene synthase 6	-2.23	0.000
97	DDB_G0279901	Q54W57	DDB_G0279901	unknown	-2.23	0.000
98	DDB_G0276351	Q8SSN4	DDB_G0276351	putative glutathione S-transferase	-2.23	0.000
99	DDB_G0275023	Q553U6	act22	actin	-2.22	0.000
100	DDB_G0267178	Q55H43	DDB_G0267178_RTE	DIRS1 ORF2 fragment	-2.22	0.000
101	DDB_G0294220	Q54AT6	DDB_G0294220	unknown	-2.22	0.000
102	DDB_G0272160	Q75JU3	DDB_G0272160	unknown	-2.19	0.000
103	DDB_G0277489	Q76NU7	DDB_G0277489	unknown	-2.19	0.000
104	DDB_G0267262	Q55H25	DDB_G0267262_RTE	DIRS1 ORF2 fragment	-2.18	0.000
105	DDB_G0274117	Q86HQ2	abcG8	ABC transporter G family protein	-2.18	0.000
106	DDB_G0282135	Q54SZ3	DDB_G0282135	PhoPQ-activated pathogenicity-related protein	-2.18	0.000
107	DDB_G0283243	Q54RF7	DDB_G0283243	unknown	-2.18	0.000
108	DDB_G0270000	Q55CM4	DDB_G0270000	unknown	-2.18	0.000
109	DDB_G0284535	Q1ZXF5	cyp508A4	cytochrome P450 family protein	-2.17	0.000
110	DDB_G0271654	Q55AT6	tgrK3	immunoglobulin E-set domain-containing protein	-2.15	0.000
111	DDB_G0270038	Q55CJ1	DDB_G0270038	unknown	-2.15	0.000
112	DDB_G0276125	Q75JG1	DDB_G0276125	unknown	-2.15	0.000
113	DDB_G0271074	Q55BW8	DDB_G0271074	unknown	-2.15	0.000
114	DDB_G0280709	Q54UZ5	DDB_G0280709	unknown	-2.15	0.000
115	DDB_G0278267	Q54YF3	DDB_G0278267	PH domain-containing protein,<br>SH3 domain-containing protein,<br>Arf GTPase activating protein	-2.15	0.000
116	DDB_G0280547	Q54V32	comH	putative GATA-binding transcription factor, GATA zinc finger domain-containing protein 2	-2.13	0.000
117	DDB_G0283153	Q54RF4	cbpD1	calcium-binding protein	-2.12	0.000
118	DDB_G0270058	Q55CG8	DDB_G0270058	unknown	-2.11	0.000
119	DDB_G0290225	Q54GD5	DDB_G0290225	patatin family protein	-2.11	0.000

120	DDB_G0289283	Q7Z202	cupC	calcium up-regulated protein, ricin B lectin domain-containing protein	-2.10	0.000
121	DDB_G0288865	Q54IG0	DDB_G0288865	Uncharacterized N-acetyltransferase yjgM	-2.10	0.000
122	DDB_G0283559	Q54QW9	DDB_G0283559	unknown	-2.10	0.000
123	DDB_G0274245	Q86KL0	DDB_G0274245	unknown	-2.09	0.000
124	DDB_G0276151	Q75JJ9	DDB_G0276151	enoyl-CoA hydratase/isomerase family protein	-2.09	0.000
125	DDB_G0290285	Q54GB8	DDB_G0290285	unknown	-2.07	0.000
126	DDB_G0279455	Q54WT2	DDB_G0279455	unknown	-2.07	0.000
127	DDB_G0276215	Q552D2	DDB_G0276215	glycoside hydrolase family 15 protein	-2.07	0.000
128	DDB_G0268948	Q55EX9	DDB_G0268948	putative SAM dependent methyltransferase	-2.06	0.000
129	DDB_G0279727	Q54WH1	DDB_G0279727	unknown	-2.06	0.000
130	DDB_G0267326	Q55GZ4	DDB_G0267326_RTE	DIRS1 ORF2 fragment	-2.05	0.000
131	DDB_G0267456	P54653	cbp2	calcium-binding protein 2	-2.05	0.000
132	DDB_G0294200	Q54AU6	DDB_G0294200	unknown	-2.04	0.000
133	DDB_G0294168	Q54AW1	DDB_G0294168_RTE	DIRS1 ORF1 fragment	-2.04	0.000
134	DDB_G0272396	Q559Z3	DDB_G0272396	putative transmembrane protein	-2.04	0.000
135	DDB_G0272146	Q86JL4	DDB_G0272146	unknown	-2.04	0.000
136	DDB_G0268000	Q55FQ4	psiB	PA14 domain-containing protein	-2.03	0.000
137	DDB_G0269258	Q55EF3	DDB_G0269258_RTE	TRE3-C ORF2	-2.03	0.000
138	DDB_G0279103	Q54XA0	DDB_G0279103	unknown	-2.03	0.000
139	DDB_G0285667	Q54MT8	DDB_G0285667	unknown	-2.02	0.000
140	DDB_G0277871	Q54Y53	rsc12	unknown	-2.02	0.000
141	DDB_G0270606	Q55DF7	DDB_G0270606	unknown	-2.02	0.001
142	DDB_G0276817	Q7KWL4	DDB_G0276817	unknown	-2.02	0.000
143	DDB_G0267328	Q55GZ3	DDB_G0267328_RTE	DIRS1 ORF2 fragment	-2.01	0.000
144	DDB_G0272242	Q75JP7	cupI	ricin B lectin domain-containing protein, cup family protein	-2.01	0.000
145	DDB_G0271970	Q86AH5	DDB_G0271970	unknown	-2.01	0.000
146	DDB_G0286595	Q54LK6	DDB_G0286595	unknown	-2.00	0.001

Table S2. Common differentially regulated genes of ATG9¯, ATG16¯, and ATG9¯/16¯ cells. An absolute fold change of ≥ 2.0 and a p-value ≤ 0.05 was used for analysis. #, number.

	Strain	# of genes	Common with ATG9¯		Common with ATG16¯		Common with ATG9¯/16¯		Common with the other two strains
			#	%	#	%	#	%	%
**UP**	ATG9¯	279	-	-	209	75	40	14	12
	ATG16¯	487	209	43	-	-	54	11	7
	ATG9¯/16¯	114	40	35	54	47	-	-	29
**DOWN**	ATG9¯	53	-	-	29	55	25	47	28
	ATG16¯	152	29	19	-	-	51	34	10
	ATG9¯/16¯	146	25	17	51	35	-	-	10

Table S3. Gene Ontology (GO) statistical overrepresentation test results for DEGs of ATG9¯, ATG16¯, and ATG9¯16¯ cells using biological process, cellular component and molecular function annotation sets. GO analysis was performed with PANTHER 15.0 (released 2020-02-14). Up- and down-regulated genes with a p-value ≤ 0.05 and an absolute fold change ≥ 2.0 were used.

Up-regulated genes

Biological Process

ATG16¯		# Dictyostelium	# Experiment	expected	Fold Enrichment	raw P value	FDR
**category**	pyrimidine ribonucleoside metabolic process	8	5	0.3	16.4	6.7E-05	2.1E-02

Molecular Function

None

Cellular Component

ATG16¯		# Dictyostelium	# Experiment	expected	Fold Enrichment	raw P value	FDR
**category**	membrane	3101	156	118.4	1.3	1.7E-04	1.6E-02

Down-regulated genes

Biological Process

ATG16¯		# Dictyostelium	# Experiment	expected	Fold Enrichment	raw P value	FDR
**category**	DNA integration	49	9	0.6	15.5	2.2E-08	4.8E-05
**category**	sexual reproduction	139	9	1.7	5.5	6.0E-05	2.1E-02
**ATG9¯/16¯**							
**category**	DNA integration	49	7	0.6	12.5	3.0E-06	6.8E-03
**category**	sexual reproduction	139	9	1.6	5.7	4.7E-05	2.6E-02

Molecular Function

None

Cellular Component

ATG9¯/16¯		# Dictyostelium	# Experiment	expected	Fold Enrichment	raw P value	FDR
**category**	anchored component of membrane	23	5	0.3	19.0	1.4E-05	2.8E-03

Table S4. List of detected peptides in TMT proteome analysis. Sample preparation, protein digestion, and TMT labeling was performed as described in Materials and Methods.

Table S5. List of identified proteins in TMT proteome analysis. Based on the unique peptides from table S4 protein groups with a FDR ≤ 0.01 and at least two unique peptides were identified.

Table S6. List of DEPs in ATG9¯, ATG16¯, and ATG9¯/16¯ cells in comparison to AX2. Enriched proteins in mutant strains versus AX2 were determined by TMT proteomics and fold changes (FC) and p values of three biological replicates were calculated. For each strain comparison only those proteins with a p-value ≤ 0.05 and an absolute FC ≥ 1.2 are depicted. #, number; N/A, not available.

Up-regulated proteins in ATG9¯ versus AX2 cells

#	UniProt ID	DDB_G ID	GeneName	GeneProduct	FC	p-value
1	Q86AA1	DDB_G0274291	DDB_G0274291	Probable T4-type lysozyme 2	2.16	0.008
2	Q86ID4	DDB_G0275487	DDB0167163	Uncharacterized protein	2.11	0.017
3	Q54NT1	DDB_G0284999	DDB0186311	Uncharacterized protein	1.82	0.000
4	Q75JI6	DDB_G0276097	DDB0169498	Uncharacterized protein	1.77	0.001
5	Q55BB3	DDB_G0271254	DDB0202810	Uncharacterized protein	1.75	0.000
6	Q54C11	DDB_G0293202	DDB_G0293202	TNF receptor-associated factor family protein	1.72	0.004
7	Q8T849	DDB_G0275177	prkA	Uncharacterized protein	1.70	0.000
8	Q54DL7	DDB_G0292188	DDB_G0292188	von Willebrand factor A domain-containing protein	1.68	0.000
9	Q55GU8	DDB_G0267468	adprt4	Poly [ADP-ribose] polymerase	1.66	0.014
10	Q552D6	DDB_G0276219	DDB0203518	Uncharacterized protein	1.60	0.047
11	Q555E4	DDB_G0274661	DDB_G0274661	Uncharacterized protein	1.57	0.012
12	Q54QE9	DDB_G0283911	DDB0185738	Uncharacterized protein	1.54	0.011
13	P20610	DDB_G0277839	cxgS	Cytochrome c oxidase subunit 7s	1.51	0.001
14	Q76NV4	DDB_G0277415	DDB0169220	Uncharacterized protein	1.50	0.000
15	Q54G64	DDB_G0290377	agnB	Argonaut-like protein	1.49	0.000
16	Q54TQ4	DDB_G0281617	DDB0204563	Uncharacterized protein	1.47	0.000
17	Q54PU9	DDB_G0284277	DDB0185943	Uncharacterized protein	1.46	0.023
18	Q552L7	DDB_G0275981	DDB0203550	Uncharacterized protein	1.46	0.000
19	Q54P57	DDB_G0284779	DDB0186189	Uncharacterized protein	1.45	0.021
20	Q54H71	DDB_G0289675	adprh	[Protein ADP-ribosylarginine] hydrolase	1.44	0.000
21	Q54CW8	DDB_G0292652	DDB0184503	Uncharacterized protein	1.44	0.001
22	Q54H65	DDB_G0289681	DDB0188516	Uncharacterized protein	1.44	0.020
23	Q54H60	DDB_G0289787	DDB0216125	Uncharacterized protein	1.43	0.018
24	Q55FU7	DDB_G0267942	DDB0189665	VASt domain-containing protein	1.42	0.015
25	Q55F86	DDB_G0268208	DDB0189867	Uncharacterized protein	1.42	0.027
26	Q54FB4	DDB_G0290975	DDB0189182	Abhydrolase_3 domain-containing protein	1.39	0.001
27	Q8MP79	DDB_G0277425	DDB_G0277425	Uncharacterized protein	1.39	0.038
28	Q54XS8	DDB_G0278771	DDB0206180	PHD domain-containing protein	1.39	0.001
29	O97113	DDB_G0275439	cad2	Putative calcium-dependent cell adhesion molecule 2	1.36	0.001
30	Q55CR9	DDB_G0269930	DDB0190679	ABM domain-containing protein	1.36	0.013
31	Q54RB1	DDB_G0283281	DDB0185428	SGL domain-containing protein	1.36	0.016
32	Q55DY0	DDB_G0269482	DDB0190293	VWFA domain-containing protein	1.35	0.000
33	Q54H89	DDB_G0289641	DDB0219472	B box-type domain-containing protein	1.35	0.021
34	Q6B9X6	DDB_G0268144	vwkA	Alpha-protein kinase vwkA	1.34	0.000
35	Q55AS0	N/A	DDB0203605	Uncharacterized protein	1.34	0.001
36	Q55FN4	DDB_G0268026	DDB0189725	Uncharacterized protein	1.33	0.004
37	Q54X05	DDB_G0279397	DDB0218164	SAC domain-containing protein	1.32	0.006
38	Q54M11	DDB_G0286271	DDB0186894	Uncharacterized protein	1.32	0.003
39	Q54Z19	DDB_G0277961	DDB0204326	Uncharacterized protein	1.31	0.000
40	P14330	DDB_G0280533	lmcB	Vegetative-specific protein V4	1.31	0.002
41	Q54U98	DDB_G0281199	DDB0204075	Uncharacterized protein	1.31	0.000
42	Q55GA0	DDB_G0267754	DDB_G0267754	TNF receptor-associated factor family protein	1.30	0.001
43	Q55GN4	DDB_G0267592	DDB0189392	Amino_oxidase domain-containing protein	1.30	0.001
44	Q54JL5	DDB_G0287973	DDB0187714	Uncharacterized protein	1.29	0.005
45	Q54WD8	DDB_G0279717	DDB0206002	Carboxylic ester hydrolase	1.29	0.002
46	Q86I43	DDB_G0275161	DDB_G0275161	Uncharacterized protein	1.29	0.002

47	Q86AC9	DDB_G0276361	DDB0167013	Uncharacterized protein	1.29	0.000
48	Q55B20	DDB_G0271470	DDB0216844	N-acetyltransferase domain-containing protein	1.29	0.010
49	P42529	DDB_G0276759	cbpA	Calcium-binding protein A	1.29	0.011
50	Q8MQU6	DDB_G0267426	cshA	Citrate synthase, peroxisomal	1.28	0.000
51	Q54EJ5	DDB_G0291482	gloB2	Glyoxylase B2	1.27	0.000
52	Q54WL1	DDB_G0279571	DDB0205855	Uncharacterized protein	1.27	0.014
53	Q54NR1	DDB_G0285025	alrE	Aldo-keto reductase	1.26	0.000
54	Q54QU8	DDB_G0283629	DDB_G0283629	Probable zinc transporter protein	1.26	0.009
55	Q54CW9	DDB_G0292650	DDB0184502	Uncharacterized protein	1.26	0.004
56	Q54DM8	DDB_G0292120	cnrK	RBR-type E3 ubiquitin transferase	1.25	0.000
57	Q54PP0	DDB_G0284409	DDB0185999	Fe2OG dioxygenase domain-containing protein	1.25	0.044
58	Q552B4	DDB_G0276179	DDB0203492	Uncharacterized protein	1.25	0.032
59	Q54D21	DDB_G0292574	DDB0184451	Uncharacterized protein	1.24	0.004
60	Q54Y60	DDB_G0278401	DDB_G0278401	Uncharacterized protein	1.24	0.001
61	Q54WZ2	DDB_G0279191	vacB	Vacuolin-B	1.24	0.000
62	Q54EU1	DDB_G0291338	DDB0183828	Uncharacterized protein	1.23	0.037
63	Q54QS9	DDB_G0283655	DDB0185607	Uncharacterized protein	1.23	0.001
64	Q54WE3	DDB_G0279707	DDB0205997	Uncharacterized protein	1.23	0.007
65	Q54GB3	DDB_G0290363	sybB	Synaptobrevin-B	1.22	0.049
66	Q559N8	DDB_G0272466	DDB0217020	Uncharacterized protein	1.22	0.012
67	Q54RK8	DDB_G0283097	DDB0185353	B box-type domain-containing protein	1.22	0.001
68	Q552P4	DDB_G0275937	DDB_G0275937	Uncharacterized protein	1.22	0.007
69	Q55G88	DDB_G0268522	psmG3	Proteasome assembly chaperone 3	1.21	0.041
70	Q8STF9	DDB_G0269202	Dd-gdcA	Dd-gdcA protein	1.21	0.009
71	Q54U83	DDB_G0281219	DDB0204089	Nudix hydrolase domain-containing protein	1.21	0.002
72	Q552S0	DDB_G0275711	nhe1	Sodium/hydrogen exchanger 1	1.21	0.010
73	Q54H05	DDB_G0289661	kinY	Probable serine/threonine-protein kinase kinY	1.21	0.001
74	P14326	DDB_G0291121	cinB	Vegetative-specific protein H5	1.21	0.029
75	Q55DU3	DDB_G0270884	abnA	Actobindin-A	1.21	0.001
76	P19198	DDB_G0272560	capA-1	cAMP-binding protein 1	1.21	0.007
77	Q6IMN8	DDB_G0293850	alrA	Aldose reductase A	1.21	0.000
78	Q9BIW4	DDB_G0287507	limD	LIM domain-containing protein D	1.20	0.027
79	Q86I40	DDB_G0275013	omt4	O-methyltransferase 4	1.20	0.034
80	Q553W9	DDB_G0275299	cln5	Ceroid-lipofuscinosis neuronal protein 5 homolog	1.20	0.026
81	B0G176	DDB_G0295665	DDB_G0295665	Uncharacterized protein	1.20	0.015
82	Q553N3	DDB_G0275749	DDB0202480	Uncharacterized protein	1.20	0.010
83	Q75JW5	DDB_G0272182	DDB0168738	Uncharacterized protein	1.20	0.002

Down-regulated proteins in ATG9¯ versus AX2 cells

#	UniProt ID	DDB_G ID	GeneName	GeneProduct	FC	p-value
1	Q54G81	DDB_G0290325	DDB0188839	Peptidase C50 domain-containing protein	0.51	0.003
2	P54657	DDB_G0285793	cadA	Calcium-dependent cell adhesion molecule 1	0.65	0.000
3	Q54BS2	DDB_G0293460	DDB0191955	Purple acid phosphatase	0.67	0.005
4	Q95US4	DDB_G0279921	gp130	Lipid-anchored plasma membrane glycoprotein 130	0.68	0.000
5	Q86KU5	DDB_G0277729	DDB_G0277729	Uncharacterized protein	0.71	0.000
6	P02886	DDB_G0273063	dscA-1	Discoidin-1 subunit A	0.71	0.002
7	Q54X38	DDB_G0279229	DDB0205663	Uncharacterized protein	0.71	0.001
8	Q54QT0	DDB_G0283653	DDB0185606	Uncharacterized protein	0.72	0.011
9	Q54YR8	DDB_G0278115	DDB0204431	Uncharacterized protein	0.72	0.001
10	Q54SB4	DDB_G0282559	DDB0204837	Purple acid phosphatase	0.73	0.006
11	Q54FF6	DDB_G0290887	DDB0189136	Uncharacterized protein	0.74	0.014
12	Q54PL9	DDB_G0284547	DDB0218606	Uncharacterized protein	0.75	0.000
13	Q54FF8	DDB_G0290885	DDB0189134	Uncharacterized protein	0.75	0.029
14	Q86JM5	DDB_G0272012	DDB_G0272012	Putative elongation of fatty acids protein	0.75	0.021

15	Q54PD3	DDB_G0284631	DDB_G0284631	Uncharacterized protein	0.75	0.000
16	Q556W2	DDB_G0273781	abcG17-1	ABC transporter G family member 17	0.76	0.000
17	Q55CJ0	DDB_G0270040	DDB0190759	Uncharacterized protein	0.76	0.038
18	Q54G31	DDB_G0290465	DDB0219564	Uncharacterized protein	0.76	0.000
19	Q551Y7	DDB_G0276251	DDB0217768	Uncharacterized protein	0.78	0.002
20	Q55BY4	DDB_G0271066	DDB0216761	Elongation of fatty acids protein	0.79	0.038
21	Q55AX0	DDB_G0271628	dlpC	Dynamin-like protein C	0.79	0.021
22	Q55E04	DDB_G0269448	DDB0190271	Uncharacterized protein	0.79	0.004
23	Q54UY3	DDB_G0280725	DDB0206158	Uncharacterized protein	0.79	0.043
24	O77257	DDB_G0278725	p17	Secreted protein A	0.80	0.004
25	Q54TC9	DDB_G0281821	sre1	Elongation of fatty acids protein sre1	0.80	0.002
26	Q55ED4	DDB_G0269284	DDB_G0269284	NKAP family protein	0.81	0.013
27	Q55G75	DDB_G0267786	DDB_G0267786	PH domain-containing protein	0.81	0.000
28	Q54GQ2	DDB_G0290001	DDB0188678	Methyltransf_11 domain-containing protein	0.81	0.000
29	Q54RI2	DDB_G0283127	DDB0185376	Uncharacterized protein	0.81	0.012
30	Q55FT1	DDB_G0267966	pyd1	Dihydropyrimidine dehydrogenase [NADP(+)]	0.81	0.000
31	Q54SY2	DDB_G0282181	nvl	Putative ribosome biogenesis ATPase nvl	0.82	0.011
32	Q75JV7	DDB_G0272170	gnt8	Putative beta-1,4-N-acetylglucosaminyltransferase	0.82	0.023
33	Q54IE5	DDB_G0288807	DDB0188120	Uncharacterized protein	0.82	0.013
34	Q54PD4	DDB_G0284629	DDB_G0284629	SnoaL-like domain-containing protein	0.82	0.000
35	Q54I44	DDB_G0289019	DDB0188214	Uncharacterized protein	0.82	0.040
36	P54661	DDB_G0287587	smlA	Small aggregate formation protein	0.82	0.000
37	Q556M8	DDB_G0272572	DDB0168017	Uncharacterized protein	0.83	0.007
38	B0G0Y7	DDB_G0268646	DDB_G0268646	Uncharacterized protein	0.83	0.013
39	Q54VG2	DDB_G0280349	DDB0206534	Uncharacterized protein	0.83	0.001
40	P15808	DDB_G0280045	thyA	Flavin-dependent thymidylate synthase	0.83	0.000
41	Q54YA0	DDB_G0278345	acly	Probable ATP-citrate synthase	0.83	0.000

Up-regulated proteins in ATG16‾ versus AX2 cells

#	UniProt ID	DDB_G ID	GeneName	GeneProduct	FC	p-value
1	Q55F86	DDB_G0268208	DDB0189867	Uncharacterized protein	3.19	0.003
2	Q55GC4	DDB_G0267728	DDB0189497	Uncharacterized protein	3.09	0.000
3	Q54LV6	DDB_G0286393	DDB_G0286393	Uncharacterized protein	2.63	0.002
4	Q55GC5	DDB_G0267726	DDB0189496	Uncharacterized protein	2.37	0.000
5	Q966R0	DDB_G0272827	cbpI	Calcium-binding protein I	2.34	0.003
6	Q54G11	DDB_G0290491	DDB_G0290491	Autophagy-related protein 8-like protein	2.17	0.004
7	Q55F84	DDB_G0268212	DDB0189869	Helicase ATP-binding domain-containing protein	2.07	0.000
8	Q54PK5	DDB_G0284497	DDB0186035	Uncharacterized protein	2.04	0.047
9	Q86I30	DDB_G0275083	DDB0167433	Uncharacterized protein	2.00	0.003
10	P11872	DDB_G0271666	prtB	cAMP-regulated M3R protein	1.88	0.000
11	Q86AA1	DDB_G0274291	DDB_G0274291	Probable T4-type lysozyme 2	1.88	0.012
12	Q555C4	DDB_G0274655	DDB0203205	Uncharacterized protein	1.88	0.001
13	Q54IU1	DDB_G0288519	DDB0231477	Uncharacterized protein	1.83	0.000
14	Q54FB4	DDB_G0290975	DDB0189182	Abhydrolase_3 domain-containing protein	1.83	0.000
15	Q86A17	DDB_G0272684	qdpr	Dihydropteridine reductase	1.81	0.000
16	Q54Z19	DDB_G0277961	DDB0204326	Uncharacterized protein	1.80	0.000
17	Q556H9	DDB_G0274049	DDB0202984	Uncharacterized protein	1.78	0.001
18	Q552L7	DDB_G0275981	DDB0203550	Uncharacterized protein	1.67	0.000
19	Q54CQ7	DDB_G0292738	DDB0184536	NmrA domain-containing protein	1.65	0.000
20	Q54HW8	DDB_G0289171	DDB_G0289171	Uncharacterized protein	1.64	0.000
21	Q54RN9	DDB_G0283007	DDB0185319	Uncharacterized protein	1.64	0.002
22	Q54M11	DDB_G0286271	DDB0186894	Uncharacterized protein	1.63	0.000
23	Q54RZ4	DDB_G0282815	orfSGP	BB_PF domain-containing protein	1.61	0.007

24	P14326	DDB_G0291121	cinB	Vegetative-specific protein H5	1.60	0.003
25	Q54IQ3	DDB_G0288591	DDB0188011	Uncharacterized protein	1.60	0.000
26	Q54XL5	DDB_G0278865	DDB0206248	Uncharacterized protein	1.59	0.002
27	Q8T137	DDB_G0272754	gsr	Glutathione reductase	1.59	0.000
28	Q556N3	DDB_G0272576	DDB0168021	Uncharacterized protein	1.57	0.029
29	Q86L41	DDB_G0272769	DDB0168967	Acyl_transf_3 domain-containing protein	1.57	0.006
30	Q551L7	DDB_G0276551	DDB0217807	G domain-containing protein	1.56	0.017
31	Q55DZ2	DDB_G0269462	DDB_G0269462	Ubiquitin domain-containing protein	1.55	0.000
32	Q556R9	DDB_G0273177	DDB_G0273871	UPF0734 protein DDB_G0273871/DDB_G0273177	1.54	0.000
33	Q54VH2	DDB_G0280333	DDB0206524	Uncharacterized protein	1.54	0.000
34	Q54NT1	DDB_G0284999	DDB0186311	Uncharacterized protein	1.54	0.000
35	Q552B4	DDB_G0276179	DDB0203492	Uncharacterized protein	1.54	0.047
36	Q552P4	DDB_G0275937	DDB_G0275937	Uncharacterized protein	1.53	0.018
37	Q558Z0	DDB_G0272867	argS1	Probable arginine--tRNA ligase, cytoplasmic	1.53	0.000
38	Q54Y60	DDB_G0278401	DDB_G0278401	Uncharacterized protein	1.52	0.000
39	Q54C11	DDB_G0293202	DDB_G0293202	TNF receptor-associated factor family protein	1.51	0.002
40	Q54RB1	DDB_G0283281	DDB0185428	SGL domain-containing protein	1.48	0.015
41	Q54QE9	DDB_G0283911	DDB0185738	Uncharacterized protein	1.47	0.010
42	Q86IC9	DDB_G0275499	omt5	Probable caffeoyl-CoA O-methyltransferase 1	1.45	0.002
43	Q54QS9	DDB_G0283655	DDB0185607	Uncharacterized protein	1.45	0.001
44	Q556Y3	DDB_G0273739	DDB0168100	SMP-LTD domain-containing protein	1.45	0.002
45	Q55F82	DDB_G0268600	DDB_G0268600	Uncharacterized protein	1.45	0.015
46	Q54JX4	DDB_G0287879	DDB0219270	Uncharacterized protein	1.45	0.003
47	Q553N3	DDB_G0275749	DDB0202480	Uncharacterized protein	1.44	0.006
48	Q556K0	DDB_G0273999	DDB0167985	RWD domain-containing protein	1.44	0.000
49	Q1ZXM2	DDB_G0273013	uglB	Uracil-DNA glycosylase	1.44	0.000
50	Q54GD8	DDB_G0290223	DDB_G0290223	PXMP2/4 family protein 3	1.44	0.003
51	Q54ML6	DDB_G0285871	mog1	Probable ran guanine nucleotide release factor	1.44	0.000
52	Q55DW0	DDB_G0269508	DDB0190312	Uncharacterized protein	1.43	0.001
53	Q557E6	DDB_G0273139	DDB0168161	DNA repair protein RAD51 homolog	1.42	0.002
54	Q556M4	DDB_G0272604	cyp508A2-1	Probable cytochrome P450 508A2	1.42	0.000
55	Q55CE3	DDB_G0270098	DDB0190801	Uncharacterized protein	1.42	0.000
56	Q54NR1	DDB_G0285025	alrE	Aldo-keto reductase	1.42	0.000
57	Q555E4	DDB_G0274661	DDB_G0274661	Uncharacterized protein	1.41	0.012
58	Q7KWQ0	DDB_G0272746	DDB0168237	Uncharacterized protein	1.41	0.001
59	Q95ZG5	DDB_G0273051	DrnA	Putative RNase III	1.41	0.001
60	Q556G3	DDB_G0272632	gsta2-1	Putative glutathione S-transferase alpha-2	1.41	0.000
61	Q1ZXL7	DDB_G0273047	cyp508A3-1	Probable cytochrome P450 508A3	1.41	0.000
62	Q54BI7	DDB_G0293604	DDB0192039	Uncharacterized protein	1.41	0.000
63	Q54H65	DDB_G0289681	DDB0188516	Uncharacterized protein	1.40	0.042
64	Q54MG1	DDB_G0285981	DDB_G0285981	von Willebrand factor A domain-containing protein	1.39	0.000
65	Q54U83	DDB_G0281219	DDB0204089	Nudix hydrolase domain-containing protein	1.39	0.010
66	Q557F3	DDB_G0273597	DDB0168168	Uncharacterized protein	1.38	0.004
67	Q54DI2	DDB_G0292236	DDB0184285	Uncharacterized protein	1.38	0.007
68	Q54JL5	DDB_G0287973	DDB0187714	Uncharacterized protein	1.38	0.007
69	Q7KWM9	DDB_G0272738	iunH	Probable ribonucleoside hydrolase	1.38	0.000
70	P54670	DDB_G0277827	cafA	Calfumirin-1	1.38	0.000
71	Q557H1	DDB_G0273471	dpp3-1	Dipeptidyl peptidase 3	1.38	0.000
72	Q54WL1	DDB_G0279571	DDB0205855	Uncharacterized protein	1.38	0.006
73	P14330	DDB_G0280533	lmcB	Vegetative-specific protein V4	1.38	0.000
74	Q559T9	DDB_G0272280	DDB_G0272280	AhpC/TSA family protein	1.37	0.001
75	Q6IMN8	DDB_G0293850	alrA	Aldose reductase A	1.37	0.000
76	Q557H6	DDB_G0273555	DDB_G0273411	EFP_N domain-containing protein	1.37	0.000
77	P19198	DDB_G0272560	capA-1	cAMP-binding protein 1	1.36	0.002
78	Q55G11	DDB_G0268374	dcd2B	Neutral ceramidase B	1.36	0.016
79	Q94497	DDB_G0282367	selD	Selenide, water dikinase	1.36	0.034

80	Q54KP3	DDB_G0287219	DDB_G0287219	Uncharacterized protein	1.36	0.002
81	Q554F5	DDB_G0275209	cnrD	Putative countin receptor Cnr4	1.35	0.001
82	Q54WE3	DDB_G0279707	DDB0205997	Uncharacterized protein	1.35	0.002
83	Q7KWQ2	DDB_G0272660	serS	Serine--tRNA ligase, cytoplasmic	1.34	0.000
84	Q86KF9	DDB_G0272522	sgkA	Sphingosine kinase A	1.34	0.000
85	Q55EX9	DDB_G0268948	DDB_G0268948	Putative methyltransferase	1.34	0.000
86	Q54L14	DDB_G0286977	DDB0218872	J domain-containing protein	1.33	0.003
87	Q556J9	DDB_G0272889	surf1-1	SURF1-like protein	1.33	0.015
88	Q54DY1	DDB_G0291916	argJ	Arginine biosynthesis bifunctional protein ArgJ, mitochondrial	1.33	0.000
89	Q75JI6	DDB_G0276097	DDB0169498	Uncharacterized protein	1.33	0.002
90	Q54LY4	DDB_G0286305	DDB0186919	PKS_ER domain-containing protein	1.33	0.000
91	Q55GM3	DDB_G0267610	DDB0189402	Epimerase domain-containing protein	1.33	0.001
92	Q552M1	DDB_G0275989	DDB0203554	PKS_AT domain-containing protein	1.33	0.022
93	Q556U6	DDB_G0273093	bip1-1	Luminal-binding protein 1	1.32	0.000
94	Q54GN9	DDB_G0290029	DDB0220663	C2H2 type Zn-finger-containing protein	1.32	0.000
95	Q54YN2	DDB_G0278155	mai	Maleylacetoacetate isomerase	1.32	0.000
96	Q556K7	DDB_G0272801	DDB0167992	Uncharacterized protein	1.32	0.001
97	Q86IL5	DDB_G0272668	ppp4r2	Serine/threonine-protein phosphatase 4 regulatory subunit 2	1.32	0.000
98	Q55FS7	DDB_G0268554	DDB0216570	Uncharacterized protein	1.32	0.018
99	Q54DM8	DDB_G0292120	cnrK	RBR-type E3 ubiquitin transferase	1.32	0.000
100	Q54SH3	DDB_G0282467	DDB0204777	Aminotran_1_2 domain-containing protein	1.31	0.001
101	Q75K22	DDB_G0275469	DDB0167125	Uncharacterized protein	1.31	0.000
102	Q556W8	DDB_G0273297	DDB0203045	Uncharacterized protein	1.31	0.006
103	Q557C7	DDB_G0273247	DDB0168140	Uncharacterized protein	1.31	0.000
104	Q54F16	DDB_G0291191	DDB0219654	Uncharacterized protein	1.31	0.000
105	Q94503	DDB_G0279185	cprF	Cysteine proteinase 6	1.30	0.001
106	Q86B10	DDB_G0272777	DDB0168975	Uncharacterized protein	1.30	0.022
107	Q55CL7	DDB_G0271010	DDB0216732	KxDL domain-containing protein	1.30	0.028
108	Q55BB3	DDB_G0271254	DDB0202810	Uncharacterized protein	1.30	0.000
109	Q54T29	DDB_G0282033	DDB0205103	Uncharacterized protein	1.30	0.004
110	Q54IU0	DDB_G0288521	DDB0231476	Aldehyde dehydrogenase	1.30	0.003
111	Q55G69	DDB_G0268346	DDB0202149	PNPLA domain-containing protein	1.29	0.031
112	Q86HW2	DDB_G0349487	DDB0217812	Uncharacterized protein	1.29	0.017
113	Q8MQU6	DDB_G0267426	cshA	Citrate synthase, peroxisomal	1.29	0.000
114	Q54J99	DDB_G0288203	DDB0187827	IFRD domain-containing protein	1.29	0.000
115	Q55DM5	DDB_G0269602	slc35b2	Adenosine 3'-phospho 5'-phosphosulfate transporter 1	1.29	0.000
116	P14327	DDB_G0273775	cinD-1	Vegetative-specific protein H7	1.28	0.018
117	Q54U95	DDB_G0281325	DDB0218292	Uncharacterized protein	1.28	0.000
118	Q54V50	DDB_G0280615	DDB0206075	Uncharacterized protein	1.28	0.035
119	Q54PR1	DDB_G0284375	DDB0185977	PKS_AT domain-containing protein	1.28	0.002
120	Q54ZE4	DDB_G0277653	DDB0217999	Uncharacterized protein	1.28	0.000
121	Q75JS7	DDB_G0272200	DDB0168777	Uncharacterized protein	1.28	0.000
122	Q8T849	DDB_G0275177	prkA	Uncharacterized protein	1.27	0.001
123	P54659	DDB_G0291127	mvpB	Major vault protein beta	1.27	0.000
124	Q8T1P1	DDB_G0273453	DDB_G0273453	Uncharacterized protein DDB_G0273453/DDB_G0273565	1.27	0.006
125	Q54H89	DDB_G0289641	DDB0219472	B box-type domain-containing protein	1.27	0.021
126	Q86IF6	DDB_G0272989	DDB0217073	PX domain-containing protein	1.27	0.000
127	P34118	DDB_G0269156	mvpA	Major vault protein alpha	1.27	0.000
128	Q55GI0	DDB_G0349279	DDB0189444	Uncharacterized protein	1.27	0.000
129	Q559N8	DDB_G0272466	DDB0217020	Uncharacterized protein	1.27	0.007
130	Q86AT8	DDB_G0273531	spkA-1	Stress-activated protein kinase alpha	1.27	0.000
131	Q54CZ7	DDB_G0292612	DDB0184475	Uncharacterized protein	1.27	0.003
132	Q54VA3	DDB_G0280493	DDB0205236	Uncharacterized protein	1.27	0.000

133	Q54H71	DDB_G0289675	adprh	[Protein ADP-ribosylarginine] hydrolase	1.27	0.000
134	Q54EP8	DDB_G0291410	DDB0183875	Methyltransf_11 domain-containing protein	1.27	0.019
135	Q54SE2	DDB_G0282517	DDB_G0282517	1-Cys peroxiredoxin	1.27	0.000
136	Q55BZ5	DDB_G0270296	dcd1A	Protein dcd1A	1.27	0.043
137	P34122	DDB_G0277501	capB	cAMP-binding protein 2	1.26	0.000
138	Q54TQ4	DDB_G0281617	DDB0204563	Uncharacterized protein	1.26	0.002
139	Q54NS1	DDB_G0285011	caf17	Putative transferase caf17 homolog, mitochondrial	1.26	0.000
140	Q556S5	DDB_G0295833	DDB0217341	Short-chain dehydrogenase/reductase family protein	1.26	0.000
141	Q556I6	DDB_G0272612	DDB0167972	TGc domain-containing protein	1.26	0.001
142	Q7KWM5	DDB_G0272730	alg2	Alpha-1,3/1,6-mannosyltransferase ALG2	1.26	0.000
143	Q86A24	DDB_G0272708	DDB_G0272708	Protein-lysine N-methyltransferase	1.26	0.003
144	Q54K28	DDB_G0287671	DDB0219255	Fe2OG dioxygenase domain-containing protein	1.26	0.014
145	Q556J2	DDB_G0272883	ksrA-1	3-ketodihydrosphingosine reductase	1.26	0.000
146	Q54VR5	DDB_G0280183	DDB0206426	Uncharacterized protein	1.26	0.000
147	P54640	DDB_G0272815	cprE	Cysteine proteinase 5	1.26	0.002
148	Q54IN6	DDB_G0288629	DDB_G0288629	Uncharacterized protein	1.26	0.002
149	Q86AQ3	DDB_G0277203	DDB0169112	3Beta_HSD domain-containing protein	1.25	0.001
150	Q559E7	DDB_G0272955	DDB0217056	Uncharacterized protein	1.25	0.002
151	Q54ET7	DDB_G0291344	DDB0183832	Carboxylic ester hydrolase	1.25	0.001
152	Q54JV1	DDB_G0287685	cinC	Elongation factor 2	1.25	0.000
153	Q54N71	DDB_G0285467	DDB0186514	Amidohydro-rel domain-containing protein	1.25	0.000
154	Q55A71	DDB_G0272094	DDB0216957	Uncharacterized protein	1.25	0.033
155	Q556I5	DDB_G0274029	DDB0217100	Uncharacterized protein	1.25	0.000
156	Q86IF9	DDB_G0272678	DDB0168929	Uncharacterized protein	1.25	0.002
157	Q54CJ8	DDB_G0292894	DDB_G0292894	Uncharacterized cyclodeaminase	1.24	0.008
158	Q556V1	DDB_G0273089	coq10-1	Coenzyme Q-binding protein COQ10, mitochondrial	1.24	0.000
159	Q54TD0	DDB_G0281823	V4-7	Peptidase S53 domain-containing protein	1.24	0.000
160	Q55CE9	DDB_G0270088	DDB0190795	Uncharacterized protein	1.24	0.000
161	Q54RW2	DDB_G0282879	DDB0205007	Uncharacterized protein	1.24	0.000
162	Q54P57	DDB_G0284779	DDB0186189	Uncharacterized protein	1.24	0.006
163	Q54H97	DDB_G0289609	DDB_G0289609	CBS domain-containing protein	1.24	0.001
164	Q869Z4	DDB_G0271904	pck2	Phosphoenolpyruvate carboxykinase [GTP], mitochondrial	1.24	0.001
165	Q556G9	DDB_G0272636	DDB0167952	Uncharacterized protein	1.24	0.000
166	Q54SA9	DDB_G0282565	DDB_G0282565	Uncharacterized protein	1.24	0.001
167	Q54DD1	DDB_G0292328	DDB0184335	FAD_binding_3 domain-containing protein	1.24	0.000
168	Q869Z5	DDB_G0271892	DDB0231434	Putative glutathione S-transferase	1.24	0.004
169	Q54K35	DDB_G0287627	timm17	Mitochondrial import inner membrane translocase subunit tim17	1.24	0.011
170	Q54MZ7	DDB_G0285593	DDB0186594	Nfu_N domain-containing protein	1.24	0.000
171	Q55FN4	DDB_G0268026	DDB0189725	Uncharacterized protein	1.24	0.000
172	Q55AD2	DDB_G0271950	DDB_G0271950	B box-type domain-containing protein	1.23	0.000
173	Q556Y8	DDB_G0273729	DDB_G0273199	Probable rhodanese domain-containing dual specificity protein phosphatase	1.23	0.000
174	Q54U38	DDB_G0281291	DDB_G0281291	NAD(P)-bd_dom domain-containing protein	1.23	0.009
175	Q86L51	DDB_G0272857	rapB	Ras-related protein rapB	1.23	0.000
176	Q55CR4	DDB_G0269936	DDB0190684	ANK_REP_REGION domain-containing protein	1.23	0.001
177	Q557I5	DDB_G0273537	DDB0217155	Uncharacterized protein	1.23	0.011
178	Q9NA13	DDB_G0292564	iplA	Inositol 1,4,5-trisphosphate receptor-like protein A	1.23	0.028
179	Q54QU9	DDB_G0283627	prsC	Ribose-phosphate pyrophosphokinase C	1.23	0.002
180	Q86H28	DDB_G0275669	prodh	Proline dehydrogenase 1, mitochondrial	1.23	0.036
181	Q9U1M9	DDB_G0277851	dymB	Dynamin-B	1.23	0.001
182	Q54DL7	DDB_G0292188	DDB_G0292188	von Willebrand factor A domain-containing protein	1.23	0.000
183	Q54LE9	DDB_G0286673	DDB0187087	Ubiquinone biosynthesis protein	1.23	0.005
184	Q54I75	DDB_G0288943	DDB0188188	Uncharacterized protein	1.23	0.000
185	Q556G1	DDB_G0272628	DDB0167945	PKS_ER domain-containing protein	1.22	0.000

186	Q54T58	DDB_G0281987	lyrm1	LYR motif-containing protein 1	1.22	0.021
187	Q54UI8	DDB_G0281055	DDB0203978	Methyltransf_25 domain-containing protein	1.22	0.000
188	Q54F47	DDB_G0291105	cyp513C1	Probable cytochrome P450 513C1	1.22	0.016
189	Q558Y7	DDB_G0272861	cosA	Protein costars	1.22	0.001
190	Q557B6	DDB_G0273237	DDB_G0273237	FAM172 family protein homolog	1.22	0.000
191	Q54VK0	DDB_G0280291	DDB0230117	Methenyl tetrahydrofolate cyclohydrolase / NADP-dependent methylene H4F dehydrogenase	1.22	0.004
192	Q559C4	DDB_G0272971	DDB0217064	PH domain-containing protein	1.22	0.001
193	Q869L6	DDB_G0275559	DDB0167345	Uncharacterized protein	1.22	0.002
194	Q54V68	DDB_G0280585	ubqK	Ubiquitin-like domain-containing protein	1.22	0.003
195	Q86KZ6	DDB_G0272322	DDB0206556	Uncharacterized protein	1.22	0.000
196	Q551R4	DDB_G0276383	DDB0203824	VWFA domain-containing protein	1.22	0.000
197	Q54VI4	DDB_G0280317	gsta3	Putative glutathione S-transferase alpha-3	1.22	0.000
198	Q557D2	DDB_G0273131	g6pd-1	Glucose-6-phosphate 1-dehydrogenase	1.22	0.000
199	Q54U46	DDB_G0281283	DDB0204129	N-acetyltransferase domain-containing protein	1.22	0.000
200	Q55GJ5	DDB_G0267644	DDB0189429	Uncharacterized protein	1.22	0.049
201	Q86A16	DDB_G0272680	DDB0168928	PPM-type phosphatase domain-containing protein	1.22	0.000
202	Q54XG9	DDB_G0278975	DDB_G0278975	G8 domain-containing protein	1.21	0.000
203	Q54BT7	DDB_G0293448	DDB0229809	J domain-containing protein (Fragment)	1.21	0.000
204	Q54L77	DDB_G0286849	DDB_G0286849	Uncharacterized protein	1.21	0.000
205	Q55CV1	DDB_G0269888	DDB0190648	Uncharacterized protein	1.21	0.013
206	Q558S4	DDB_G0272841	DDB0202692	Purple acid phosphatase	1.21	0.028
207	Q54W09	DDB_G0279973	DDB_G0279973	AhpC/TSA family protein	1.21	0.001
208	C7G071	DDB_G0295739	DDB_G0295739	APH domain-containing protein	1.21	0.000
209	Q54M18	DDB_G0286257	argC	Bifunctional protein argC, mitochondrial	1.21	0.000
210	Q76NT0	DDB_G0277479	mrm1	rRNA methyltransferase 1, mitochondrial	1.21	0.003
211	Q54TE4	DDB_G0281793	DDB0204678	Uncharacterized protein	1.21	0.005
212	P02886	DDB_G0273063	dscA-1	Discoidin-1 subunit A	1.21	0.025
213	Q55DR6	DDB_G0269242	fcsA	Fatty acyl-CoA synthetase A	1.21	0.000
214	Q55GK8	DDB_G0267630	kil1	Membrane-associated sulfotransferase kil1	1.21	0.000
215	Q55EM4	DDB_G0268828	DDB0190067	Uncharacterized protein	1.21	0.004
216	Q54H87	DDB_G0289621	DDB0188495	Uncharacterized protein	1.21	0.003
217	Q54DG8	DDB_G0292282	DDB0184300	Uncharacterized protein	1.21	0.012
218	P54658	DDB_G0272819	hspC	32 kDa heat shock protein	1.21	0.005
219	Q54KD0	DDB_G0287407	DDB_G0287407	TPR repeat-containing protein	1.21	0.000
220	Q86L12	DDB_G0276853	DDB0169011	Uncharacterized protein	1.21	0.001
221	Q54F95	DDB_G0290995	DDB0189200	PHB domain-containing protein	1.21	0.000
222	Q86K21	DDB_G0272875	cpnB-1	Copine-B	1.21	0.000
223	Q54RY0	DDB_G0282845	DDB0204986	J domain-containing protein	1.21	0.003
224	Q7KWR1	DDB_G0276279	DDB_G0276279	DIOX_N domain-containing protein	1.21	0.000
225	Q55BV1	DDB_G0270352	DDB0190990	Uncharacterized protein	1.21	0.000
226	Q54BE6	DDB_G0293664	DDB0192080	Uncharacterized protein	1.21	0.004
227	Q7KWW3	DDB_G0276789	DDB_G0276789	Uncharacterized protein	1.21	0.004
228	Q54G64	DDB_G0290377	agnB	Argonaut-like protein	1.21	0.000
229	Q54KP5	DDB_G0287215	DDB0187366	J domain-containing protein	1.20	0.001
230	Q54QE7	DDB_G0283915	DDB_G0283915	Esterase/lipase/thioesterase domain-containing protein	1.20	0.000
231	Q54IR6	DDB_G0288573	DDB0187998	Uncharacterized protein	1.20	0.015
232	Q54VS1	DDB_G0280173	isca1	Iron-sulfur cluster assembly 1 homolog, mitochondrial	1.20	0.003
233	Q54YA6	DDB_G0278333	DDB0205381	Uncharacterized protein	1.20	0.001
234	Q54N01	DDB_G0285587	DDB0186587	Uncharacterized protein	1.20	0.032
235	Q54BC2	DDB_G0293758	H2Bv1	Histone H2B.v1	1.20	0.019
236	Q54LI8	DDB_G0286619	DDB0220665	SAP DNA-binding domain-containing protein	1.20	0.001
237	Q558Z1	DDB_G0272995	DDB0217077	Uncharacterized protein	1.20	0.003
238	Q54IW4	DDB_G0288477	DDB0219348	Uncharacterized protein	1.20	0.006

Down-regulated proteins in ATG16¯ versus AX2 cells

#	UniProt ID	DDB_G ID	GeneName	GeneProduct	FC	p-value
1	Q54G81	DDB_G0290325	DDB0188839	Peptidase C50 domain-containing protein	0.51	0.006
2	Q86KU5	DDB_G0277729	DDB_G0277729	Uncharacterized protein	0.53	0.000
3	O15736	DDB_G0275323	tipD	Protein tipD (ATG16)	0.53	0.001
4	C7G078	DDB_G0295801	DDB_G0295801	B_lectin domain-containing protein	0.55	0.000
5	Q95US4	DDB_G0279921	gp130	Lipid-anchored plasma membrane glycoprotein 130	0.55	0.000
6	Q54BS2	DDB_G0293460	DDB0191955	Purple acid phosphatase	0.57	0.004
7	Q54G31	DDB_G0290465	DDB0219564	Uncharacterized protein	0.59	0.000
8	Q86JJ3		DDB0168369	Uncharacterized protein	0.59	0.016
9	Q7Z1I0	DDB_G0289813	cupF	Calcium up-regulated protein F	0.60	0.002
10	Q54TC9	DDB_G0281821	sre1	Elongation of fatty acids protein sre1	0.60	0.000
11	Q86JM5	DDB_G0272012	DDB_G0272012	Putative elongation of fatty acids protein	0.61	0.000
12	Q54X49	DDB_G0279211	metE	5-methyltetrahydropteroyltriglutamate--homocysteine methyltransferase	0.61	0.000
13	C7G077	DDB_G0295799	DDB_G0295799	BB_PF domain-containing protein	0.62	0.003
14	Q54IN0	DDB_G0288635	DDB0188033	Uncharacterized protein	0.62	0.001
15	Q54YR8	DDB_G0278115	DDB0204431	Uncharacterized protein	0.63	0.000
16	Q550U9	DDB_G0276767	plbA	Phospholipase B-like protein A	0.64	0.019
17	P54657	DDB_G0285793	cadA	Calcium-dependent cell adhesion molecule 1	0.64	0.000
18	Q54LB9	DDB_G0286723	ponC5	Ponticulin-like protein C5	0.65	0.007
19	Q54I15	DDB_G0289109	DDB0219409	Uncharacterized protein	0.65	0.015
20	Q54TC4	DDB_G0281861	DDB0204253	Purple acid phosphatase	0.66	0.035
21	P15808	DDB_G0280045	thyA	Flavin-dependent thymidylate synthase	0.66	0.000
22	Q54IE5	DDB_G0288807	DDB0188120	Uncharacterized protein	0.66	0.001
23	Q54SB4	DDB_G0282559	DDB0204837	Purple acid phosphatase	0.67	0.003
24	Q7Z1Z9	DDB_G0289883	cupG	Calcium up-regulated protein G	0.67	0.000
25	Q58A41	DDB_G0289467	DD8-14	G domain-containing protein	0.68	0.000
26	Q555N6	DDB_G0274705	DDB_G0274705	Glutathione S-transferase domain-containing protein	0.68	0.019
27	Q86L03	DDB_G0276873	DDB0169034	Uncharacterized protein	0.68	0.029
28	C7G070	DDB_G0295737	DDB_G0295737	PAP/25A-associated domain-containing protein	0.68	0.001
29	Q54YA0	DDB_G0278345	acly	Probable ATP-citrate synthase	0.68	0.000
30	Q552U9	DDB_G0275721	DDB0202450	Uncharacterized protein	0.68	0.002
31	Q54SG3	DDB_G0282483	DDB_G0282483	Uncharacterized transmembrane protein	0.68	0.001
32	Q86HF8	DDB_G0271852	DDB0168536	Uncharacterized protein	0.69	0.002
33	Q54PD4	DDB_G0284629	DDB_G0284629	SnoaL-like domain-containing protein	0.70	0.000
34	Q8MML5	DDB_G0274109	paxB	Paxillin-B	0.70	0.004
35	Q54QX0	DDB_G0283533	cbpB	Calcium-binding protein B	0.70	0.009
36	P30815	DDB_G0281393	cxdA	Cytochrome c oxidase subunit 4, mitochondrial	0.70	0.010
37	Q54UR0	DDB_G0280881	DDB_G0280881	Glutathione S-transferase domain-containing protein	0.70	0.008
38	Q54YA2	DDB_G0278341	DDB0205386	Uncharacterized protein	0.70	0.000
39	Q54LW6	DDB_G0286463	DDB_G0286463	Uncharacterized protein	0.71	0.016
40	Q54G38	DDB_G0290425	surf6	Surfeit locus protein 6 homolog	0.71	0.001
41	Q54JF0	DDB_G0288091	DDB0187779	Uncharacterized protein	0.72	0.000
42	Q54CL1	DDB_G0292874	DDB0191664	Nefa_Nip30_N domain-containing protein	0.72	0.007
43	Q54XY5	DDB_G0278699	DDB0218110	Uncharacterized protein	0.72	0.024
44	Q86JE7	DDB_G0277573	DDB0169278	Uncharacterized protein	0.72	0.003
45	Q54FV5	DDB_G0290577	DDB0188966	Uncharacterized protein	0.72	0.001
46	Q550I1	DDB_G0277097	DDB0217906	Endotoxin_N domain-containing protein	0.72	0.000
47	B0G0Y8	DDB_G0268634	pde3	cGMP-specific 3',5'-cGMP phosphodiesterase 3	0.73	0.006
48	Q54N59	DDB_G0285485	DDB0186526	Uncharacterized protein	0.73	0.000
49	Q86J14	DDB_G0274273	DDB0167842	C2 domain-containing protein	0.73	0.001
50	Q54X38	DDB_G0279229	DDB0205663	Uncharacterized protein	0.73	0.002

51	Q54I60	DDB_G0288985	purN	Phosphoribosylglycinamide formyltransferase	0.73	0.000
52	Q55DS6	DDB_G0269544	DDB0190345	Uncharacterized protein	0.73	0.008
53	Q54JH5	DDB_G0288059	DDB0219299	Uncharacterized protein	0.73	0.001
54	Q55C87	DDB_G0270166	DDB0190855	Uncharacterized protein	0.73	0.003
55	Q7Z203	DDB_G0289815	cupB	Calcium up-regulated protein B	0.74	0.042
56	O77257	DDB_G0278725	p17	Secreted protein A	0.74	0.001
57	Q76P10	DDB_G0277367	DDB_G0277367	Uncharacterized protein	0.74	0.002
58	Q54UJ1	DDB_G0281049	DDB0203975	LIM zinc-binding domain-containing protein	0.74	0.000
59	Q54RI2	DDB_G0283127	DDB0185376	Uncharacterized protein	0.74	0.021
60	Q54GY6	DDB_G0289859	kxcA	Kinase and exchange factor for Rac A	0.74	0.012
61	Q55G75	DDB_G0267786	DDB_G0267786	PH domain-containing protein	0.75	0.000
62	B0G0Y7	DDB_G0268646	DDB_G0268646	Uncharacterized protein	0.75	0.000
63	Q54WE9	DDB_G0279695	DDB0205991	RRM domain-containing protein	0.75	0.001
64	Q23911	DDB_G0291255	29C	Secreted protein B	0.75	0.004
65	Q54Z53	DDB_G0277775	DDB0203448	Peptidyl-prolyl cis-trans isomerase	0.75	0.001
66	Q54X05	DDB_G0279397	DDB0218164	SAC domain-containing protein	0.75	0.017
67	P20609	DDB_G0277837	cxgE	Cytochrome c oxidase subunit 7e	0.75	0.000
68	Q54Q84	DDB_G0284051	DDB0185804	Aa_trans domain-containing protein	0.76	0.002
69	Q54CS6	DDB_G0292768	dcd1B	Protein dcd1B	0.76	0.000
70	Q86AV9	DDB_G0276919	pldG	Phosphatidylinositol-glycan-specific phospholipase D	0.76	0.009
71	P32073	DDB_G0281551	guaA	GMP synthase [glutamine-hydrolyzing]	0.76	0.000
72	Q54PS7	DDB_G0284449	plbD	Phospholipase B-like protein D	0.76	0.020
73	P34115	DDB_G0290257	GP138A	Cell surface glycoprotein gp138A	0.76	0.005
74	Q55CB2	DDB_G0270134	DDB0190831	ADF-H domain-containing protein	0.76	0.002
75	B0G165	DDB_G0295675	DDB_G0295675	Uncharacterized protein	0.76	0.000
76	Q54NC1	DDB_G0285399	cyb5r1	NADH-cytochrome b5 reductase 1	0.76	0.000
77	P07670	DDB_G0276331	pyr4	Dihydroorotate dehydrogenase	0.76	0.000
78	Q54N80	DDB_G0285455	fkbp3	FK506-binding protein 3	0.76	0.006
79	Q76P07	DDB_G0277165	DDB_G0277165	Probable serine/threonine-protein kinase	0.76	0.002
80	Q54QJ8	DDB_G0283871	DDB_G0283871	Uncharacterized protein	0.76	0.000
81	Q8T196	DDB_G0274203	DDB0167563	SH3 domain-containing protein	0.77	0.009
82	Q86KU6	DDB_G0277725	thfA	Methylenetetrahydrofolate dehydrogenase [NAD(+)]	0.77	0.000
83	Q54N31	DDB_G0285541	DDB0186554	ZT_dimer domain-containing protein	0.77	0.000
84	Q1ZXP2	DDB_G0294587	DDB_G0294587	Uncharacterized protein (Fragment)	0.77	0.030
85	Q54ND0	DDB_G0285345	DDB_G0285345	Uncharacterized transmembrane protein SSD449	0.77	0.045
86	Q55DB1	DDB_G0269716	DDB0190498	Peptidase_C39_2 domain-containing protein	0.77	0.009
87	Q86JN6	DDB_G0271992	DDB0168606	Uncharacterized protein	0.77	0.005
88	Q54PA8	DDB_G0284671	DDB_G0284671	Uncharacterized protein	0.77	0.042
89	P26310	DDB_G0282097	cxfA	Cytochrome c oxidase polypeptide 6, mitochondrial	0.77	0.000
90	Q55D96	DDB_G0269734	DDB0190512	SPX domain-containing protein	0.77	0.020
91	Q54SY2	DDB_G0282181	nvl	Putative ribosome biogenesis ATPase nvl	0.77	0.004
92	P07828	DDB_G0289489	act18	Actin-18	0.77	0.017
93	Q55DU1	DDB_G0269518	abnB	Actobindin-B/C	0.78	0.002
94	Q54PD3	DDB_G0284631	DDB_G0284631	Uncharacterized protein	0.78	0.000
95	Q55BY4	DDB_G0271066	DDB0216761	Elongation of fatty acids protein	0.78	0.039
96	Q54I40	DDB_G0289027	DDB0188218	Uncharacterized protein	0.78	0.007
97	Q54Q03	DDB_G0284205	ubl5	Ubiquitin-like protein 5	0.78	0.011
98	Q54UY3	DDB_G0280725	DDB0206158	Uncharacterized protein	0.78	0.029
99	O21042	DDB_G0294088	cox1/2	Cytochrome c oxidase subunit 1+2	0.78	0.001
100	Q54EX9	DDB_G0291277	DDB0183789	Uncharacterized protein	0.78	0.013
101	Q54ZI6	DDB_G0277455	plbE	Phospholipase B-like protein E	0.78	0.003
102	Q54VG5	DDB_G0280343	DDB0206531	Uncharacterized protein	0.78	0.001
103	Q54N55	DDB_G0285493	DDB0186530	BTP domain-containing protein	0.78	0.011
104	Q54V71	DDB_G0280579	DDB_G0280579	Uncharacterized protein	0.78	0.030
105	Q7KWY5	DDB_G0276769	DDB_G0276769	Uncharacterized protein	0.78	0.005
106	Q54BI6	DDB_G0293606	DDB0192040	CRAL-TRIO domain-containing protein	0.79	0.000

107	Q55EK6	DDB_G0268848	DDB0190082	Uncharacterized protein	0.79	0.000
108	Q54CW1	DDB_G0292664	DDB0184511	Calponin-homology (CH) domain-containing protein	0.79	0.003
109	Q86A81	DDB_G0274327	DDB0167879	TFIIS N-terminal domain-containing protein	0.79	0.008
110	Q869W9	DDB_G0275069	pks16	Probable polyketide synthase 16	0.79	0.000
111	Q54VT4	DDB_G0280147	ddx47	Probable ATP-dependent RNA helicase ddx47	0.79	0.007
112	Q54BW5	DDB_G0293374	dut	Deoxyuridine 5'-triphosphate nucleotidohydrolase	0.79	0.000
113	Q54G96	DDB_G0290301	DDB_G0290301	Uncharacterized protein	0.79	0.001
114	Q54NR8	DDB_G0285013	DDB0186325	N-acetyltransferase domain-containing protein	0.79	0.035
115	Q54WT8	DDB_G0279443	aqpB	Aquaporin-B	0.79	0.037
116	Q54UH8	DDB_G0281071	serA	D-3-phosphoglycerate dehydrogenase	0.79	0.000
117	Q54QT0	DDB_G0283653	DDB0185606	Uncharacterized protein	0.79	0.018
118	Q54DN6	DDB_G0292112	galK	Galactokinase	0.79	0.002
119	Q54DV6	DDB_G0292012	DDB0184160	N-acetyltransferase domain-containing protein	0.79	0.040
120	Q54ED0	DDB_G0291574	DDB0183990	NPC1_N domain-containing protein	0.79	0.002
121	Q55D50	DDB_G0269790	DDB0190556	Uncharacterized protein	0.79	0.002
122	Q55FI9	DDB_G0268090	DDB0189769	Glyco_hydro_18 domain-containing protein	0.79	0.000
123	Q54P26	DDB_G0284859	samkB	Probable serine/threonine-protein kinase samkB	0.80	0.000
124	Q55EB8	DDB_G0269312	DDB0190166	NUC153 domain-containing protein	0.80	0.009
125	Q54XU9	DDB_G0278571	DDB0205545	HIG1 domain-containing protein	0.80	0.010
126	Q54MM3	DDB_G0285859	DDB0186714	Uncharacterized protein	0.80	0.010
127	Q553P3	DDB_G0275385	DDB0202598	Fe2OG dioxygenase domain-containing protein	0.80	0.001
128	Q54YX3	DDB_G0278031	DDB_G0278031	CRAL-TRIO domain-containing protein	0.80	0.000
129	Q54BX0	DDB_G0293366	DDB0191902	NLPC_P60 domain-containing protein	0.80	0.005
130	Q55CM9	DDB_G0269992	DDB0190722	Uncharacterized protein	0.80	0.013
131	Q55FD9	DDB_G0268576	DDB0216585	PH domain-containing protein	0.80	0.017
132	Q54QQ0	DDB_G0283701	impdh	Inosine-5'-monophosphate dehydrogenase	0.80	0.000
133	Q54KH9	DDB_G0287335	DDB0187427	RRM domain-containing protein	0.80	0.000
134	Q54H38	DDB_G0289723	abhd	Abhydrolase domain-containing protein	0.80	0.005
135	Q54SR7	DDB_G0282267	fkbp2	FK506-binding protein 2	0.80	0.002
136	Q54SQ6	DDB_G0282301	DDB_G0282301	Uncharacterized protein	0.81	0.006
137	Q54IN4	DDB_G0288631	DDB0188030	Peptidase_S9 domain-containing protein	0.81	0.035
138	Q54RC0	DDB_G0283327	DDB0218473	Uncharacterized protein	0.81	0.002
139	P09556	DDB_G0280041	pyr56	Uridine 5'-monophosphate synthase	0.81	0.000
140	Q54XG0	DDB_G0278987	DDB_G0278987	Uncharacterized G-patch domain protein	0.81	0.040
141	Q54BL2	DDB_G0293576	esf2	Putative pre-rRNA-processing protein esf2	0.81	0.011
142	Q54FT0	DDB_G0290679	DDB0219588	Uncharacterized protein	0.81	0.000
143	P13651	DDB_G0284861	eif5a	Eukaryotic translation initiation factor 5A	0.81	0.000
144	Q75JV7	DDB_G0272170	gnt8	Putative beta-1,4-N-acetylglucosaminyltransferase	0.81	0.001
145	Q54VG2	DDB_G0280349	DDB0206534	Uncharacterized protein	0.81	0.002
146	Q55FT1	DDB_G0267966	pyd1	Dihydropyrimidine dehydrogenase [NADP(+)]	0.81	0.000
147	Q54N05	DDB_G0285581	DDB0186583	RING-type domain-containing protein	0.81	0.005
148	Q8SSN5	DDB_G0276345	nat5	N-alpha-acetyltransferase 20	0.81	0.021
149	Q54D57	DDB_G0292472	DDB0184418	Uncharacterized protein	0.81	0.002
150	Q55EK2	DDB_G0269016	cyp524A1	Probable cytochrome P450 524A1	0.81	0.000
151	Q55FP7	DDB_G0268008	DDB0189713	DAO domain-containing protein	0.81	0.004
152	P24639	DDB_G0269160	nxnA	Annexin A7	0.82	0.000
153	Q54U07	DDB_G0281403	nacA	Nascent polypeptide-associated complex subunit alpha	0.82	0.002
154	Q54TD6	DDB_G0281843	DDB0204241	Uncharacterized protein	0.82	0.011
155	O21049	DDB_G0294092	cox3	Cytochrome c oxidase subunit 3	0.82	0.000
156	Q86KB5	DDB_G0274205	DDB0167562	Uncharacterized protein	0.82	0.004
157	Q54W91	DDB_G0279821	DDB0206310	Uncharacterized protein	0.82	0.012
158	Q86I47	DDB_G0275053	DDB0167407	NmrA domain-containing protein	0.82	0.001
159	Q86IC7	DDB_G0275503	DDB0167175	Sde2_N_Ubi domain-containing protein	0.82	0.010
160	Q55GW2	DDB_G0267484	acp1	Low molecular weight phosphotyrosine protein phosphatase	0.82	0.007
161	Q54HT2	DDB_G0289235	DDB0188327	Uncharacterized protein	0.82	0.009

162	Q54MK3	DDB_G0285889	DDB0186733	Uncharacterized protein	0.82	0.001
163	Q54MD6	DDB_G0286103	DDB0220504	Small MutS related (Smr) family protein	0.82	0.000
164	Q54CJ1	DDB_G0292978	DDB0219806	Uncharacterized protein	0.82	0.007
165	Q55CM7	DDB_G0269994	DDB_G0269994	DDRGK domain-containing protein 1	0.82	0.042
166	Q54BC3	DDB_G0293786	DDB_G0293786	Ras GTPase	0.82	0.044
167	Q54JI7	DDB_G0288025	hal	Probable histidine ammonia-lyase	0.82	0.001
168	Q54J53	DDB_G0288289	DDB0187875	Uncharacterized protein	0.82	0.001
169	Q55CP1	DDB_G0269972	DDB0190708	Sas10 domain-containing protein	0.82	0.002
170	B0G119	DDB_G0295681	DDB_G0295681	Peptidylprolyl isomerase	0.82	0.001
171	Q54BH5	DDB_G0293730	DDB_G0293730	PI-PLC X-box domain-containing protein	0.82	0.000
172	Q54YL9	DDB_G0278179	DDB0216404	Myb domain-containing protein	0.83	0.000
173	Q55DB6	DDB_G0269712	ahsa	Activator of 90 kDa heat shock protein ATPase homolog	0.83	0.002
174	Q54Y04	DDB_G0278491	DDB0205489	DTW domain-containing protein	0.83	0.004
175	Q55FK4	DDB_G0268064	DDB_G0268064	Acyl-protein thioesterase 1 homolog 2	0.83	0.000
176	P46794	DDB_G0267386	cysB	Cystathionine beta-synthase	0.83	0.000
177	Q55AK6	DDB_G0271766	DDB0203625	Poly [ADP-ribose] polymerase	0.83	0.008
178	Q86HX9	DDB_G0276877	mybK	Myb-like protein K	0.83	0.010
179	Q54FV6	DDB_G0290575	DDB0188965	Uncharacterized protein	0.83	0.002
180	Q55C09	DDB_G0270834	sgmA	Sphingomyelin phosphodiesterase A	0.83	0.008
181	Q54J34	DDB_G0288333	purB	Adenylosuccinate lyase	0.83	0.000
182	Q54HN9	DDB_G0289333	DDB0188365	Uncharacterized protein	0.83	0.001
183	Q55FI2	DDB_G0268102	DDB0189776	Uncharacterized protein	0.83	0.047
184	Q54QF3	DDB_G0283851	DDB0185734	DSPc domain-containing protein	0.83	0.007
185	Q54RP4	DDB_G0283067	DDB0218432	Uncharacterized protein	0.83	0.004
186	Q54X76	DDB_G0279155	DDB0218152	THUMP domain-containing protein	0.83	0.004
187	Q54C42	DDB_G0293244	DDB0191845	Uncharacterized protein	0.83	0.001
188	Q54Q60	DDB_G0284079	DDB0185830	Uncharacterized protein	0.83	0.008
189	Q1ZXC2	DDB_G0290745	pabpc1B	Polyadenylate-binding protein 1-B	0.83	0.001
190	Q8T6J5	DDB_G0267438	abcA2	ABC transporter A family member 2	0.83	0.001
191	Q551A6	DDB_G0276719	DDB0217846	Uncharacterized protein	0.83	0.023
192	Q54PW5	DDB_G0284257	dph1	2-(3-amino-3-carboxypropyl)histidine synthase subunit 1	0.83	0.024
193	Q54SA1	DDB_G0282579	pldZ	Phospholipase D Z	0.83	0.009

Up-regulated proteins in ATG9^{-}/16^{-} versus AX2 cells

#	UniProt ID	DDB_G ID	GeneName	GeneProduct	FC	p-value
1	Q54LB9	DDB_G0286723	ponC5	Ponticulin-like protein C5	5.07	0.001
2	Q55H11	DDB_G0267292	DDB0216492	Uncharacterized protein	4.75	0.000
3	O15723	N/A	gag	Gag	3.83	0.000
4	Q86AV9	DDB_G0276919	pldG	Phosphatidylinositol-glycan-specific phospholipase D	2.81	0.002
5	P34146	DDB_G0282365	rac1C	Rho-related protein rac1C	2.79	0.002
6	Q55BN3	DDB_G0270430	DDB0191055	WH2 domain-containing protein	2.56	0.015
7	Q54BL7	DDB_G0293708	DDB0192012	LIM zinc-binding domain-containing protein	2.56	0.000
8	Q54XJ6	DDB_G0278897	DDB0206269	RGS domain-containing protein	2.51	0.000
9	Q54LV6	DDB_G0286393	DDB_G0286393	Uncharacterized protein	2.35	0.001
10	Q54XA6	DDB_G0279093	DDB0205031	BTB_2 domain-containing protein	2.33	0.009
11	Q55CL7	DDB_G0271010	DDB0216732	KxDL domain-containing protein	2.23	0.017
12	Q556S3	DDB_G0273119	DDB_G0273119	Uncharacterized protein	2.15	0.001
13	Q559T8	DDB_G0272282	DDB_G0272282	Probable serine/threonine-protein kinase	2.13	0.035
14	Q54W28	DDB_G0279955	DDB0205886	Uncharacterized protein	2.07	0.001
15	O96904	DDB_G0269130	phgA	Drainin	2.05	0.029
16	Q556H9	DDB_G0274049	DDB0202984	Uncharacterized protein	2.03	0.000
17	Q55GY6	DDB_G0267342	DDB0216513	Reverse transcriptase domain-containing protein	2.00	0.006

18	Q54X38	DDB_G0279229	DDB0205663	Uncharacterized protein	1.96	0.001
19	Q9BIW4	DDB_G0287507	limD	LIM domain-containing protein D	1.95	0.011
20	Q54K99	DDB_G0287509	ubiad1	UbiA prenyltransferase domain-containing protein 1	1.94	0.000
21	Q54JX4	DDB_G0287879	DDB0219270	Uncharacterized protein	1.92	0.003
22	Q54G11	DDB_G0290491	DDB_G0290491	Autophagy-related protein 8-like protein	1.91	0.004
23	Q54FX8	DDB_G0290655	DDB_G0290655	Putative uncharacterized protein	1.90	0.019
24	Q54S99	DDB_G0282583	DDB_G0282583	RGS domain-containing protein	1.87	0.007
25	Q54U89	DDB_G0281211	far1	Metabotropic glutamate receptor-like protein L	1.87	0.017
26	Q54RN9	DDB_G0283007	DDB0185319	Uncharacterized protein	1.86	0.000
27	Q86AC9	DDB_G0276361	DDB0167013	Uncharacterized protein	1.84	0.000
28	Q54FD5	DDB_G0290931	DDB_G0290931	TNF receptor-associated factor family protein	1.82	0.000
29	Q54WE3	DDB_G0279707	DDB0205997	Uncharacterized protein	1.80	0.001
30	Q94494	DDB_G0289393	psiH	Protein psiH	1.79	0.037
31	Q54CB7	DDB_G0293066	DDB0191758	Uncharacterized protein	1.79	0.017
32	Q552S8	DDB_G0275911	DDB0217719	Uncharacterized protein	1.79	0.001
33	Q54M11	DDB_G0286271	DDB0186894	Uncharacterized protein	1.78	0.000
34	Q55CE3	DDB_G0270098	DDB0190801	Uncharacterized protein	1.77	0.000
35	Q86K21	DDB_G0272875	cpnB-1	Copine-B	1.75	0.000
36	Q54ZD5	DDB_G0277625	DDB0203417	DUF202 domain-containing protein	1.75	0.015
37	Q556U5	DDB_G0273095	DDB_G0273095	28 kDa heat- and acid-stable phosphoprotein homolog	1.74	0.000
38	Q54IM6	DDB_G0288651	DDB0219369	PNPLA domain-containing protein	1.74	0.000
39	Q550F6	DDB_G0277115	dcx	Protein doublecortin	1.74	0.001
40	Q54IR6	DDB_G0288573	DDB0187998	Uncharacterized protein	1.72	0.001
41	Q54RI2	DDB_G0283127	DDB0185376	Uncharacterized protein	1.71	0.016
42	Q55A32	DDB_G0272364	DDB_G0272364	EGF-like domain-containing protein	1.70	0.001
43	Q54NZ7	DDB_G0285053	alrB	Aldose reductase B	1.70	0.000
44	Q54HM2	DDB_G0289357	DDB_G0289357	Uncharacterized protein	1.70	0.010
45	Q8T2A6	DDB_G0274215	DDB0167576	BTB domain-containing protein	1.69	0.000
46	Q554F5	DDB_G0275209	cnrD	Putative countin receptor Cnr4	1.68	0.004
47	Q54LF1	DDB_G0286669	DDB_G0286669	Acyl-coenzyme A oxidase	1.68	0.000
48	P34042	DDB_G0285425	gpaD	Guanine nucleotide-binding protein alpha-4 subunit	1.68	0.003
49	Q55BQ2	DDB_G0271086	DDB0216772	PH domain-containing protein	1.68	0.000
50	Q54WK9	DDB_G0279575	DDB0205857	DUF1084 domain-containing protein	1.67	0.013
51	P42529	DDB_G0276759	cbpA	Calcium-binding protein A	1.66	0.002
52	Q556V7	DDB_G0273085	DDB0168076	Uncharacterized protein	1.65	0.000
53	Q86I30	DDB_G0275083	DDB0167433	Uncharacterized protein	1.64	0.002
54	Q54X05	DDB_G0279397	DDB0218164	SAC domain-containing protein	1.62	0.003
55	Q5TJ65	DDB_G0289541	vasp	Protein VASP homolog	1.59	0.001
56	Q557J6	DDB_G0273517	abpE-1	Drebrin-like protein	1.57	0.002
57	Q556S2	DDB_G0273865	pakH-1	Serine/threonine-protein kinase pakH	1.56	0.001
58	Q54GB3	DDB_G0290363	sybB	Synaptobrevin-B	1.55	0.000
59	Q54K91	DDB_G0287519	ach1	Acetyl-CoA hydrolase	1.55	0.000
60	Q54H33	DDB_G0289733	DDB0188548	Uncharacterized protein	1.54	0.002
61	Q55E54	DDB_G0269388	corB	Coronin-B	1.50	0.000
62	P14327	DDB_G0273775	cinD-1	Vegetative-specific protein H7	1.50	0.001
63	Q54RN7	DDB_G0283011	DDB_G0283011	t-SNARE coiled-coil homology domain-containing protein	1.49	0.042
64	Q55CT6	DDB_G0270990	DDB0216722	Acyl-coenzyme A oxidase	1.49	0.000
65	Q54WY8	DDB_G0279315	gacN	Rho GTPase-activating protein gacN	1.49	0.002
66	Q54NS7	DDB_G0285007	prafB	PRA1 family protein 2	1.48	0.001
67	Q54B10	DDB_G0293904	redA	NADPH oxidoreductase A	1.47	0.000
68	Q556T4	DDB_G0273105	DDB0168056	Cytochrome b5 heme-binding domain-containing protein	1.47	0.002
69	P34137	DDB_G0273817	ptpA1-1	Tyrosine-protein phosphatase 1	1.47	0.002
70	Q54PF2	DDB_G0284599	DDB0186090	RING-type domain-containing protein	1.46	0.034
71	Q551L7	DDB_G0276551	DDB0217807	G domain-containing protein	1.46	0.021

72	Q54VY1	DDB_G0280069	DDB0206357	DUF2185 domain-containing protein	1.46	0.003
73	Q1ZXP2	DDB_G0294587	DDB_G0294587	Uncharacterized protein (Fragment)	1.46	0.003
74	O21042	DDB_G0294088	cox1/2	Cytochrome c oxidase subunit 1+2	1.45	0.002
75	Q86JN6	DDB_G0271992	DDB0168606	Uncharacterized protein	1.45	0.017
76	Q86AT8	DDB_G0273531	spkA-1	Stress-activated protein kinase alpha	1.45	0.000
77	Q54JV1	DDB_G0287685	cinC	Elongation factor 2	1.45	0.002
78	Q54GQ5	DDB_G0289995	DDB0188675	Uncharacterized protein	1.45	0.036
79	Q55BF4	DDB_G0271310	ucpA	Mitochondrial substrate carrier family protein ucpA	1.45	0.015
80	Q54H05	DDB_G0289661	kinY	Probable serine/threonine-protein kinase kinY	1.44	0.001
81	Q55GB1	DDB_G0267432	abcG15	ABC transporter G family member 15	1.44	0.003
82	O60952	DDB_G0279415	limE	LIM domain-containing protein E	1.44	0.000
83	Q75JT4	DDB_G0272150	grlJ	Metabotropic glutamate receptor-like protein J	1.44	0.004
84	O21049	DDB_G0294092	cox3	Cytochrome c oxidase subunit 3	1.43	0.000
85	O77257	DDB_G0278725	p17	Secreted protein A	1.43	0.000
86	Q86IG9	DDB_G0272694	gacH	Rho GTPase-activating protein gacH	1.43	0.040
87	Q54GV4	DDB_G0289907	DDB_G0289907	EGF-like domain-containing protein	1.43	0.044
88	Q556T8	DDB_G0273829	DDB0203031	SCP domain-containing protein	1.43	0.007
89	Q54KI6	DDB_G0287323	DDB0187419	Uncharacterized protein	1.42	0.001
90	Q54U95	DDB_G0281325	DDB0218292	Uncharacterized protein	1.41	0.000
91	Q54FV6	DDB_G0290575	DDB0188965	Uncharacterized protein	1.41	0.002
92	Q54YU5	DDB_G0278593	exdl2B	3'-5' exonuclease domain-containing protein	1.40	0.001
93	Q558Y7	DDB_G0272861	cosA	Protein costars	1.40	0.002
94	Q556R9	DDB_G0273177	DDB_G0273871	UPF0734 protein DDB_G0273871/DDB_G0273177	1.40	0.000
95	Q966R0	DDB_G0272827	cbpI	Calcium-binding protein I	1.40	0.013
96	Q558S4	DDB_G0272841	DDB0202692	Purple acid phosphatase	1.40	0.004
97	Q556H5	DDB_G0272646	cofD-1	Cofilin-3	1.40	0.007
98	Q54VG1	DDB_G0280351	gpaL	Guanine nucleotide-binding protein alpha-12 subunit	1.40	0.000
99	Q54LN9	DDB_G0286525	acmsd	2-amino-3-carboxymuconate-6-semialdehyde decarboxylase	1.40	0.002
100	Q8T849	DDB_G0275177	prkA	Uncharacterized protein	1.39	0.000
101	Q555D2	DDB_G0274915	DDB_G0274915	Nuclear pore complex protein	1.39	0.009
102	Q556I2	DDB_G0273031	ado-1	Probable 2-aminoethanethiol dioxygenase	1.39	0.000
103	Q54I40	DDB_G0289027	DDB0188218	Uncharacterized protein	1.39	0.000
104	P11022	DDB_G0269170	pmpA	Membrane protein P8A7	1.38	0.001
105	Q9NGW9	DDB_G0290723	mkcB	Probable serine/threonine-protein kinase mkcB	1.38	0.001
106	Q556U6	DDB_G0273093	bip1-1	Luminal-binding protein 1	1.38	0.000
107	Q54QY8	DDB_G0283481	DDB0185548	Uncharacterized protein	1.38	0.000
108	Q54W89	DDB_G0279825	DDB0206312	Uncharacterized protein	1.38	0.036
109	Q55CH0	DDB_G0271022	adcC	Arrestin domain-containing protein C	1.37	0.003
110	Q86AY4	DDB_G0272340	DDB_G0272340	TNF receptor-associated factor family protein	1.37	0.006
111	Q557K0	DDB_G0273433	DDB_G0273433	TNF receptor-associated factor family protein DDB_G0273433/DDB_G0273509	1.37	0.000
112	Q55DC2	DDB_G0269706	DDB0190488	Uncharacterized protein	1.37	0.036
113	Q54QF3	DDB_G0283851	DDB0185734	DSPc domain-containing protein	1.36	0.006
114	Q54T01	DDB_G0282105	DDB_G0282105	Probable protein phosphatase	1.36	0.010
115	Q86KU7	DDB_G0277723	DDB0230057	Carboxylic ester hydrolase	1.36	0.036
116	P20609	DDB_G0277837	cxgE	Cytochrome c oxidase subunit 7e	1.36	0.000
117	Q867T7	DDB_G0288773	ncfA	NADPH oxidase activator	1.36	0.012
118	Q54Z26	DDB_G0277947	shmt1	Serine hydroxymethyltransferase 1	1.36	0.000
119	Q55BY2	DDB_G0270774	DDB_G0270774	DUF1084 domain-containing protein	1.35	0.026
120	Q54IT1	DDB_G0288537	DDB_G0288537	Putative uncharacterized protein	1.35	0.001
121	Q54QU8	DDB_G0283629	DDB_G0283629	Probable zinc transporter protein	1.35	0.009
122	Q55GM3	DDB_G0267610	DDB0189402	Epimerase domain-containing protein	1.35	0.000
123	Q54Q33	DDB_G0284165	DDB0218581	Ras-associating domain-containing protein	1.35	0.046
124	Q54UU9	DDB_G0280813	DDB0218256	Uncharacterized protein	1.35	0.006
125	Q556N3	DDB_G0272576	DDB0168021	Uncharacterized protein	1.35	0.026

126	Q23892	DDB_G0292810	gluA	Lysosomal beta glucosidase	1.35	0.000
127	Q54S87	DDB_G0282607	crsA	Ceramide synthase	1.35	0.004
128	P42521	DDB_G0274021	rnrB-1	Ribonucleoside-diphosphate reductase small subunit	1.34	0.000
129	Q54CR1	DDB_G0292746	DDB0184540	Uncharacterized protein	1.34	0.046
130	Q556Y3	DDB_G0273739	DDB0168100	SMP-LTD domain-containing protein	1.34	0.000
131	Q557E5	DDB_G0273387	DDB0217220	Uncharacterized protein	1.34	0.044
132	P34045	DDB_G0276455	gpaG	Guanine nucleotide-binding protein alpha-7 subunit	1.34	0.000
133	Q54K95	DDB_G0287515	tat	Tyrosine aminotransferase	1.34	0.000
134	Q54NH4	DDB_G0285257	DDB0186414	Fucokinase domain-containing protein	1.33	0.029
135	Q54Q60	DDB_G0284079	DDB0185830	Uncharacterized protein	1.33	0.001
136	Q54LR3	DDB_G0286447	DDB_G0286447	Uncharacterized protein	1.33	0.011
137	Q553U5	DDB_G0275179	ADA2	Adenosine deaminase 2	1.33	0.000
138	Q8MQU6	DDB_G0267426	cshA	Citrate synthase, peroxisomal	1.33	0.000
139	Q6TMJ9	DDB_G0280061	sybA	Synaptobrevin-A	1.33	0.020
140	Q8WQ85	DDB_G0288557	vilA	Villidin	1.33	0.000
141	Q86A16	DDB_G0272680	DDB0168928	PPM-type phosphatase domain-containing protein	1.33	0.000
142	Q54T13	DDB_G0282055	DDB0205121	Uncharacterized protein	1.33	0.002
143	Q559N8	DDB_G0272466	DDB0217020	Uncharacterized protein	1.33	0.008
144	Q86IF9	DDB_G0272678	DDB0168929	Uncharacterized protein	1.33	0.000
145	Q558Z0	DDB_G0272867	argS1	Probable arginine--tRNA ligase, cytoplasmic	1.33	0.000
146	P19198	DDB_G0272560	capA-1	cAMP-binding protein 1	1.33	0.002
147	Q55DZ2	DDB_G0269462	DDB_G0269462	Ubiquitin domain-containing protein	1.33	0.000
148	Q559P7	DDB_G0272460	DDB0217017	Uncharacterized protein	1.33	0.014
149	Q55DN6	DDB_G0269588	DDB0190384	Uncharacterized protein	1.32	0.001
150	Q54RB0	DDB_G0283261	acoA	Acyl-coenzyme A oxidase	1.32	0.000
151	Q94464	DDB_G0277849	dymA	Dynamin-A	1.32	0.000
152	Q86HU8	DDB_G0276473	DDB0167100	Epimerase domain-containing protein	1.32	0.000
153	Q556W8	DDB_G0273297	DDB0203045	Uncharacterized protein	1.32	0.042
154	Q86K42	DDB_G0277249	DDB0169141	ANK_REP_REGION domain-containing protein	1.32	0.024
155	Q86IF6	DDB_G0272989	DDB0217073	PX domain-containing protein	1.32	0.000
156	Q86B09	DDB_G0272781	pmmB	Phosphomannomutase 2	1.32	0.000
157	Q7KWM9	DDB_G0272738	iunH	Probable ribonucleoside hydrolase	1.32	0.000
158	Q54VC9	DDB_G0280445	DDB_G0280445	Glutathione-dependent formaldehyde-activating, GFA family protein	1.32	0.000
159	Q54QM1	DDB_G0283863	DDB0218526	Kinase	1.31	0.013
160	Q75JL8	DDB_G0276065	DDB0169464	Acyl-coenzyme A oxidase	1.31	0.000
161	Q55F21	DDB_G0268664	aatA	Aspartate aminotransferase, mitochondrial	1.31	0.000
162	Q553M5	DDB_G0275843	DDB_G0275843	Uncharacterized protein	1.31	0.003
163	Q75JH9	DDB_G0276103	DDB0169505	Fimbrin	1.31	0.002
164	Q550Y6	DDB_G0276739	DDB0202924	Uncharacterized protein	1.31	0.030
165	Q54SR8	DDB_G0282265	sgmC	Sphingomyelinase phosphodiesterase C	1.31	0.001
166	C7G025	DDB_G0295849	DDB_G0295849	RHOMBOID-like protein	1.31	0.015
167	P34044	DDB_G0283151	gpaF	Guanine nucleotide-binding protein alpha-6 subunit	1.31	0.004
168	Q556J2	DDB_G0272883	ksrA-1	3-ketodihydrosphingosine reductase	1.31	0.000
169	Q55A08	N/A	DDB0216977	Uncharacterized protein	1.31	0.017
170	Q54NZ6	DDB_G0285041	DDB_G0285041	Isochorismatase family protein 1A	1.31	0.002
171	Q551W4	DDB_G0276321	DDB0217779	Uncharacterized protein	1.31	0.000
172	Q558Y5	DDB_G0272997	DDB0217078	XRN_N domain-containing protein	1.31	0.033
173	Q550U0	DDB_G0277023	DDB0217867	Uncharacterized protein	1.30	0.008
174	P26310	DDB_G0282097	cxfA	Cytochrome c oxidase polypeptide 6, mitochondrial	1.30	0.003
175	Q55DA0	DDB_G0270826	abcG22	ABC transporter G family member 22	1.30	0.001
176	Q54RB1	DDB_G0283281	DDB0185428	SGL domain-containing protein	1.30	0.030
177	Q54GY8	DDB_G0289827	rab18	Ras-related protein Rab-18	1.30	0.001
178	Q54LW6	DDB_G0286463	DDB_G0286463	Uncharacterized protein	1.30	0.012
179	Q86A24	DDB_G0272708	DDB_G0272708	Protein-lysine N-methyltransferase	1.30	0.007
180	Q556S4	DDB_G0273861	DDB0168045	Uncharacterized protein	1.30	0.002

181	Q54L14	DDB_G0286977	DDB0218872	J domain-containing protein	1.30	0.005
182	Q76P07	DDB_G0277165	DDB_G0277165	Probable serine/threonine-protein kinase	1.30	0.001
183	Q54PU2	DDB_G0284291	gatc	Glutamyl-tRNA(Gln) amidotransferase subunit C, mitochondrial	1.30	0.037
184	Q54BM7	DDB_G0293524	ppkA	Polyphosphate kinase	1.30	0.000
185	Q55BH9	DDB_G0271342	dgat1	Diacylglycerol O-acyltransferase 1	1.29	0.027
186	Q7KWQ2	DDB_G0272660	serS	Serine--tRNA ligase, cytoplasmic	1.29	0.000
187	Q555V8	DDB_G0274811	argB	Acetylglutamate kinase	1.29	0.006
188	Q8T8M2	DDB_G0271142	proC	Profilin-3	1.29	0.022
189	Q556M4	DDB_G0272604	cyp508A2-1	Probable cytochrome P450 508A2	1.29	0.003
190	Q54IN6	DDB_G0288629	DDB_G0288629	Uncharacterized protein	1.29	0.002
191	Q54UA6	DDB_G0281185	DDB0204067	Uncharacterized protein	1.29	0.016
192	Q54TC2	DDB_G0281865	adh5	Alcohol dehydrogenase class-3	1.29	0.001
193	Q55C63	DDB_G0270208	DDB0190879	WD_REPEATS_REGION domain-containing protein	1.29	0.003
194	Q55GH4	DDB_G0267674	glcS	Glycogen [starch] synthase	1.29	0.000
195	C7G038	DDB_G0285797	DDB_G0285797	Uncharacterized protein	1.29	0.015
196	Q54Y32	DDB_G0278445	mpl3	MAP kinase phosphatase with leucine-rich repeats protein 3	1.29	0.002
197	Q54SZ2	DDB_G0282117	DDB0205143	MYND-type domain-containing protein	1.29	0.023
198	Q557H6	DDB_G0273555	DDB_G0273411	EFP_N domain-containing protein	1.29	0.001
199	Q1ZXI8	DDB_G0278651	manE	Alpha-mannosidase E	1.29	0.010
200	Q86L28	DDB_G0276837	DDB_G0276837	Uncharacterized protein	1.29	0.000
201	Q556P6	DDB_G0273917	DDB0217108	Uncharacterized protein	1.29	0.000
202	O21047	DDB_G0294048	nad4	NADH-ubiquinone oxidoreductase chain 4	1.29	0.030
203	Q54YX6	DDB_G0278025	DDB0204373	HP domain-containing protein	1.29	0.000
204	Q54T73	DDB_G0281967	DDB0205055	Uncharacterized protein	1.29	0.012
205	Q54QH7	DDB_G0283889	DDB0218541	Uncharacterized protein	1.28	0.002
206	Q54CR8	DDB_G0292758	mobB	MOB kinase activator-like 1 homolog B	1.28	0.001
207	Q54HI2	DDB_G0289423	DDB_G0289423	SPX and EXS domain-containing protein 4	1.28	0.003
208	Q554S6	DDB_G0274131	act17	Actin-17	1.28	0.004
209	Q54HR1	DDB_G0289285	DDB0188346	Uncharacterized protein	1.28	0.011
210	P34147	DDB_G0286555	racA	Rho-related protein racA	1.28	0.002
211	Q54U08	DDB_G0281401	DDB0205558	Phospholipid-transporting ATPase	1.28	0.012
212	Q869N2	DDB_G0276459	pakB	Serine/threonine-protein kinase pakB	1.28	0.004
213	Q54NB9	DDB_G0285401	DDB0218708	Uncharacterized protein	1.27	0.042
214	Q86HT1	DDB_G0274441	psiL	Protein psiL	1.27	0.021
215	Q54KL1	DDB_G0287279	DDB0187394	Uncharacterized protein	1.27	0.002
216	Q54YY7	DDB_G0278003	DDB0204362	Uncharacterized protein	1.27	0.032
217	Q7YSJ4	DDB_G0278581	psiF	Protein psiF	1.27	0.000
218	Q54HB4	DDB_G0289573	eny2	Transcription and mRNA export factor ENY2	1.27	0.000
219	Q9NA13	DDB_G0292564	iplA	Inositol 1,4,5-trisphosphate receptor-like protein A	1.27	0.003
220	Q556J9	DDB_G0272889	surf1-1	SURF1-like protein	1.27	0.003
221	Q54JA5	DDB_G0288195	sibC	Integrin beta-like protein C	1.27	0.003
222	Q54TB6	DDB_G0281875	DDB0204262	Corrinoid adenosyltransferase	1.27	0.001
223	Q54JX6	DDB_G0287749	DDB_G0287749	Uncharacterized protein	1.27	0.000
224	Q55E91	DDB_G0269342	DDB0190189	Uncharacterized protein	1.27	0.003
225	Q8MP23	DDB_G0274549	DDB_G0274549	Uncharacterized protein	1.27	0.011
226	Q9GPZ7	DDB_G0269224	fszB	Mitochondrial division protein fszB	1.27	0.002
227	Q54IZ7	DDB_G0288405	DDB0187927	Rab-GAP TBC domain-containing protein	1.27	0.018
228	Q54XS1	DDB_G0278781	pah	Phenylalanine-4-hydroxylase	1.27	0.001
229	Q54ST8	DDB_G0282287	DDB0218370	Uncharacterized protein	1.27	0.001
230	Q556K0	DDB_G0273999	DDB0167985	RWD domain-containing protein	1.26	0.000
231	Q86KF9	DDB_G0272522	sgkA	Sphingosine kinase A	1.26	0.000
232	Q86B10	DDB_G0272777	DDB0168975	Uncharacterized protein	1.26	0.022
233	Q54NL4	DDB_G0285175	vmp1	Vacuole membrane protein 1 homolog	1.26	0.021
234	Q556S5	DDB_G0295833	DDB0217341	Short-chain dehydrogenase/reductase family protein	1.26	0.001

235	Q8T674	DDB_G0267430	abcG20	ABC transporter G family member 20	1.26	0.019
236	Q23884	DDB_G0294040	mrpl11	60S ribosomal protein L11, mitochondrial	1.26	0.008
237	Q54DG1	DDB_G0292270	comG	Aldehyde dehydrogenase family 3 comG	1.26	0.000
238	Q557F3	DDB_G0273597	DDB0168168	Uncharacterized protein	1.25	0.004
239	Q54XG9	DDB_G0278975	DDB_G0278975	G8 domain-containing protein	1.25	0.000
240	P14196	DDB_G0284871	AAC11	AAC-rich mRNA clone AAC11 protein	1.25	0.000
241	P16051	DDB_G0276267	gpaB	Guanine nucleotide-binding protein alpha-2 subunit	1.25	0.000
242	O61125	DDB_G0284181	krsA	Serine/threonine-protein kinase 4 homolog A	1.25	0.001
243	Q552S7	DDB_G0275913	DDB_G0275913	Putative acetyltransferase	1.25	0.000
244	Q54GQ7	DDB_G0289991	DDB0188673	Uncharacterized protein	1.25	0.000
245	Q8MML5	DDB_G0274109	paxB	Paxillin-B	1.25	0.018
246	Q8T1G5	DDB_G0275121	alyC	Lysozyme C	1.25	0.035
247	Q86HX3	DDB_G0276419	DDB0167048	4HBT domain-containing protein	1.25	0.000
248	Q55AH8	DDB_G0271880	DDB0216932	Uncharacterized protein	1.25	0.003
249	Q54CL1	DDB_G0292874	DDB0191664	Nefa_Nip30_N domain-containing protein	1.25	0.003
250	Q556V1	DDB_G0273089	coq10-1	Coenzyme Q-binding protein COQ10, mitochondrial	1.25	0.000
251	Q55CZ2	DDB_G0269848	DDB0190609	PlsC domain-containing protein	1.25	0.006
252	Q54CT8	DDB_G0292730	adkB	Probable adenylate kinase B	1.25	0.005
253	Q54I15	DDB_G0289109	DDB0219409	Uncharacterized protein	1.25	0.032
254	Q54QV0	DDB_G0283625	mob2	MOB kinase activator-like 2	1.25	0.000
255	Q54SA1	DDB_G0282579	pldZ	Phospholipase D Z	1.25	0.000
256	Q54RS9	DDB_G0283057	DDB0218425	Rap-GAP domain-containing protein	1.25	0.008
257	C7G062	DDB_G0295753	DDB_G0295753	Mitochondrial substrate carrier family protein	1.24	0.000
258	Q553T0	DDB_G0275359	DDB0202574	SAM_MT_ERG6_SMT domain-containing protein	1.24	0.000
259	O77229	DDB_G0274595	catA	Catalase-A	1.24	0.000
260	Q5TJ57	DDB_G0269626	forE	Formin-E	1.24	0.007
261	Q556K7	DDB_G0272801	DDB0167992	Uncharacterized protein	1.24	0.010
262	Q54GN9	DDB_G0290029	DDB0220663	C2H2 type Zn-finger-containing protein	1.24	0.000
263	Q54WH5	DDB_G0279657	elmoE	ELMO domain-containing protein E	1.24	0.000
264	Q86B07	DDB_G0272785	DDB0168980	Saposin B-type domain-containing protein	1.24	0.019
265	Q54IT3	DDB_G0288541	maoA	Probable flavin-containing monoamine oxidase A	1.24	0.000
266	Q54VI7	DDB_G0280311	DDB0206508	Uncharacterized protein	1.24	0.029
267	Q54MA7	DDB_G0286073	DDB0186803	Uncharacterized protein	1.24	0.002
268	Q54PL7	DDB_G0284477	DDB0186024	Uncharacterized protein	1.24	0.007
269	P22887	DDB_G0273069	ndkC-1	Nucleoside diphosphate kinase, cytosolic	1.24	0.000
270	Q54GR0	DDB_G0289987	DDB0188671	Uncharacterized protein	1.24	0.000
271	Q54QY3	DDB_G0283541	yipf5	Protein YIPF5 homolog	1.24	0.036
272	Q86L51	DDB_G0272857	rapB	Ras-related protein rapB	1.23	0.002
273	C7FZZ9	DDB_G0295773	DDB_G0295773	DUF1077 family protein	1.23	0.005
274	Q86IL9	DDB_G0272196	DDB0168714	VWFA domain-containing protein	1.23	0.008
275	P54658	DDB_G0272819	hspC	32 kDa heat shock protein	1.23	0.002
276	Q557E6	DDB_G0273139	DDB0168161	DNA repair protein RAD51 homolog	1.23	0.000
277	Q37315	DDB_G0294016	atp9	ATP synthase subunit 9, mitochondrial	1.23	0.036
278	Q54Q34	DDB_G0349530	DDB0185859	Calponin-homology (CH) domain-containing protein	1.23	0.000
279	B0G176	DDB_G0295665	DDB_G0295665	Uncharacterized protein	1.23	0.008
280	Q54I22	DDB_G0289059	DDB_G0289059	Short-chain dehydrogenase/reductase family protein	1.23	0.000
281	Q54NE0	DDB_G0285329	DDB0186442	Uncharacterized protein	1.23	0.000
282	Q54QN4	DDB_G0283731	DDB_G0283731	Uncharacterized transmembrane protein	1.23	0.000
283	Q1ZXC8	DDB_G0289119	pXi	Probable serine/threonine-protein kinase pXi	1.23	0.002
284	Q54IS6	DDB_G0288549	rtf2	Replication termination factor 2	1.23	0.013
285	Q556T7	DDB_G0273831	DDB0168059	SEC7 domain-containing protein	1.23	0.000
286	Q55CK8	DDB_G0270018	DDB0190742	Uncharacterized protein	1.23	0.011
287	Q556X0	DDB_G0273075	DDB0168087	Uncharacterized protein	1.23	0.025
288	Q7KWQ0	DDB_G0272746	DDB0168237	Uncharacterized protein	1.23	0.011
289	Q55GW2	DDB_G0267484	acp1	Low molecular weight phosphotyrosine protein phosphatase	1.23	0.002

290	Q54PP1	DDB_G0284407	erg24	Delta(14)-sterol reductase	1.22	0.000
291	P54680	DDB_G0277855	fimA	Fimbrin	1.22	0.000
292	Q54QG2	DDB_G0283847	DDB0185725	Uncharacterized protein	1.22	0.000
293	Q1ZXQ4	DDB_G0269474	fcsB	Fatty acyl-CoA synthetase B	1.22	0.010
294	Q86IL5	DDB_G0272668	ppp4r2	Serine/threonine-protein phosphatase 4 regulatory subunit 2	1.22	0.000
295	Q54G10	DDB_G0290493	DDB0188911	Uncharacterized protein	1.22	0.020
296	Q55GP8	DDB_G0267568	gacO	Rho GTPase-activating protein gacO	1.22	0.001
297	Q54Y30	DDB_G0278449	rsmC	Small GTPase	1.22	0.013
298	Q54JJ8	DDB_G0288005	DDB0187731	PNPLA domain-containing protein	1.22	0.003
299	Q9BI24	DDB_G0272795	3B	Prespore-specific protein	1.22	0.010
300	Q556Y8	DDB_G0273729	DDB_G0273199	Probable rhodanese domain-containing dual specificity protein phosphatase	1.22	0.001
301	Q557E0	DDB_G0273623	hspE-1	Heat shock cognate 70 kDa protein 2	1.22	0.000
302	Q55CE0	DDB_G0270102	mkcF	Probable serine/threonine-protein kinase mkcF	1.22	0.001
303	Q552A1	DDB_G0276247	DDB_G0276247	Uncharacterized protein	1.22	0.000
304	Q557I4	DDB_G0273419	DDB0168194	Uncharacterized protein	1.22	0.013
305	Q86IY4	DDB_G0274339	DDB0167887	Uncharacterized protein	1.22	0.000
306	Q8MLZ3	DDB_G0274383	pdeD	cGMP-dependent 3',5'-cGMP phosphodiesterase A	1.22	0.014
307	Q86CR8	DDB_G0286191	atg8	Autophagy-related protein 8	1.22	0.005
308	Q559H0	DDB_G0272933	DDB_G0272933	SAYSvFN domain-containing protein	1.22	0.025
309	Q54J34	DDB_G0288333	purB	Adenylosuccinate lyase	1.22	0.000
310	Q8I7T3	DDB_G0288511	sadA	Substrate-adhesion molecule	1.22	0.001
311	Q54DY1	DDB_G0291916	argJ	Arginine biosynthesis bifunctional protein ArgJ, mitochondrial	1.21	0.000
312	P34136	DDB_G0276615	hmgB	3-hydroxy-3-methylglutaryl-coenzyme A reductase 2	1.21	0.000
313	Q54D70	DDB_G0292448	DDB0184405	Methyltransf_11 domain-containing protein	1.21	0.002
314	Q86K57	DDB_G0277231	DDB0169117	Uncharacterized protein	1.21	0.002
315	Q8T8P3	DDB_G0293194	abcD2	ABC transporter D family member 2	1.21	0.005
316	Q54FY7	DDB_G0290529	cpnE	Copine-E	1.21	0.001
317	Q54P77	DDB_G0284831	4cl1	Probable 4-coumarate--CoA ligase 1	1.21	0.000
318	Q9NKW1	DDB_G0291247	mfeA	Peroxisomal multifunctional enzyme A	1.21	0.000
319	Q55DC0	DDB_G0269708	DDB0190490	Uncharacterized protein	1.21	0.000
320	Q54EA0	DDB_G0291712	DDB0219687	Uncharacterized protein	1.21	0.008
321	Q54M18	DDB_G0286257	argC	Bifunctional protein argC, mitochondrial	1.21	0.000
322	Q86JC1	DDB_G0272666	DDB0168867	Putative ankyrin repeat protein	1.21	0.000
323	Q54RE9	DDB_G0283171	DDB0185393	Uncharacterized protein	1.21	0.037
324	Q54LW1	DDB_G0286383	DDB0186941	Uncharacterized protein	1.21	0.000
325	Q37313	DDB_G0294018	nad1	NADH-ubiquinone oxidoreductase chain 1	1.21	0.027
326	Q54W91	DDB_G0279821	DDB0206310	Uncharacterized protein	1.21	0.007
327	Q54LA5	DDB_G0286747	H2AZ	Histone H2A.z	1.21	0.015
328	Q54F26	DDB_G0291155	DDB0189279	RRM domain-containing protein	1.21	0.000
329	Q54S68	DDB_G0282641	DDB0204886	Uncharacterized protein	1.21	0.008
330	Q557I5	DDB_G0273537	DDB0217155	Uncharacterized protein	1.21	0.024
331	Q8IS19	DDB_G0289667	gefD	Ras guanine nucleotide exchange factor D	1.21	0.011
332	P10733	DDB_G0289327	sevA	Severin	1.21	0.001
333	O96923	DDB_G0291125	gnrA	Gelsolin-related protein of 125 kDa	1.21	0.004
334	Q1ZXQ0	DDB_G0270932	phr	Sca1 complex protein phr	1.21	0.003
335	Q54EU8	DDB_G0291324	DDB0183821	Uncharacterized protein	1.21	0.007
336	Q54TH9	DDB_G0281739	gacY	Rho GTPase-activating protein gacY	1.20	0.014
337	Q54R66	DDB_G0283365	polr2k	DNA-directed RNA polymerases I, II, and III subunit rpabc4	1.20	0.006
338	Q557B6	DDB_G0273237	DDB_G0273237	FAM172 family protein homolog	1.20	0.000
339	Q8T1P1	DDB_G0273453	DDB_G0273453	Uncharacterized protein DDB_G0273453/DDB_G0273565	1.20	0.041
340	O21048	DDB_G0294020	nad2	NADH-ubiquinone oxidoreductase chain 2	1.20	0.004
341	Q54VA3	DDB_G0280493	DDB0205236	Uncharacterized protein	1.20	0.049

342	Q54EM2	DDB_G0291454	DDB0183901	IPT/TIG domain-containing protein	1.20	0.015
343	Q54E45	DDB_G0291810	DDB0184078	Uncharacterized protein	1.20	0.000
344	Q54BK7	DDB_G0293584	DDB0192021	SE domain-containing protein	1.20	0.003
345	Q54ND4	DDB_G0285341	DDB0186448	Uncharacterized protein	1.20	0.015
346	Q86KI1	DDB_G0273439	ap2a1-1	AP-2 complex subunit alpha-2	1.20	0.001
347	Q54WU5	DDB_G0279429	DDB0205761	Uncharacterized protein	1.20	0.020

Down-regulated proteins in ATG9¯/16¯ versus AX2 cells

#	UniProt ID	DDB_G ID	GeneName	GeneProduct	FC	p-value
1	Q54ND3	DDB_G0285389	DDB_G0285389	Protein UXT homolog	0.40	0.001
2	Q7KWV8	DDB_G0272518	DDB0168832	Uncharacterized protein	0.40	0.000
3	Q95US4	DDB_G0279921	gp130	Lipid-anchored plasma membrane glycoprotein 130	0.41	0.000
4	Q54PL9	DDB_G0284547	DDB0218606	Uncharacterized protein	0.43	0.000
5	Q54FF8	DDB_G0290885	DDB0189134	Uncharacterized protein	0.45	0.018
6	Q54TC9	DDB_G0281821	sre1	Elongation of fatty acids protein sre1	0.46	0.000
7	Q54FF6	DDB_G0290887	DDB0189136	Uncharacterized protein	0.46	0.004
8	P54657	DDB_G0285793	cadA	Calcium-dependent cell adhesion molecule 1	0.47	0.000
9	P02886	DDB_G0273063	dscA-1	Discoidin-1 subunit A	0.47	0.001
10	P26199	DDB_G0287125	proA	Profilin-1	0.48	0.000
11	Q54G81	DDB_G0290325	DDB0188839	Peptidase C50 domain-containing protein	0.49	0.003
12	Q86HF8	DDB_G0271852	DDB0168536	Uncharacterized protein	0.50	0.017
13	Q54SY1	DDB_G0282149	DDB0205154	Uncharacterized protein	0.51	0.000
14	Q54LG3	DDB_G0286651	DDB_G0286651	Saposin B domain-containing protein	0.51	0.014
15	Q54IN0	DDB_G0288635	DDB0188033	Uncharacterized protein	0.51	0.001
16	C7FZY1	DDB_G0295807	DDB_G0295807	Short-chain dehydrogenase/reductase family protein	0.51	0.000
17	O15736	DDB_G0275323	tipD	Protein tipD (ATG16)	0.52	0.001
18	Q58A41	DDB_G0289467	DD8-14	G domain-containing protein	0.53	0.000
19	Q54PD4	DDB_G0284629	DDB_G0284629	SnoaL-like domain-containing protein	0.54	0.000
20	Q550I1	DDB_G0277097	DDB0217906	Endotoxin_N domain-containing protein	0.54	0.000
21	Q54EW2	DDB_G0291301	DDB_G0291301	Putative bifunctional amine oxidase	0.55	0.000
22	Q54P26	DDB_G0284859	samkB	Probable serine/threonine-protein kinase samkB	0.55	0.000
23	C7G078	DDB_G0295801	DDB_G0295801	B_lectin domain-containing protein	0.55	0.000
24	Q54IX4	DDB_G0288443	DDB0187950	Uncharacterized protein	0.57	0.000
25	Q7Z1I0	DDB_G0289813	cupF	Calcium up-regulated protein F	0.57	0.003
26	Q54SB4	DDB_G0282559	DDB0204837	Purple acid phosphatase	0.57	0.003
27	Q54FU0	DDB_G0290593	DDB0188978	ADF-H domain-containing protein	0.57	0.000
28	P54661	DDB_G0287587	smlA	Small aggregate formation protein	0.57	0.000
29	Q1ZXH5	DDB_G0279681	DDB_G0279681	Calcium-binding EF-hand domain-containing protein	0.57	0.040
30	P42530	DDB_G0292552	dscE	Discoidin-2	0.57	0.000
31	P16643	DDB_G0272562	csbB	Glycoprotein 24B	0.59	0.035
32	Q54QT0	DDB_G0283653	DDB0185606	Uncharacterized protein	0.59	0.002
33	Q54DI2	DDB_G0292236	DDB0184285	Uncharacterized protein	0.59	0.000
34	Q54WD8	DDB_G0279717	DDB0206002	Carboxylic ester hydrolase	0.60	0.000
35	Q54CY3	DDB_G0292636	DDB_G0292636	Putative uncharacterized protein	0.61	0.002
36	Q55EK6	DDB_G0268848	DDB0190082	Uncharacterized protein	0.61	0.000
37	C7G077	DDB_G0295799	DDB_G0295799	BB_PF domain-containing protein	0.62	0.002
38	Q54YR8	DDB_G0278115	DDB0204431	Uncharacterized protein	0.62	0.000
39	P13231	DDB_G0282141	hatA	Hisactophilin-1	0.62	0.000
40	Q54LX0	DDB_G0286371	cbpM	Calcium-binding protein M	0.62	0.000
41	Q54N59	DDB_G0285485	DDB0186526	Uncharacterized protein	0.62	0.001
42	Q55CE9	DDB_G0270088	DDB0190795	Uncharacterized protein	0.62	0.000
43	Q55GC4	DDB_G0267728	DDB0189497	Uncharacterized protein	0.63	0.001

44	P21837	DDB_G0285419	cryS	Crystal protein	0.63	0.000
45	P54670	DDB_G0277827	cafA	Calfumirin-1	0.63	0.001
46	Q54TN1	DDB_G0281653	DDB0204586	Uncharacterized protein	0.63	0.001
47	Q55C87	DDB_G0270166	DDB0190855	Uncharacterized protein	0.63	0.001
48	Q7Z1Z9	DDB_G0289883	cupG	Calcium up-regulated protein G	0.64	0.001
49	Q54KF7	DDB_G0287363	sibA	Integrin beta-like protein A	0.64	0.001
50	Q55EM4	DDB_G0268828	DDB0190067	Uncharacterized protein	0.64	0.001
51	Q55FN4	DDB_G0268026	DDB0189725	Uncharacterized protein	0.64	0.001
52	Q54JF0	DDB_G0288091	DDB0187779	Uncharacterized protein	0.65	0.000
53	P54653	DDB_G0267456	cbp2	Calcium-binding protein 2	0.65	0.000
54	P07670	DDB_G0276331	pyr4	Dihydroorotate dehydrogenase	0.65	0.000
55	Q55GC5	DDB_G0267726	DDB0189496	Uncharacterized protein	0.65	0.001
56	Q54HF4	DDB_G0289529	DDB0219440	AAA domain-containing protein	0.66	0.005
57	Q54TR1	DDB_G0281605	cfaD	Counting factor associated protein D	0.66	0.002
58	Q54RZ4	DDB_G0282815	orfSGP	BB_PF domain-containing protein	0.66	0.003
59	Q7KWW8	DDB_G0276795	ctbs1	Probable di-N-acetylchitobiase 1	0.66	0.002
60	Q54LY3	DDB_G0286307	DDB0186920	Uncharacterized protein	0.66	0.003
61	Q54Q84	DDB_G0284051	DDB0185804	Aa_trans domain-containing protein	0.67	0.000
62	Q55FI9	DDB_G0268090	DDB0189769	Glyco_hydro_18 domain-containing protein	0.67	0.000
63	Q54IS1	DDB_G0288563	DDB0187993	Pept_C1 domain-containing protein	0.67	0.003
64	Q54TD0	DDB_G0281823	V4-7	Peptidase S53 domain-containing protein	0.67	0.001
65	Q54IR0	DDB_G0288579	DDB0188003	Uncharacterized protein	0.67	0.003
66	Q54ZX6	DDB_G0277333	DDB0217941	Methyltransf_12 domain-containing protein	0.67	0.000
67	Q54FY1	DDB_G0290537	DDB0231475	Aldehyde dehydrogenase	0.67	0.000
68	Q54C63	DDB_G0293190	DDB0219831	FHA domain-containing protein	0.67	0.003
69	Q55EX9	DDB_G0268948	DDB_G0268948	Putative methyltransferase	0.67	0.000
70	Q54TQ9	DDB_G0281609	DDB0204558	Glyco_hydro_cc domain-containing protein	0.67	0.000
71	Q8SSU2	DDB_G0274223	DDB_G0274223	Glutathione S-transferase domain-containing protein	0.68	0.002
72	Q6B9X6	DDB_G0268144	vwkA	Alpha-protein kinase vwkA	0.68	0.000
73	Q55FK1	DDB_G0268070	DDB0189757	Uncharacterized protein	0.68	0.005
74	Q8MM62	DDB_G0276027	pdeE	cAMP/cGMP-dependent 3',5'-cAMP/cGMP phosphodiesterase B	0.68	0.023
75	Q54GQ2	DDB_G0290001	DDB0188678	Methyltransf_11 domain-containing protein	0.69	0.000
76	Q54NR1	DDB_G0285025	alrE	Aldo-keto reductase	0.69	0.001
77	Q54FW2	DDB_G0290659	DDB0219578	Uncharacterized protein	0.69	0.000
78	P54638	DDB_G0267380	argE	Acetylornithine deacetylase	0.69	0.000
79	Q869Z5	DDB_G0271892	DDB0231434	Putative glutathione S-transferase	0.69	0.001
80	Q54QE7	DDB_G0283915	DDB_G0283915	Esterase/lipase/thioesterase domain-containing protein	0.69	0.000
81	Q94465	DDB_G0288481	gchA	GTP cyclohydrolase 1	0.69	0.000
82	Q555N6	DDB_G0274705	DDB_G0274705	Glutathione S-transferase domain-containing protein	0.69	0.023
83	Q54RD5	DDB_G0283253	DDB_G0283253	Uncharacterized protein	0.70	0.000
84	Q54BI7	DDB_G0293604	DDB0192039	Uncharacterized protein	0.70	0.000
85	Q86IC8	DDB_G0275501	omt6	Probable caffeoyl-CoA O-methyltransferase 2	0.70	0.012
86	Q556W2	DDB_G0273781	abcG17-1	ABC transporter G family member 17	0.70	0.000
87	Q54YA0	DDB_G0278345	acly	Probable ATP-citrate synthase	0.70	0.000
88	Q95UC5	DDB_G0275693	AY055590	Dipeptidyl aminopeptidase	0.70	0.000
89	Q54C10	DDB_G0293274	DDB0215559	B box-type domain-containing protein	0.70	0.000
90	Q558X5	DDB_G0272756	csbC	Contact site B protein C	0.70	0.002
91	Q54FY2	DDB_G0290535	DDB0231474	Aldehyde dehydrogenase	0.70	0.000
92	Q54X99	DDB_G0279105	DDB0205038	Uncharacterized protein	0.71	0.014
93	Q55BN8	DDB_G0270814	nek2	Probable serine/threonine-protein kinase nek2	0.71	0.000
94	Q54BC2	DDB_G0293758	H2Bv1	Histone H2B.v1	0.71	0.025
95	Q54I60	DDB_G0288985	purN	Phosphoribosylglycinamide formyltransferase	0.71	0.000
96	Q7Z203	DDB_G0289815	cupB	Calcium up-regulated protein B	0.71	0.009
97	Q54BU4	DDB_G0293416	abcB1	ABC transporter B family member 1	0.71	0.000

98	Q55F82	DDB_G0268600	DDB_G0268600	Uncharacterized protein	0.71	0.016
99	Q86IX8	DDB_G0274329	DDB0167895	PPIase cyclophilin-type domain-containing protein	0.71	0.011
100	Q54J48	DDB_G0288305	pdx2	Probable pyridoxal 5'-phosphate synthase subunit pdx2	0.71	0.000
101	Q54UQ4	DDB_G0280893	rbsk	Ribokinase	0.71	0.000
102	Q86JM5	DDB_G0272012	DDB_G0272012	Putative elongation of fatty acids protein	0.71	0.001
103	Q55GE4	DDB_G0268504	DDB_G0268504	Uncharacterized protein	0.72	0.001
104	Q1ZXI0	DDB_G0279095	ptsA	6-pyruvoyl tetrahydrobiopterin synthase	0.72	0.000
105	Q54NT1	DDB_G0284999	DDB0186311	Uncharacterized protein	0.72	0.001
106	Q54JS9	DDB_G0287889	DDB0219276	Uncharacterized protein	0.72	0.000
107	Q54GK6	DDB_G0290091	rpl22a	60S ribosomal protein L22 2	0.72	0.006
108	Q54YA2	DDB_G0278341	DDB0205386	Uncharacterized protein	0.72	0.000
109	Q54UJ1	DDB_G0281049	DDB0203975	LIM zinc-binding domain-containing protein	0.72	0.001
110	Q54GJ1	DDB_G0290125	DDB0219527	Uncharacterized protein	0.72	0.008
111	B0G0Z5	DDB_G0269616	DDB_G0269616	Saposin B domain-containing protein	0.72	0.000
112	Q54GM4	DDB_G0290043	DDB0188706	B box-type domain-containing protein	0.72	0.001
113	Q54CQ7	DDB_G0292738	DDB0184536	NmrA domain-containing protein	0.72	0.000
114	Q54F04	DDB_G0291201	DDB0189300	AA_permease_C domain-containing protein	0.72	0.003
115	Q54W35	DDB_G0279909	lipA	Lipoxygenase domain-containing protein	0.72	0.001
116	Q54FB1	DDB_G0290959	rsc6	Random slug cDNA6 protein	0.72	0.000
117	Q54C73	DDB_G0293122	DDB_G0293122	Uncharacterized protein	0.72	0.001
118	Q54VI4	DDB_G0280317	gsta3	Putative glutathione S-transferase alpha-3	0.72	0.000
119	Q54MK3	DDB_G0285889	DDB0186733	Uncharacterized protein	0.73	0.000
120	Q54F74	DDB_G0291029	DDB0230064	Sulfate adenylyltransferase	0.73	0.000
121	Q54IQ3	DDB_G0288591	DDB0188011	Uncharacterized protein	0.73	0.001
122	Q55EN7	DDB_G0268810	DDB0190054	Cation_ATPase_N domain-containing protein	0.73	0.000
123	Q54M17	DDB_G0286259	DDB0186888	Uncharacterized protein	0.73	0.008
124	Q54RX1	DDB_G0282861	DDB_G0282861	Translationally-controlled tumor protein homolog 2	0.73	0.017
125	Q54BI6	DDB_G0293606	DDB0192040	CRAL-TRIO domain-containing protein	0.73	0.005
126	P34115	DDB_G0290257	GP138A	Cell surface glycoprotein gp138A	0.74	0.008
127	Q55AS0	N/A	DDB0203605	Uncharacterized protein	0.74	0.002
128	Q76P10	DDB_G0277367	DDB_G0277367	Uncharacterized protein	0.74	0.002
129	Q54CB3	DDB_G0293074	DDB0191762	Aa_trans domain-containing protein	0.74	0.048
130	P14330	DDB_G0280533	lmcB	Vegetative-specific protein V4	0.74	0.005
131	Q54UR0	DDB_G0280881	DDB_G0280881	Glutathione S-transferase domain-containing protein	0.74	0.006
132	Q55EG0	DDB_G0268916	DDB0190124	Uncharacterized protein	0.74	0.007
133	Q55DW4	DDB_G0269214	abcG1	ABC transporter G family member 1	0.74	0.003
134	Q54B48	DDB_G0293958	DDB_G0293958	Probable serine/threonine-protein kinase	0.74	0.002
135	Q54VT4	DDB_G0280147	ddx47	Probable ATP-dependent RNA helicase ddx47	0.74	0.006
136	Q75JW6	DDB_G0272184	DDB0168737	PKS_ER domain-containing protein	0.74	0.000
137	Q54DY7	DDB_G0291912	DDB_G0291912	Serine carboxypeptidase S10 family member 1	0.74	0.003
138	Q54GZ7	DDB_G0289849	DDB0216131	B box-type domain-containing protein	0.74	0.000
139	Q54XS6	DDB_G0278775	ube2c	Probable ubiquitin-conjugating enzyme E2 C	0.74	0.005
140	Q54EX7	DDB_G0291281	grxB	Glutaredoxin-like protein	0.74	0.019
141	Q54K57	DDB_G0287657	tpsA	Alpha,alpha-trehalose-phosphate synthase [UDP-forming] A	0.74	0.003
142	Q75JF7	DDB_G0276133	DDB0169527	Uncharacterized protein	0.74	0.026
143	Q54Z13	DDB_G0277971	atp6v1g	V-type proton ATPase subunit G	0.74	0.001
144	Q23921	DDB_G0289391	pkiA	Protein pkiA	0.75	0.000
145	Q55ED4	DDB_G0269284	DDB_G0269284	NKAP family protein	0.75	0.002
146	Q54TD6	DDB_G0281843	DDB0204241	Uncharacterized protein	0.75	0.009
147	Q54BX0	DDB_G0293366	DDB0191902	NLPC_P60 domain-containing protein	0.75	0.003
148	Q54WR3	DDB_G0279485	DDB0205795	Uncharacterized protein	0.75	0.010
149	Q54FT2	DDB_G0290603	DDB0188987	Uncharacterized protein	0.75	0.000
150	Q54WK0	DDB_G0279611	DDB0205936	Fatty acid hydroxylase domain-containing protein	0.75	0.003
151	Q86I40	DDB_G0275013	omt4	O-methyltransferase 4	0.75	0.047

152	Q54EU3	DDB_G0291334	DDB0183826	Uncharacterized protein	0.75	0.008
153	Q54K68	DDB_G0287555	DDB0231093	Cysteine dioxygenase	0.75	0.002
154	Q553P3	DDB_G0275385	DDB0202598	Fe2OG dioxygenase domain-containing protein	0.75	0.000
155	Q55G75	DDB_G0267786	DDB_G0267786	PH domain-containing protein	0.75	0.000
156	Q54W09	DDB_G0279973	DDB_G0279973	AhpC/TSA family protein	0.75	0.000
157	P34090	DDB_G0275007	cmfA	Conditioned medium factor	0.76	0.001
158	Q54R55	DDB_G0283401	DDB0185484	Pept_C1 domain-containing protein	0.76	0.014
159	Q54B49	DDB_G0293912	pks45	Probable polyketide synthase 45	0.76	0.001
160	Q55GW4	DDB_G0267482	DDB_G0267482	Putative uncharacterized protein	0.76	0.011
161	Q54QB9	DDB_G0283955	gacG	Rho GTPase-activating protein gacG	0.76	0.008
162	Q54DL7	DDB_G0292188	DDB_G0292188	von Willebrand factor A domain-containing protein	0.76	0.000
163	Q23917	DDB_G0284331	regA	3',5'-cyclic-nucleotide phosphodiesterase regA	0.76	0.002
164	Q54RY2	DDB_G0282841	DDB0204984	Uncharacterized protein	0.76	0.019
165	Q54LW3	DDB_G0286379	DDB_G0286379	Uncharacterized protein	0.76	0.002
166	Q54YX3	DDB_G0278031	DDB_G0278031	CRAL-TRIO domain-containing protein	0.76	0.001
167	Q54G77	DDB_G0290333	DDB0188843	Peptidase S53 domain-containing protein	0.76	0.001
168	Q86AQ3	DDB_G0277203	DDB0169112	3Beta_HSD domain-containing protein	0.76	0.002
169	Q54V71	DDB_G0280579	DDB_G0280579	Uncharacterized protein	0.76	0.037
170	Q54PD3	DDB_G0284631	DDB_G0284631	Uncharacterized protein	0.76	0.000
171	Q54UR2	DDB_G0280877	DDB0215228	Peptidylprolyl isomerase	0.76	0.003
172	Q55GG9	DDB_G0267680	DDB_G0267680	Uncharacterized protein	0.77	0.004
173	Q7KWS3	DDB_G0276299	agnA	Argonaut-like protein	0.77	0.000
174	P42526	DDB_G0282143	hatB	Hisactophilin-2	0.77	0.000
175	Q54DN6	DDB_G0292112	galK	Galactokinase	0.77	0.009
176	Q8T2R4	DDB_G0276927	DDB_G0276927	Uncharacterized protein	0.77	0.001
177	Q54C71	DDB_G0293124	kxcB	Kinase and exchange factor for Rac B	0.77	0.000
178	Q86LA5	DDB_G0272072	DDB0168637	B box-type domain-containing protein	0.77	0.009
179	Q55GC0	DDB_G0267734	DDB0189501	Methyltransf_11 domain-containing protein	0.77	0.000
180	Q54KN7	DDB_G0287227	trxE	Putative thioredoxin-5	0.77	0.000
181	P15808	DDB_G0280045	thyA	Flavin-dependent thymidylate synthase	0.77	0.000
182	Q55FT8	DDB_G0267958	DDB_G0267958	Bromo and FHA domain-containing protein	0.77	0.003
183	Q54F37	DDB_G0291187	DDB0219651	Peptidase C83 domain-containing protein	0.77	0.017
184	P24639	DDB_G0269160	nxnA	Annexin A7	0.77	0.000
185	Q54KM5	DDB_G0287257	DDB0231137	Uncharacterized protein (Fragment)	0.77	0.002
186	Q54J47	DDB_G0288299	pdx1	Probable pyridoxal 5'-phosphate synthase subunit pdx1	0.77	0.000
187	Q54EI4	DDB_G0291676	DDB0219676	Uncharacterized protein	0.77	0.031
188	Q86A02	DDB_G0275635	PIPkinA	Uncharacterized protein	0.77	0.047
189	B0G192	DDB_G0295669	DDB_G0295669	Isochorismatase hydrolase	0.77	0.000
190	Q54HV5	DDB_G0289201	DDB0188304	MFS domain-containing protein	0.77	0.002
191	Q54MZ6	DDB_G0285595	DDB0231295	Dihydroxybiphenyl dioxygenase domain-containing protein	0.77	0.001
192	Q54GP0	DDB_G0290027	DDB0188689	Uncharacterized protein	0.77	0.001
193	Q54EX9	DDB_G0291277	DDB0183789	Uncharacterized protein	0.78	0.003
194	Q54ZT0	DDB_G0277369	set	Protein set homolog	0.78	0.000
195	Q54H97	DDB_G0289609	DDB_G0289609	CBS domain-containing protein	0.78	0.002
196	P54679	DDB_G0282817	patB	Probable plasma membrane ATPase	0.78	0.002
197	Q55FY3	DDB_G0267908	DDB0189631	ABM domain-containing protein	0.78	0.028
198	Q55BP5	DDB_G0270418	top2	Probable DNA topoisomerase 2	0.78	0.000
199	Q55DP0	DDB_G0270566	DDB_G0270566	DDHD domain-containing protein	0.78	0.011
200	Q54M03	DDB_G0286341	DDB0231435	Putative glutathione S-transferase	0.78	0.000
201	Q55BA2	DDB_G0271242	DDB_G0271242	UPF0522 protein C	0.78	0.007
202	Q54GS2	DDB_G0289941	DDB0188659	Uncharacterized protein	0.78	0.001
203	Q54U46	DDB_G0281283	DDB0204129	N-acetyltransferase domain-containing protein	0.78	0.003
204	Q55AK6	DDB_G0271766	DDB0203625	Poly [ADP-ribose] polymerase	0.78	0.023
205	Q54LR8	DDB_G0286441	DDB0186987	Uncharacterized protein	0.78	0.003

206	Q54N91	DDB_G0285437	DDB0186492	Uncharacterized protein	0.78	0.008
207	Q54QF2	DDB_G0283901	DDB0218547	Cir_N domain-containing protein	0.78	0.010
208	Q54SA9	DDB_G0282565	DDB_G0282565	Uncharacterized protein	0.78	0.000
209	Q54PU9	DDB_G0284277	DDB0185943	Uncharacterized protein	0.78	0.038
210	Q54T06	DDB_G0282067	DDB_G0282067	Probable zinc transporter protein	0.78	0.048
211	P02599	DDB_G0279407	calA	Calmodulin	0.78	0.040
212	Q54TG6	DDB_G0281763	DDB0204655	HTH La-type RNA-binding domain-containing protein	0.78	0.000
213	Q54NS9	DDB_G0285003	aifA	Apoptosis-inducing factor homolog A	0.78	0.000
214	Q75K16	DDB_G0275459	DDB0231494	RNA-binding region RNP-1 domain-containing protein	0.78	0.000
215	Q54QK7	DDB_G0283789	dimt1	Probable dimethyladenosine transferase	0.78	0.003
216	Q555Q9	DDB_G0274105	glgB	1,4-alpha-glucan-branching enzyme	0.79	0.000
217	Q54PD6	DDB_G0284625	DDB0186106	EGF-like domain-containing protein	0.79	0.014
218	Q86H62	DDB_G0275555	glrx3	Glutaredoxin-3 homolog	0.79	0.000
219	Q54CW8	DDB_G0292652	DDB0184503	Uncharacterized protein	0.79	0.001
220	Q54QE2	DDB_G0283991	DDB0218552	Sulf_transp domain-containing protein	0.79	0.010
221	Q54SR9	DDB_G0282263	DDB0205303	Uncharacterized protein	0.79	0.017
222	Q55FF3	DDB_G0268138	gsta1	Putative glutathione S-transferase alpha-1	0.79	0.001
223	Q86HR3	DDB_G0274465	DDB0167672	DrsE domain-containing protein	0.79	0.000
224	Q54R16	DDB_G0283523	DDB_G0283523	UPF0538 protein	0.79	0.001
225	Q94503	DDB_G0279185	cprF	Cysteine proteinase 6	0.79	0.010
226	O76856	DDB_G0279411	ctsD	Cathepsin D	0.79	0.000
227	Q54BW7	DDB_G0293388	DDB_G0293388	ATP-dependent metalloprotease	0.79	0.007
228	Q54DT1	DDB_G0291980	abcA9	ABC transporter A family member 9	0.79	0.002
229	Q54G47	DDB_G0290409	DDB0188874	Uncharacterized protein	0.79	0.002
230	Q54PX3	DDB_G0284245	DDB0185922	Uncharacterized protein	0.79	0.002
231	Q55FM3	DDB_G0268042	DDB0189735	CS domain-containing protein	0.79	0.004
232	Q8T2H8	DDB_G0277405	DDB0216422	SAP DNA-binding domain-containing protein	0.80	0.002
233	Q54FV5	DDB_G0290577	DDB0188966	Uncharacterized protein	0.80	0.005
234	Q54VB1	DDB_G0280477	DDB0205225	Uncharacterized protein	0.80	0.033
235	Q869W6	DDB_G0275057	DDB_G0275057	Probable myosin light chain kinase	0.80	0.004
236	Q55C66	DDB_G0270204	crtp3	Crt homolog 3	0.80	0.000
237	Q54VW1	DDB_G0280105	DDB_G0280105	Serine carboxypeptidase S10 family member 2	0.80	0.002
238	Q54FV4	DDB_G0290645	DDB0216172	Uncharacterized protein	0.80	0.044
239	Q55D96	DDB_G0269734	DDB0190512	SPX domain-containing protein	0.80	0.017
240	Q54X86	DDB_G0279133	syn7B	Probable syntaxin-7B	0.80	0.001
241	Q54D21	DDB_G0292574	DDB0184451	Uncharacterized protein	0.80	0.034
242	Q869X7	DDB_G0275159	DDB_G0275159	Histone acetyltransferase type B catalytic subunit	0.80	0.004
243	Q54H41	DDB_G0289717	DDB0188538	B box-type domain-containing protein	0.80	0.037
244	Q55CF8	DDB_G0270070	DDB0190786	PLD phosphodiesterase domain-containing protein	0.80	0.000
245	Q55BP2	DDB_G0270420	DDB0191047	Uncharacterized protein	0.80	0.001
246	Q54YN2	DDB_G0278155	mai	Maleylacetoacetate isomerase	0.80	0.000
247	Q54G64	DDB_G0290377	agnB	Argonaut-like protein	0.80	0.005
248	Q54DV6	DDB_G0292012	DDB0184160	N-acetyltransferase domain-containing protein	0.80	0.043
249	Q55C60	DDB_G0270214	DDB0190882	F5/8 type C domain-containing protein	0.80	0.000
250	Q54HY5	DDB_G0289141	DDB_G0289141	Poly [ADP-ribose] polymerase	0.80	0.000
251	Q54VS1	DDB_G0280173	isca1	Iron-sulfur cluster assembly 1 homolog, mitochondrial	0.80	0.001
252	Q55DA6	DDB_G0269722	DDB_G0269722	Probable 18S rRNA (guanine-N(7))-methyltransferase	0.80	0.034
253	Q54BH5	DDB_G0293730	DDB_G0293730	PI-PLC X-box domain-containing protein	0.80	0.000
254	Q54QJ8	DDB_G0283871	DDB_G0283871	Uncharacterized protein	0.80	0.000
255	Q54LH3	DDB_G0286637	DDB0231509	3-methyl-2-oxobutanoate hydroxymethyltransferase	0.81	0.006
256	Q8MYE6	DDB_G0275953	DDB0169540	MOSC domain-containing protein	0.81	0.013
257	Q55CE1	DDB_G0270726	DDB0201947	Uncharacterized protein	0.81	0.028
258	Q8T5Z7	DDB_G0291994	abcA1	ABC transporter A family member 1	0.81	0.005
259	Q556S7	DDB_G0273113	DDB0168049	Uncharacterized protein	0.81	0.005
260	Q55F45	DDB_G0268642	DDB_G0268642	Probable serine/threonine-protein kinase	0.81	0.000
261	Q54MH9	DDB_G0285961	rfc1	Probable replication factor C subunit 1	0.81	0.007

262	Q54BD6	DDB_G0293740	ddx51	Probable ATP-dependent RNA helicase ddx51	0.81	0.016
263	Q556M8	DDB_G0272572	DDB0168017	Uncharacterized protein	0.81	0.006
264	Q75J89	DDB_G0277387	DDB0169194	Uncharacterized protein	0.81	0.003
265	P46794	DDB_G0267386	cysB	Cystathionine beta-synthase	0.81	0.000
266	Q559Q5	DDB_G0272458	DDB0217016	Uncharacterized protein	0.81	0.005
267	Q86K69	DDB_G0277189	DDB0169101	Ubiquitin-like domain-containing protein	0.81	0.001
268	Q8T850	DDB_G0275259	DDB0217609	Uncharacterized protein	0.81	0.012
269	Q54UD7	DDB_G0281139	DDB0204037	Uncharacterized protein	0.81	0.026
270	Q54KL2	DDB_G0287277	DDB0187393	Epimerase domain-containing protein	0.81	0.019
271	Q54XT8	DDB_G0278751	DDB_G0278751	Ribosomal RNA processing protein 36 homolog	0.81	0.020
272	Q75JC5	DDB_G0271820	DDB0216917	Uncharacterized protein	0.81	0.028
273	Q54U47	DDB_G0281281	DDB0204128	Uncharacterized protein	0.81	0.002
274	Q54FC3	DDB_G0290967	DDB0189173	Uncharacterized protein	0.81	0.002
275	Q54FY3	DDB_G0290479	hydA	Aldehyde dehydrogenase	0.81	0.001
276	Q54DZ1	DDB_G0291906	DDB0184128	UDENN domain-containing protein	0.81	0.005
277	Q54GX3	DDB_G0289839	DDB0188610	Lipase_3 domain-containing protein	0.82	0.011
278	Q54Q97	DDB_G0284011	DDB0218563	PlsC domain-containing protein	0.82	0.002
279	Q54CJ1	DDB_G0292978	DDB0219806	Uncharacterized protein	0.82	0.005
280	Q54RD2	DDB_G0283193	DDB0185407	U-box domain-containing protein	0.82	0.048
281	Q55AD2	DDB_G0271950	DDB_G0271950	B box-type domain-containing protein	0.82	0.009
282	Q55EC4	DDB_G0269304	DDB0190160	CS domain-containing protein	0.82	0.008
283	Q54M71	DDB_G0286147	pfdn6	Probable prefoldin subunit 6	0.82	0.013
284	Q54MM9	DDB_G0285851	DDB0186709	Uncharacterized protein	0.82	0.001
285	P54639	DDB_G0278721	cprD	Cysteine proteinase 4	0.82	0.031
286	Q54LT7	DDB_G0286419	cyp519E1	Probable cytochrome P450 519E1	0.82	0.032
287	Q55FA4	DDB_G0268198	rpa43	Probable DNA-directed RNA polymerase I subunit RPA43	0.82	0.005
288	Q54MI6	DDB_G0285919	tiprl	TIP41-like protein	0.82	0.000
289	Q54Z20	DDB_G0277959	cupJ	Putative calcium up-regulated protein J	0.82	0.046
290	Q95YL5	DDB_G0291081	pefA	Penta-EF hand domain-containing protein 1	0.82	0.003
291	Q54RM0	DDB_G0283075	DDB_G0283075	NuA4 complex subunit EAF3 homolog	0.82	0.043
292	Q54MN4	DDB_G0285827	DDB0186704	TPR_REGION domain-containing protein	0.82	0.000
293	Q54HX6	DDB_G0289151	mybI	Myb-like protein I	0.82	0.000
294	Q55F29	DDB_G0269048	DDB0216612	Hemerythrin domain-containing protein	0.82	0.000
295	Q551J8	DDB_G0276563	DDB0217814	Uncharacterized protein	0.82	0.007
296	Q54L23	DDB_G0286947	DDB0187205	Uncharacterized protein	0.82	0.001
297	Q54XR4	DDB_G0278793	DDB0206195	TPR_REGION domain-containing protein	0.82	0.000
298	Q54X77	DDB_G0279151	cenB	Centrin-B	0.82	0.006
299	Q8T690	DDB_G0287461	abcG3	ABC transporter G family member 3	0.82	0.003
300	Q55E90	DDB_G0269344	DDB0190190	WD_REPEATS_REGION domain-containing protein	0.82	0.007
301	Q54Y04	DDB_G0278491	DDB0205489	DTW domain-containing protein	0.82	0.007
302	Q55G18	DDB_G0267866	pomp	Proteasome maturation protein homolog	0.82	0.026
303	Q54NJ3	DDB_G0285215	DDB0186397	Uncharacterized protein	0.82	0.006
304	Q54B08	DDB_G0293992	DDB_G0293992	C2 domain-containing protein	0.82	0.019
305	Q54WT5	DDB_G0279449	DDB0205771	HP domain-containing protein	0.82	0.000
306	Q54UF7	DDB_G0281101	ldhA	Putative D-lactate dehydrogenase	0.82	0.001
307	Q9UA41	DDB_G0280401	cypD	Peptidyl-prolyl cis-trans isomerase D, mitochondrial	0.83	0.000
308	Q54IW8	DDB_G0288469	DDB0219344	RNB domain-containing protein	0.83	0.027
309	Q75JE2	DDB_G0271724	DDB0168502	Uncharacterized protein	0.83	0.000
310	Q55EK2	DDB_G0269016	cyp524A1	Probable cytochrome P450 524A1	0.83	0.000
311	Q55CB2	DDB_G0270134	DDB0190831	ADF-H domain-containing protein	0.83	0.001
312	Q54RK8	DDB_G0283097	DDB0185353	B box-type domain-containing protein	0.83	0.016
313	Q55D56	DDB_G0269784	DDB0190551	Nudix hydrolase domain-containing protein	0.83	0.019
314	Q54H42	DDB_G0289715	DDB0188537	Uncharacterized protein	0.83	0.013
315	Q54Y42	DDB_G0278427	DDB0205448	Protein-lysine N-methyltransferase	0.83	0.021

316	Q54YW8	DDB_G0278039	polr2h	DNA-directed RNA polymerases I, II, and III subunit rpabc3	0.83	0.001
317	Q54VI3	DDB_G0280319	glud2	Glutamate dehydrogenase 2	0.83	0.000
318	Q54YZ5	DDB_G0277989	DDB_G0277989	Probable serine/threonine-protein kinase	0.83	0.028
319	Q55D71	DDB_G0269764	cnrC	Putative countin receptor Cnr3	0.83	0.002
320	Q86HW2	DDB_G0349487	DDB0217812	Uncharacterized protein	0.83	0.033
321	Q551G5	DDB_G0276523	DDB0203882	O-acyltransferase	0.83	0.010
322	Q54QS2	DDB_G0283663	DDB0185614	Uncharacterized protein	0.83	0.000
323	Q54SR7	DDB_G0282267	fkbp2	FK506-binding protein 2	0.83	0.001
324	Q54WG1	DDB_G0279675	DDB0205980	CN hydrolase domain-containing protein	0.83	0.005
325	Q55E92	DDB_G0269340	DDB0190188	Uncharacterized protein	0.83	0.030
326	Q54SY2	DDB_G0282181	nvl	Putative ribosome biogenesis ATPase nvl	0.83	0.013
327	Q552P4	DDB_G0275937	DDB_G0275937	Uncharacterized protein	0.83	0.043
328	B0G106	DDB_G0272418	DDB_G0272418	Thioredoxin fold domain-containing protein	0.83	0.042
329	Q54GP8	DDB_G0290015	grxA	Glutaredoxin	0.83	0.034
330	Q6RZZ9	DDB_G0288361	kif13	Kinesin-related protein 13	0.83	0.001

Table S7. Common DEPs in ATG9¯, ATG16¯ and ATG9¯/16¯ cells.

	UniProt ID	dictyBase ID or Gene Name	ATG9¯		ATG16¯		ATG9¯/16¯	
			FC	*p*-value	FC	*p*-value	FC	*p*-value
Up	Q54RB1	DDB_G0283281	1.36	0.016	1.48	0.015	1.30	0.030
	P19198	capA-1	1.21	0.007	1.36	0.017	1.33	0.017
	Q8T849	sigI	1.70	0.000	1.27	0.001	1.39	0.000
	Q8MQU6	cshA	1.28	0.000	1.29	0.000	1.39	0.000
	Q54M11	DDB_G0286271	1.32	0.003	1.63	0.000	1.78	0.000
	Q559N8	DDB_G0272466	1.22	0.012	1.27	0.007	1.33	0.008
	Q54WE3	iliP	1.23	0.007	1.35	0.002	1.80	0.001
Down	P54657	cadA	0.65	0.000	0.64	0.000	0.47	0.000
	Q54SY2	nvl	0.82	0.001	0.77	0.004	0.83	0.013
	Q54PD4	DDB_G0284629	0.82	0.000	0.70	0.000	0.54	0.000
	Q54SB4	dduA	0.73	0.006	0.67	0.003	0.57	0.003
	Q54YR8	netD	0.72	0.001	0.63	0.000	0.62	0.000
	Q54PD3	DDB_G0284631	0.75	0.000	0.78	0.000	0.76	0.000
	Q55G75	DDB_G0267786	0.81	0.000	0.75	0.000	0.75	0.000
	Q54TC9	eloB	0.80	0.002	0.60	0.000	0.46	0.000
	Q54YA0	acly	0.83	0.000	0.68	0.000	0.70	0.000
	Q54G81	espl1	0.51	0.003	0.51	0.006	0.49	0.003
	Q95US4	gp130	0.68	0.000	0.55	0.000	0.41	0.000
	Q86JM5	DDB_G0272012	0.75	0.021	0.61	0.000	0.71	0.001
	Q54QT0	DDB_G0283653	0.72	0.011	0.79	0.018	0.59	0.002
	P15808	thyA	0.83	0.000	0.66	0.000	0.77	0.000

cap, cAMP-binding protein; sig, srfA-induced gene; csh, citrate synthase homology; ili, induced after Legionella infection; cad, calcium-dependent adhesion; nvl, nuclear valosin-containing like protein; ddu, downregulated in dupA mutant; net, nuclear envelope transmembrane; elo, elongase; acl, ATP citrate lyase; espl1, extra spindle poles-like 1; gp130, glycoprotein 130; thy, thymidylate synthase. FC, fold change. Fold change is rounded at two places after the decimal point.

Table S8. Oppositely regulated DEPs in ATG9^{-}, ATG16^{-} and/or ATG9^{-}/16^{-} cells.

UniProt ID	dictyBase ID or Gene Name	ATG9^{-}		ATG16^{-}		ATG9^{-}/16^{-}	
		FC	*p*-value	FC	*p*-value	FC	*p*-value
P14330	lmcB	1.31	0.002	1.38	0.000	0.74	0.005
Q54DL7	DDB_G0292188	1.68	0.000	1.23	0.000	0.76	0.000
Q54G64	agnB	1.49	0.000	1.21	0.000	0.80	0.005
Q54NR1	alrE	1.26	0.000	1.42	0.000	0.69	0.001
Q54NT1	DDB_G0284999	1.82	0.000	1.54	0.000	0.72	0.001
Q552P4	DDB_G0275937	1.22	0.007	1.53	0.018	0.83	0.043
Q55FN4	DDB_G0268026	1.33	0.004	1.24	0.000	0.64	0.001
O77257	p17	0.80	0.004	0.74	0.001	1.43	0.000
Q54RI2	DDB_G0283127	0.81	0.012	0.74	0.021	1.71	0.016
Q54X38	DDB_G0279229	0.71	0.001	0.73	0.002	1.96	0.001
Q54X05	DDB_G0279397	1.32	0.006	0.75	0.017	1.62	0.003
P02886	dscA-1	0.71	0.002	1.21	0.025	0.47	0.001
Q54CW8	DDB_G0292652	1.44	0.001			0.79	0.001
Q54D21	DDB_G0292574	1.24	0.004			0.80	0.034
Q54RK8	DDB_G0283097	1.22	0.001			0.83	0.016
Q54WD8	DDB_G0279717	1.29	0.002			0.60	0.000
Q54PU9	DDB_G0284277	1.46	0.023			0.78	0.038
Q86I40	omt4	1.20	0.034			0.75	0.047
Q6B9X6	vwkA	1.34	0.000			0.68	0.000
P54670	cafA			1.38	0.000	0.63	0.001
Q54BC2	H2Bv1			1.20	0.019	0.71	0.025
Q54BI7	DDB_G0293604			1.41	0.000	0.70	0.000
Q54CQ7	DDB_G0292738			1.65	0.000	0.72	0.000
Q54DI2	DDB_G0292236			1.38	0.007	0.59	0.000
Q54H97	DDB_G0289609			1.24	0.001	0.78	0.002
Q54IQ3	DDB_G0288591			1.60	0.000	0.73	0.001
Q54QE7	DDB_G0283915			1.20	0.000	0.69	0.000
Q54RZ4	DDB_G0282815			1.61	0.007	0.66	0.003
Q54YN2	mai			1.32	0.000	0.80	0.000
Q54SA9	DDB_G0282565			1.24	0.001	0.78	0.000
Q54TD0	tpp1F			1.24	0.000	0.67	0.001
Q54U46	DDB_G0281283			1.22	0.000	0.78	0.003
Q54VI4	gsta3			1.22	0.000	0.72	0.000
Q54VS1	isca1			1.20	0.003	0.80	0.001
Q54W09	DDB_G0279973			1.21	0.001	0.75	0.000
Q55AD2	DDB_G0271950			1.23	0.000	0.82	0.009
Q55CE9	DDB_G0270088			1.24	0.000	0.62	0.000

Q55EM4	DDB_G0268828			1.21	0.004	0.64	0.001
Q55EX9	DDB_G0268948			1.34	0.000	0.67	0.000
Q55F82	uduB			1.45	0.015	0.71	0.016
Q55GC4	uduA3			3.09	0.000	0.63	0.001
Q55GC5	uduA1			2.37	0.000	0.65	0.001
Q869Z5	gst4			1.24	0.004	0.69	0.001
Q86AQ3	DDB_G0277203			1.25	0.001	0.76	0.002
Q86HW2	DDB_G0349487			1.29	0.017	0.83	0.033
Q94503	cprF			1.30	0.001	0.79	0.010
O21042	cox1/2			0.78	0.001	1.45	0.002
O21049	cox3			0.82	0.000	1.43	0.000
P20609	cxgE			0.75	0.000	1.36	0.000
P26310	cxfA			0.77	0.000	1.30	0.003
Q1ZXP2	DDB_G0294587			0.77	0.030	1.46	0.003
Q54CL1	DDB_G0292874			0.72	0.007	1.25	0.003
Q54FV6	DDB_G0290575			0.83	0.002	1.41	0.002
Q54I40	DDB_G0289027			0.78	0.007	1.39	0.000
Q54J34	purB			0.83	0.000	1.22	0.000
Q54LB9	ponC5			0.65	0.007	5.07	0.001
Q54LW6	DDB_G0286463			0.71	0.016	1.30	0.012
Q54Q60	DDB_G0284079			0.83	0.008	1.33	0.001
Q54QF3	DDB_G0283851			0.83	0.007	1.36	0.006
Q54SA1	pldZ			0.83	0.009	1.25	0.000
Q54W91	DDB_G0279821			0.82	0.012	1.21	0.007
Q55GW2	acp1			0.82	0.007	1.23	0.002
Q76P07	DDB_G0277165			0.76	0.002	1.30	0.001
Q86AV9	pldG			0.76	0.009	2.81	0.002
Q86JN6	DDB_G0271992			0.77	0.005	1.45	0.017
Q8MML5	paxB			0.70	0.004	1.25	0.018

Red, increased; green, decreased. FC, Fold change; Fold change is rounded at two places after the decimal point.

Table S9. Peptide sequences used for PRM.

UniProt ID	dictyBase ID or Gene Name	Peptide Sequence	Precursor Mz	Precursor Charge	Product Mz	Product Charge	Fragment Ion
Q559N8	DDB_G0272466	HLEQFLNENEK	700,843873	2	1263,621558	1	y10
			700,843873	2	893,436323	1	y7
			700,843873	2	633,283845	1	y5
Q54YR8	netD	ADTIVHSDDIDNLVGK	856,428498	2	1075,526595	1	y10
			856,428498	2	416,286745	1	y4
			856,428498	2	204,134267	1	y2
Q54PD4	DDB_G0284629	ENLLDIIK	479,2844	2	601,391939	1	y5
			479,2844	2	488,307875	1	y4
			479,2844	2	260,196868	1	y2
Q54M11	DDB_G0286271	TLFEEYTK	515,758215	2	816,377411	1	y6
			515,758215	2	669,308997	1	y5
			515,758215	2	411,223811	1	y3
Q8T849	sigI	VVEYIQNHIQNINGLDK	999,026171	2	1265,659674	1	y11
			999,026171	2	1151,616747	1	y10
			999,026171	2	546,288202	1	y5
		VLSLPVYDTK	567,823889	2	835,455996	1	y7
			567,823889	2	526,250754	1	y4
			567,823889	2	363,187425	1	y3
		NQNIDIFGK	524,774731	2	464,286745	1	y4
			524,774731	2	351,202681	1	y3
			524,774731	2	204,134267	1	y2
Q54DL7	DDB_G0292188	QNLVFGEPYEETIK	833,919777	2	1212,578296	1	y10
			833,919777	2	1065,509882	1	y9
			833,919777	2	879,445825	1	y7
		ESGTVLFESAR	598,301309	2	821,451579	1	y7
			598,301309	2	722,383165	1	y6
			598,301309	2	333,188094	1	y3
		SFDNSDSIK	506,732728	2	778,357738	1	y7
			506,732728	2	663,330795	1	y6
			506,732728	2	347,228896	1	y3
HEAVY_PEPTIDES_PRTC		SAAGAFGPELSR	586,800329	2	815,428548	1	y7
			586,800329	2	668,360134	1	y6
			586,800329	2	611,33867	1	y5

Table S10. Gene Ontology statistical overrepresentation test results for DEPs using biological process, cellular component and molecular function annotation sets. GO analysis was performed with PANTHER 15.0 (released 2020-02-14) using DEPs with FC ≥ 1.20 or FC ≤ 0.83 and p-value ≤ 0.05 as input. Shading indicates related classes in an ontology. Sorting is done by the most specific subclass first, with its parent terms indented directly below it.

Biological Process – up-regulated proteins

**ATG9¯**	**# *Dictyostelium***	**# Experiment**	**Expected**	**Fold Enrichment**	**P value**	**FDR**
No statistically significant results						
**ATG16¯**	**# *Dictyostelium***	**# Experiment**	**Expected**	**Fold Enrichment**	**P value**	**FDR**
No statistically significant results						
**ATG9¯/16¯**	**# *Dictyostelium***	**# Experiment**	**Expected**	**Fold Enrichment**	**P value**	**FDR**
arginine biosynthetic process	5	3	.14	21.86	9.57E-04	4.23E-02
oxoacid metabolic process	336	21	9.22	2.28	6.70E-04	3.64E-02
organic acid metabolic process	362	22	9.93	2.21	9.63E-04	4.21E-02
metabolic process	3721	133	102.12	1.30	5.30E-04	3.07E-02
cellular process	5017	193	137.69	1.40	4.23E-09	3.14E-06
small molecule metabolic process	640	41	17.56	2.33	1.00E-06	2.98E-04
organic substance biosynthetic process	1018	47	27.94	1.68	6.65E-04	3.66E-02
biosynthetic process	1046	48	28.71	1.67	5.61E-04	3.20E-02
small molecule biosynthetic process	200	16	5.49	2.92	2.28E-04	1.59E-02
fatty acid beta-oxidation using acyl-CoA oxidase	9	4	.25	16.19	2.95E-04	1.88E-02
fatty acid beta-oxidation	23	6	.63	9.51	9.82E-05	8.26E-03
fatty acid catabolic process	30	6	.82	7.29	3.44E-04	2.13E-02
cellular lipid catabolic process	58	9	1.59	5.65	6.81E-05	7.06E-03
cellular lipid metabolic process	317	24	8.70	2.76	1.53E-05	2.43E-03
lipid metabolic process	382	33	10.48	3.15	2.08E-08	1.03E-05
lipid catabolic process	81	13	2.22	5.85	1.20E-06	2.83E-04
monocarboxylic acid catabolic process	37	6	1.02	5.91	9.20E-04	4.19E-02
small molecule catabolic process	142	13	3.90	3.34	2.64E-04	1.76E-02
fatty acid metabolic process	81	9	2.22	4.05	6.57E-04	3.66E-02
fatty acid oxidation	25	6	.69	8.75	1.46E-04	1.10E-02
lipid oxidation	27	6	.74	8.10	2.09E-04	1.50E-02
oxidation-reduction process	563	38	15.45	2.46	7.54E-07	2.40E-04
lipid homeostasis	13	4	.36	11.21	9.04E-04	4.20E-02
regulation of biological quality	361	23	9.91	2.32	2.90E-04	1.88E-02
biological regulation	1825	85	50.09	1.70	1.04E-06	2.74E-04
actin filament bundle assembly	34	8	.93	8.57	1.28E-05	2.11E-03
cellular component organization	1229	57	33.73	1.69	1.16E-04	9.24E-03
cellular component organization or biogenesis	1388	60	38.09	1.58	5.05E-04	2.96E-02
actin filament bundle organization	35	8	.96	8.33	1.53E-05	2.35E-03
actin filament organization	150	22	4.12	5.34	1.09E-09	1.62E-06
supramolecular fiber organization	180	22	4.94	4.45	2.24E-08	1.00E-05
actin cytoskeleton organization	204	25	5.60	4.47	2.29E-09	2.55E-06
cytoskeleton organization	293	27	8.04	3.36	1.29E-07	4.43E-05
organelle organization	922	49	25.30	1.94	1.64E-05	2.44E-03
actin filament-based process	205	25	5.63	4.44	2.50E-09	2.23E-06

ATG9⁻/16⁻ continued	# *Dictyostelium*	# Experiment	Expected	Fold Enrichment	P value	FDR
adenylate cyclase-modulating G protein-coupled receptor signaling pathway	23	5	.63	7.92	7.82E-04	3.83E-02
G protein-coupled receptor signaling pathway, coupled to cyclic nucleotide second messenger	23	5	.63	7.92	7.82E-04	3.79E-02
signal transduction	504	34	13.83	2.46	3.17E-06	7.06E-04
signaling	509	34	13.97	2.43	3.76E-06	7.28E-04
cell communication	617	43	16.93	2.54	6.23E-08	2.32E-05
cellular response to stimulus	985	53	27.03	1.96	4.47E-06	7.97E-04
response to stimulus	1282	72	35.18	2.05	1.05E-08	5.84E-06
regulation of cellular process	1452	66	39.85	1.66	5.94E-05	6.46E-03
regulation of biological process	1609	71	44.16	1.61	6.44E-05	6.83E-03
regulation of phagocytosis	31	6	.85	7.05	4.01E-04	2.42E-02
regulation of vesicle-mediated transport	69	9	1.89	4.75	2.24E-04	1.59E-02
macroautophagy	37	6	1.02	5.91	9.20E-04	4.23E-02
actin polymerization or depolymerization	58	9	1.59	5.65	6.81E-05	6.90E-03
negative regulation of organelle organization	49	7	1.34	5.21	6.87E-04	3.69E-02
negative regulation of cellular component organization	68	8	1.87	4.29	9.27E-04	4.17E-02
regulation of cellular component organization	248	19	6.81	2.79	1.03E-04	8.47E-03
regulation of organelle organization	202	16	5.54	2.89	2.53E-04	1.73E-02
negative regulation of response to stimulus	52	7	1.43	4.91	9.44E-04	4.21E-02
sorocarp morphogenesis	85	11	2.33	4.72	4.89E-05	5.45E-03
anatomical structure morphogenesis	176	15	4.83	3.11	1.89E-04	1.40E-02
anatomical structure development	550	32	15.09	2.12	1.03E-04	8.36E-03
developmental process	566	32	15.53	2.06	2.08E-04	1.52E-02
sorocarp development	451	29	12.38	2.34	3.78E-05	4.68E-03
non-reproductive fruiting body development	451	29	12.38	2.34	3.78E-05	4.43E-03
socially cooperative development	451	29	12.38	2.34	3.78E-05	4.81E-03
intraspecies interaction between organisms	451	29	12.38	2.34	3.78E-05	4.55E-03
regulation of actin polymerization or depolymerization	83	9	2.28	3.95	7.71E-04	3.95E-02
regulation of actin filament length	83	9	2.28	3.95	7.71E-04	3.82E-02
regulation of actin cytoskeleton organization	117	13	3.21	4.05	4.41E-05	5.04E-03
regulation of cytoskeleton organization	126	13	3.46	3.76	8.85E-05	7.73E-03
regulation of actin filament-based process	118	14	3.24	4.32	1.15E-05	1.97E-03
regulation of cellular component size	96	11	2.63	4.18	1.32E-04	1.03E-02
regulation of anatomical structure size	96	11	2.63	4.18	1.32E-04	1.02E-02
regulation of actin filament organization	83	9	2.28	3.95	7.71E-04	3.91E-02
protein dephosphorylation	83	9	2.28	3.95	7.71E-04	3.86E-02
phosphate-containing compound metabolic process	789	46	21.65	2.12	3.37E-06	7.15E-04
phosphorus metabolic process	799	46	21.93	2.10	3.92E-06	7.27E-04
chemotaxis to cAMP	97	10	2.66	3.76	5.74E-04	3.24E-02
chemotaxis	155	17	4.25	4.00	3.53E-06	7.16E-04
taxis	176	19	4.83	3.93	1.17E-06	2.90E-04
locomotion	252	26	6.92	3.76	2.82E-08	1.14E-05
response to external stimulus	386	34	10.59	3.21	8.01E-09	5.10E-06
response to chemical	435	32	11.94	2.68	1.01E-06	2.81E-04
response to organic cyclic compound	101	10	2.77	3.61	7.66E-04	4.02E-02
regulation of intracellular signal transduction	126	12	3.46	3.47	3.25E-04	2.04E-02
regulation of signal transduction	163	13	4.47	2.91	8.91E-04	4.18E-02
cell motility	106	10	2.91	3.44	1.08E-03	4.62E-02
localization of cell	106	10	2.91	3.44	1.08E-03	4.67E-02
localization	1321	59	36.25	1.63	2.63E-04	1.78E-02
phagocytosis	120	11	3.29	3.34	7.68E-04	3.98E-02

**ATG9^{-}/16^{-} continued**	**# *Dictyostelium***	**# Experiment**	**Expected**	**Fold Enrichment**	**P value**	**FDR**
aggregation involved in sorocarp development	132	12	3.62	3.31	4.80E-04	2.85E-02
cellular response to starvation	160	15	4.39	3.42	7.08E-05	6.86E-03
cellular response to nutrient levels	162	15	4.45	3.37	8.06E-05	7.48E-03
response to nutrient levels	183	17	5.02	3.38	2.62E-05	3.54E-03
response to extracellular stimulus	183	17	5.02	3.38	2.62E-05	3.44E-03
cellular response to extracellular stimulus	162	15	4.45	3.37	8.06E-05	7.33E-03
cellular response to external stimulus	163	15	4.47	3.35	8.59E-05	7.66E-03
response to starvation	165	15	4.53	3.31	9.75E-05	8.35E-03
positive regulation of catalytic activity	220	18	6.04	2.98	7.17E-05	6.80E-03
positive regulation of molecular function	240	19	6.59	2.88	6.84E-05	6.78E-03
regulation of molecular function	371	26	10.18	2.55	2.38E-05	3.31E-03
regulation of catalytic activity	343	25	9.41	2.66	1.85E-05	2.67E-03
regulation of GTPase activity	160	13	4.39	2.96	7.59E-04	4.03E-02
regulation of hydrolase activity	210	16	5.76	2.78	3.79E-04	2.31E-02
lipid biosynthetic process	182	14	4.99	2.80	7.95E-04	3.81E-02
Unclassified	5351	90	146.85	.61	4.67E-10	2.08E-06
RNA processing	483	2	13.26	.15	2.77E-04	1.82E-02
nucleic acid metabolic process	1025	12	28.13	.43	8.34E-04	3.95E-02

Biological Process – down-regulated proteins

ATG9¯	# *Dictyostelium*	# Experiment	Expected	Fold Enrichment	P value	FDR
fatty acid elongation, saturated fatty acid	7	3	.02	> 100	3.70E-06	8.25E-03
fatty acid elongation	10	3	.03	92.52	8.76E-06	7.81E-03
fatty acid elongation, polyunsaturated fatty acid	7	3	.02	> 100	3.70E-06	5.50E-03
fatty acid elongation, unsaturated fatty acid	7	3	.02	> 100	3.70E-06	1.65E-02
fatty acid elongation, monounsaturated fatty acid	7	3	.02	> 100	3.70E-06	4.13E-03
very long-chain fatty acid biosynthetic process	10	3	.03	92.52	8.76E-06	6.51E-03
very long-chain fatty acid metabolic process	10	3	.03	92.52	8.76E-06	5.58E-03
membrane lipid biosynthetic process	55	4	.18	22.43	3.76E-05	2.09E-02
**ATG16¯**	**# *Dictyostelium***	**# Experiment**	**Expected**	**Fold Enrichment**	**P value**	**FDR**
fatty acid elongation, saturated fatty acid	7	3	.11	28.08	3.71E-04	5.52E-02
carboxylic acid biosynthetic process	100	8	1.53	5.24	2.20E-04	4.91E-02
organic acid biosynthetic process	101	8	1.54	5.19	2.35E-04	4.98E-02
small molecule biosynthetic process	200	11	3.05	3.60	3.48E-04	5.74E-02
fatty acid elongation, polyunsaturated fatty acid	7	3	.11	28.08	3.71E-04	5.34E-02
fatty acid elongation, unsaturated fatty acid	7	3	.11	28.08	3.71E-04	5.71E-02
fatty acid elongation, monounsaturated fatty acid	7	3	.11	28.08	3.71E-04	5.17E-02
phosphatidylinositol catabolic process	7	3	.11	28.08	3.71E-04	5.02E-02
phosphorus metabolic process	799	27	12.20	2.21	1.45E-04	4.30E-02
phosphate-containing compound metabolic process	789	27	12.04	2.24	1.30E-04	4.14E-02
phospholipid catabolic process	14	4	.21	18.72	1.28E-04	4.41E-02
organophosphate catabolic process	32	5	.49	10.24	2.15E-04	5.05E-02
phosphatidylethanolamine catabolic process	7	3	.11	28.08	3.71E-04	4.87E-02
pyrimidine nucleoside monophosphate biosynthetic process	10	4	.15	26.21	4.41E-05	2.18E-02
pyrimidine nucleoside monophosphate metabolic process	10	4	.15	26.21	4.41E-05	1.96E-02
nucleoside monophosphate metabolic process	40	8	.61	13.10	5.09E-07	1.13E-03
nucleoside monophosphate biosynthetic process	28	8	.43	18.72	4.76E-08	2.12E-04
nucleoside phosphate biosynthetic process	99	9	1.51	5.96	3.54E-05	1.97E-02
nucleobase biosynthetic process	12	4	.18	21.84	7.82E-05	3.17E-02
nucleobase metabolic process	30	5	.46	10.92	1.64E-04	4.30E-02
purine ribonucleoside monophosphate biosynthetic process	14	4	.21	18.72	1.28E-04	4.77E-02
purine nucleoside monophosphate biosynthetic process	15	4	.23	17.47	1.61E-04	4.48E-02
purine nucleoside monophosphate metabolic process	18	4	.27	14.56	2.93E-04	5.44E-02
purine ribonucleoside monophosphate metabolic process	17	4	.26	15.41	2.43E-04	4.91E-02
ribonucleoside monophosphate metabolic process	26	6	.40	15.12	6.99E-06	7.79E-03
ribonucleoside monophosphate biosynthetic process	23	6	.35	17.09	3.81E-06	5.65E-03
pyrimidine nucleotide biosynthetic process	18	4	.27	14.56	2.93E-04	5.22E-02
pyrimidine nucleotide metabolic process	18	4	.27	14.56	2.93E-04	5.02E-02
pyrimidine-containing compound metabolic process	32	5	.49	10.24	2.15E-04	5.33E-02
nucleotide biosynthetic process	96	9	1.47	6.14	2.82E-05	1.80E-02
pyrimidine-containing compound biosynthetic process	19	4	.29	13.79	3.51E-04	5.58E-02
peptidyl-proline modification	34	5	.52	9.63	2.77E-04	5.37E-02
Unclassified	5351	53	81.68	.65	2.54E-05	2.27E-02
**ATG9¯/16¯**	**# *Dictyostelium***	**# Experiment**	**Expected**	**Fold Enrichment**	**P value**	**FDR**
oxidation-reduction process	563	36	14.69	2.45	1.57E-06	2.33E-03
metabolic process	3721	135	97.12	1.39	1.34E-05	1.49E-02
Unclassified	5351	88	139.66	.63	6.41E-09	1.43E-05

Molecular Function – up-regulated proteins

**ATG9¯**	***# Dictyostelium***	**# Experiment**	**Expected**	**Fold Enrichment**	**P value**	**FDR**
No statistically significant results						
**ATG16¯**	***# Dictyostelium***	**# Experiment**	**Expected**	**Fold Enrichment**	**P value**	**FDR**
No statistically significant results						
**ATG9¯/16¯**	***# Dictyostelium***	**# Experiment**	**Expected**	**Fold Enrichment**	**P value**	**FDR**
acetyl-CoA:L-glutamate N-acetyltransferase activity	3	3	.08	36.44	3.56E-04	3.83E-02
catalytic activity	3328	143	91.33	1.57	3.66E-09	1.65E-06
acyl-CoA oxidase activity	10	4	.27	14.58	4.05E-04	3.98E-02
oxidoreductase activity	507	34	13.91	2.44	3.51E-06	5.28E-04
G-protein beta/gamma-subunit complex binding	15	6	.41	14.58	1.34E-05	1.69E-03
protein-containing complex binding	218	23	5.98	3.84	1.26E-07	3.56E-05
binding	3928	165	107.80	1.53	3.57E-10	2.01E-07
G protein-coupled receptor binding	13	5	.36	14.01	8.50E-05	9.59E-03
protein binding	1152	63	31.62	1.99	2.80E-07	7.02E-05
carboxylic acid binding	32	8	.88	9.11	8.71E-06	1.16E-03
organic acid binding	32	8	.88	9.11	8.71E-06	1.23E-03
small molecule binding	1340	62	36.77	1.69	6.79E-05	8.07E-03
anion binding	1351	68	37.08	1.83	1.27E-06	2.20E-04
ion binding	2195	109	60.24	1.81	3.33E-10	2.50E-07
actin filament binding	116	15	3.18	4.71	2.14E-06	3.45E-04
actin binding	168	21	4.61	4.55	3.32E-08	1.25E-05
cytoskeletal protein binding	251	23	6.89	3.34	1.24E-06	2.33E-04
phospholipid binding	91	10	2.50	4.00	3.61E-04	3.71E-02
lipid binding	153	18	4.20	4.29	7.25E-07	1.64E-04
phosphoric ester hydrolase activity	170	14	4.67	3.00	4.24E-04	3.99E-02
metal ion binding	1059	58	29.06	2.00	7.98E-07	1.64E-04
cation binding	1079	61	29.61	2.06	1.24E-07	3.99E-05
Unclassified	5426	78	148.91	.52	5.29E-15	1.19E-11

Molecular Function – down-regulated proteins

**ATG9⁻**	***# Dictyostelium***	**# Experiment**	**Expected**	**Fold Enrichment**	**P value**	**FDR**
fatty acid elongase activity	7	3	.02	> 100	3.70E-06	8.36E-03
fatty acid synthase activity	9	3	.03	> 100	6.76E-06	2.54E-03
very-long-chain 3-ketoacyl-CoA synthase activity	7	3	.02	> 100	3.70E-06	4.18E-03
3-oxo-lignoceronyl-CoA synthase activity	7	3	.02	> 100	3.70E-06	2.79E-03
3-oxo-cerotoyl-CoA synthase activity	7	3	.02	> 100	3.70E-06	2.09E-03
3-oxo-arachidoyl-CoA synthase activity	7	3	.02	> 100	3.70E-06	1.67E-03
**ATG16⁻**	***# Dictyostelium***	**# Experiment**	**Expected**	**Fold Enrichment**	**P value**	**FDR**
acid phosphatase activity	14	4	.21	18.72	1.28E-04	4.14E-02
phosphoric ester hydrolase activity	170	12	2.59	4.62	1.94E-05	8.78E-03
hydrolase activity, acting on ester bonds	403	20	6.15	3.25	5.83E-06	4.39E-03
phospholipase activity	36	6	.55	10.92	3.57E-05	1.34E-02
lipase activity	41	7	.63	11.19	6.81E-06	3.84E-03
Unclassified	5426	48	82.82	.58	3.15E-07	3.55E-04
**ATG9⁻/16⁻**	***# Dictyostelium***	**# Experiment**	**Expected**	**Fold Enrichment**	**P value**	**FDR**
glutathione transferase activity	16	6	.42	14.37	1.37E-05	5.15E-03
catalytic activity	3328	141	86.86	1.62	2.76E-10	2.08E-07
serine-type exopeptidase activity	15	5	.39	12.77	1.17E-04	2.93E-02
disulfide oxidoreductase activity	17	5	.44	11.27	1.90E-04	4.30E-02
oxidoreductase activity	507	34	13.23	2.57	1.71E-06	9.66E-04
cis-trans isomerase activity	28	6	.73	8.21	1.91E-04	3.91E-02
calcium ion binding	107	11	2.79	3.94	2.07E-04	3.89E-02
metal ion binding	1059	52	27.64	1.88	1.46E-05	4.71E-03
cation binding	1079	53	28.16	1.88	1.13E-05	5.09E-03
ion binding	2195	87	57.29	1.52	5.20E-05	1.47E-02
Unclassified	5426	74	141.61	.52	1.74E-14	3.93E-11

Cellular Compoment – up-regulated proteins

**ATG9^{-}**	**# *Dictyostelium***	**# Experiment**	**Expected**	**Fold Enrichment**	**P value**	**FDR**
No statistically significant results						
**ATG16^{-}**	**# *Dictyostelium***	**# Experiment**	**Expected**	**Fold Enrichment**	**P value**	**FDR**
phagocytic vesicle	352	20	6.63	3.02	1.91E-05	9.47E-03
endocytic vesicle	371	21	6.98	3.01	1.24E-05	1.23E-02
**ATG9^{-}/16^{-}**	**# *Dictyostelium***	**# Experiment**	**Expected**	**Fold Enrichment**	**P value**	**FDR**
heterotrimeric G-protein complex	15	5	.41	12.15	1.47E-04	6.65E-03
extrinsic component of cytoplasmic side of plasma membrane	15	5	.41	12.15	1.47E-04	7.31E-03
cytoplasmic side of plasma membrane	19	5	.52	9.59	3.69E-04	1.31E-02
cytoplasmic side of membrane	22	5	.60	8.28	6.57E-04	2.04E-02
side of membrane	32	6	.88	6.83	4.66E-04	1.54E-02
cellular anatomical entity	6420	231	176.19	1.31	6.12E-09	7.60E-07
membrane	3101	123	85.10	1.45	7.13E-06	5.45E-04
plasma membrane	512	44	14.05	3.13	8.68E-11	1.73E-08
cell periphery	620	52	17.02	3.06	3.17E-12	7.87E-10
extrinsic component of plasma membrane	16	5	.44	11.39	1.89E-04	7.82E-03
intracellular	4444	181	121.96	1.48	1.93E-10	3.19E-08
plasma membrane protein complex	46	9	1.26	7.13	1.34E-05	8.89E-04
membrane protein complex	315	21	8.64	2.43	3.75E-04	1.29E-02
GTPase complex	15	5	.41	12.15	1.47E-04	6.96E-03
autophagosome	16	4	.44	9.11	1.73E-03	4.40E-02
membrane-bounded organelle	3235	121	88.78	1.36	1.81E-04	7.81E-03
organelle	3547	138	97.34	1.42	3.98E-06	3.30E-04
intracellular organelle	3489	135	95.75	1.41	8.49E-06	6.03E-04
cytoplasm	3227	156	88.56	1.76	1.44E-14	1.43E-11
peroxisome	63	11	1.73	6.36	3.91E-06	3.53E-04
microbody	63	11	1.73	6.36	3.91E-06	3.89E-04
phagocytic cup	37	6	1.02	5.91	9.20E-04	2.54E-02
actin cytoskeleton	141	21	3.87	5.43	2.06E-09	2.92E-07
cytoskeleton	310	26	8.51	3.06	1.18E-06	1.30E-04
plasma membrane bounded cell projection	85	10	2.33	4.29	2.19E-04	8.37E-03
cell projection	110	13	3.02	4.31	2.45E-05	1.43E-03
cell leading edge	91	10	2.50	4.00	3.61E-04	1.33E-02
cell cortex	177	17	4.86	3.50	1.77E-05	1.10E-03
mitochondrial inner membrane	165	15	4.53	3.31	9.75E-05	5.38E-03
organelle inner membrane	171	15	4.69	3.20	1.41E-04	7.36E-03
organelle membrane	704	35	19.32	1.81	9.06E-04	2.57E-02
organelle envelope	285	19	7.82	2.43	7.76E-04	2.34E-02
envelope	287	19	7.88	2.41	8.08E-04	2.36E-02
mitochondrial membrane	198	16	5.43	2.94	2.05E-04	8.15E-03
mitochondrial envelope	215	16	5.90	2.71	4.83E-04	1.55E-02
mitochondrion	546	29	14.98	1.94	1.25E-03	3.27E-02
endomembrane system	788	38	21.63	1.76	1.08E-03	2.90E-02
Unclassified	4981	72	136.70	.53	3.13E-13	1.56E-10

Cellular Compoment – down-regulated proteins

ATG9¯	# *Dictyostelium*	# Experiment	Expected	Fold Enrichment	P value	FDR
integral component of endoplasmic reticulum membrane	59	4	.19	20.91	4.87E-05	4.85E-02
intrinsic component of endoplasmic reticulum membrane	59	4	.19	20.91	4.87E-05	2.42E-02
**ATG16¯**	**# *Dictyostelium***	**# Experiment**	**Expected**	**Fold Enrichment**	**P value**	**FDR**
No statistically significant results						
**ATG9¯/16¯**	**# *Dictyostelium***	**# Experiment**	**Expected**	**Fold Enrichment**	**P value**	**FDR**
cell surface	32	6	.84	7.18	3.59E-04	2.75E-02
extracellular space	70	9	1.83	4.93	1.72E-04	1.71E-02
extracellular region	401	27	10.47	2.58	1.34E-05	1.67E-03
phagocytic vesicle	352	39	9.19	4.25	2.13E-13	2.12E-10
endocytic vesicle	371	39	9.68	4.03	9.69E-13	4.82E-10
cytoplasmic vesicle	625	47	16.31	2.88	2.25E-10	7.46E-08
intracellular vesicle	626	47	16.34	2.88	2.37E-10	5.88E-08
vesicle	645	47	16.83	2.79	5.98E-10	1.19E-07
membrane-bounded organelle	3235	114	84.43	1.35	3.49E-04	2.89E-02
organelle	3547	122	92.57	1.32	5.14E-04	3.41E-02
intracellular	4444	147	115.99	1.27	5.62E-04	3.49E-02
cytoplasm	3227	117	84.22	1.39	9.37E-05	1.03E-02
extracellular matrix	172	14	4.49	3.12	2.89E-04	2.61E-02
vacuole	225	16	5.87	2.72	4.51E-04	3.20E-02
Unclassified	4981	89	130.00	.68	3.35E-06	5.55E-04

Table S11. List of common entities of RNA_{seq} and TMT proteomics.

Strain	UniProt ID	dictyBase ID	Gene Product	FC RNA	FC protein
ATG9^{-}	Q86AA1	DDB_G0274291	Probable T4-type lysozyme 2 (lyT2-4)	5.47	2.16
	Q86ID4	DDB_G0275487	Uncharacterized protein	9.79	2.11
	Q54C11	DDB_G0293202	TNF receptor-associated factor family protein (trafH)	10.27	1.72
	Q54DL7	DDB_G0292188	von Willebrand factor A domain-containing protein	9.08	1.68
	Q552D6	DDB_G0276219	Uncharacterized protein	8.23	1.60
	Q54QE9	DDB_G0283911	heat shock protein 69 (hsp69)	3.82	1.54
	Q54G64	DDB_G0290377	Argonaut-like protein (agnB)	5.15	1.49
	Q54P57	DDB_G0284779	Uncharacterized protein	5.15	1.45
	Q54H71	DDB_G0289675	[Protein ADP-ribosylarginine] hydrolase (adprh)	4.91	1.44
	Q54CW8	DDB_G0292652	Uncharacterized protein	6.61	1.44
	Q54H65	DDB_G0289681	Uncharacterized protein	8.14	1.44
	Q54H60	DDB_G0283911	Uncharacterized protein	7.03	1.43
	Q75JI6	DDB_G0276097	putative transmembrane protein	11.76	1.43
	Q55F86	DDB_G0268208	Uncharacterized protein	3.87	1.42
	Q54FB4	DDB_G0290975	highly similar to cinB (99% identity)	2.11	1.39
	Q55DY0	DDB_G0269482	VWFA domain-containing protein	9.34	1.35
	Q54H89	DDB_G0289641	Uncharacterized protein	2.31	1.35
	Q6B9X6	DDB_G0268144	Alpha-protein kinase (vwkA)	3.39	1.34
	Q86I43	DDB_G0275161	Uncharacterized protein	2.91	1.29
	Q86AC9	DDB_G0276361	Uncharacterized protein	2.87	1.29
	Q54WL1	DDB_G0279571	Uncharacterized protein	2.12	1.27
	Q54DM8	DDB_G0292120	RBR-type E3 ubiquitin transferase (cnrK)	2.03	1.25
	Q54WZ2	DDB_G0279191	Vacuolin-B (vacB)	2.08	1.24
	Q54WE3	DDB_G0279707	Induced after Legionella Infection (iliP)	3.52	1.23
	Q8STF9	DDB_G0269202	gp64 and disintegrin-like, cysteine-rich protein (gdcA)	2.06	1.21
	P14326	DDB_G0291121	Vegetative-specific protein H5 (cinB)	2.21	1.21
	P19198	DDB_G0272560	cAMP-binding protein 1 (capA-1)	2.09	1.21
	Q75JW5	DDB_G0272182	Uncharacterized protein	2.68	1.20
	Q54FF6	*DDB_G0290887*	*Uncharacterized protein*	*0.43*	*0.74*
ATG16^{-}	Q55F86	DDB_G0268208	Uncharacterized protein	8.95	3.19
	Q54LV6	DDB_G0286393	Uncharacterized protein	2.03	2.63
	Q966R0	DDB_G0272827	Calcium-binding protein I (cbpI)	4.74	2.34
	Q54G11	DDB_G0290491	Autophagy-related protein 8-like protein (atg8b)	2.15	2.17
	P11872	DDB_G0271666	cAMP-regulated M3R protein (prtB)	3.30	1.88
	Q86AA1	DDB_G0274291	Probable T4-type lysozyme 2 (lyT2-4)	5.60	1.88
	Q555C4	DDB_G0274655	rigA	2.50	1.88
	Q54HW8	DDB_G0289171	Uncharacterized protein	3.24	1.64
	Q86L41	DDB_G0272769	Uncharacterized protein	3.64	1.57
	Q558Z0	DDB_G0272867	Probable arginine--tRNA ligase, cytoplasmic (argS1)	3.28	1.53
	Q54C11	DDB_G0293202	TNF receptor-associated factor family protein (trafH)	12.03	1.51
	Q54RB1	DDB_G0283281	strictosidine synthase family protein	2.62	1.48
	Q54QE9	DDB_G0283911	heat shock protein 69 (hsp69)	3.19	1.47
	Q1ZXM2	DDB_G0273013	Uracil-DNA glycosylase (uglB)	2.40	1.44
	Q555E4	DDB_G0267426	Uncharacterized protein	2.03	1.41
	Q7KWQ0	DDB_G0272746	Uncharacterized protein	2.19	1.41
	Q95ZG5	DDB_G0273051	Putative RNase III (drnA-1)	3.74	1.41
	Q54H65	DDB_G0289681	Uncharacterized protein	7.62	1.40
	Q54U83	DDB_G0281219	Nucleoside diphosphate-linked moiety X motif 6	2.00	1.39
	Q7KWM9	DDB_G0272738	Probable ribonucleoside hydrolase (iunH)	2.22	1.38
	Q54WL1	DDB_G0279571	Uncharacterized protein	2.79	1.38
	P19198	DDB_G0272560	cAMP-binding protein 1 (capA-1)	2.64	1.36
	Q554F5	DDB_G0275209	Putative countin receptor Cnr4 (cnrD)	2.09	1.35
	Q54WE3	DDB_G0279707	Induced after Legionella Infection (iliP)	3.01	1.35
	Q75JI6	DDB_G0276097	putative transmembrane protein	12.72	1.33

**ATG16^{-}**	Q54LY4	DDB_G0286305	zinc-containing alcohol dehydrogenase (ADH)	2.25	1.33
	Q8MQU6	DDB_G0267426	Citrate synthase, peroxisomal (cshA)	4.54	1.29
	Q54V50	DDB_G0280615	Uncharacterized protein	5.86	1.28
	Q54H89	DDB_G0289641	Uncharacterized protein	2.21	1.27
	Q86IF6	DDB_G0272989	PX domain-containing protein (vps5)	2.07	1.27
	Q54H71	DDB_G0289675	[Protein ADP-ribosylarginine] hydrolase (adprh)	3.76	1.27
	Q54K28	DDB_G0287671	Uncharacterized protein	2.21	1.26
	Q559E7	DDB_G0272955	Uncharacterized protein	2.80	1.25
	Q54JV1	DDB_G0287685	Elongation factor 2 (cinC)	2.05	1.25
	Q54P57	DDB_G0284779	Uncharacterized protein	5.22	1.24
	Q557I5	DDB_G0273537	Uncharacterized protein	2.24	1.23
	Q54DL7	DDB_G0292188	von Willebrand factor A domain-containing protein	7.50	1.23
	Q54LE9	DDB_G0286673	Uncharacterized protein	2.55	1.23
	Q86KZ6	DDB_G0272322	Uncharacterized protein	2.03	1.22
	Q557D2	DDB_G0273131	Glucose-6-phosphate 1-dehydrogenase (g6pd-1)	2.07	1.22
	Q86A16	DDB_G0272680	protein phosphatase 2C	2.04	1.22
	Q558S4	DDB_G0272841	acid phosphatase	2.30	1.21
	P54658	DDB_G0272819	32 kDa heat shock protein (hspC)	2.12	1.21
	Q54G64	DDB_G0290377	Argonaut-like protein (agnB)	4.66	1.21
	Q54IR6	DDB_G0288573	Uncharacterized protein	14.34	1.20
	Q558Z1	DDB_G0272995	Uncharacterized protein	2.20	1.20
	O15736	*DDB_G0275323*	*Protein tipD (atg16)*	*0.22*	*0.53*
	C7G078	*DDB_G0295801*	*B_lectin domain-containing protein*	*0.34*	*0.55*
	C7G077	*DDB_G0295799*	*Uncharacterized protein*	*0.28*	*0.62*
	Q7Z1Z9	*DDB_G0289883*	*Calcium up-regulated protein G (cupG)*	*0.48*	*0.67*
	Q555N6	*DDB_G0274705*	*putative glutathione S-transferase*	*0.27*	*0.68*
	Q54PD4	*DDB_G0284629*	*Uncharacterized protein*	*0.45*	*0.70*
	Q54UR0	*DDB_G0280881*	*putative glutathione S-transferase*	*0.30*	*0.70*
	Q54NR8	*DDB_G0285013*	*Uncharacterized protein*	*0.45*	*0.79*
	Q54TD6	*DDB_G0281843*	*Uncharacterized protein*	*0.50*	*0.82*
**ATG9^{-}/16^{-}**	Q54FX8	DDB_G0290655	Uncharacterized protein	2.39	1.90
	Q54IR6	DDB_G0288573	Uncharacterized protein	8.98	1.72
	Q54JV1	DDB_G0287685	Elongation factor 2 (cinC)	2.63	1.45
	Q86IG9	DDB_G0272694	Rho GTPase-activating protein (gacH)	2.06	1.43
	Q966R0	DDB_G0272827	Calcium-binding protein I (cbpI)	2.67	1.40
	Q55GM3	DDB_G0267610	NAD-dependent epimerase/dehydratase family protein	2.57	1.35
	Q553U5	DDB_G0275179	adenosine deaminase-related growth factor	2.04	1.33
	Q86A16	DDB_G0272680	protein phosphatase 2C	2.07	1.33
	Q558Z0	DDB_G0272867	Probable arginine--tRNA ligase, cytoplasmic (argS1)	2.38	1.33
	Q558Y5	DDB_G0272997	Uncharacterized protein	2.09	1.31
	Q86KF9	DDB_G0272522	Sphingosine kinase A (sgkA)	2.33	1.26
	Q54XG9	DDB_G0278975	Uncharacterized protein	2.28	1.25
	Q86B07	DDB_G0272785	acyloxyacyl hydrolase	2.96	1.24
	Q86L51	DDB_G0272857	Ras-related protein (rapB)	2.06	1.23
	Q7KWQ0	DDB_G0272746	Uncharacterized protein	2.04	1.23
	Q86IL5	DDB_G0272668	Serine/threonine-protein phosphatase 4 regulatory subunit 2 (ppr2)	2.19	1.22
	Q86JC1	DDB_G0272666	putative ankyrin repeat protein	2.17	1.21
	O15736	*DDB_G0275323*	*Protein tipD (atg16)*	*0.21*	*0.52*
	C7G078	*DDB_G0295801*	*B_lectin domain-containing protein*	*0.39*	*0.55*
	Q1ZXH5	*DDB_G0279681*	*calcium-binding EF-hand domain-containing protein*	*0.44*	*0.57*
	P54653	*DDB_G0267456*	*Calcium-binding protein 2 (cbp2)*	*0.49*	*0.65*
	Q54HF4	*DDB_G0289529*	*Uncharacterized protein*	*0.30*	*0.66*
	Q55EX9	*DDB_G0268948*	*putative SAM dependent methyltransferase*	*0.49*	*0.67*
	Q54NT1	*DDB_G0284999*	*Uncharacterized protein*	*0.26*	*0.72*

Order is based on FC of protein. Down-regulated gene products are in italic; blue characters, 14 common up-regulated entities of the ATG9^{-} and ATG16^{-} strains; red characters, 6 common up-regulated entities of the ATG16^{-} and ATG9^{-}/16^{-} strains; green characters, 2 common down-regulated entities of the ATG16^{-} and ATG9^{-}/16^{-} strains. FC, Fold change; Fold change is rounded at two places after the decimal point.
